# Asymmetric syntheses of three-membered heterocycles using chiral amide-based ammonium ylides†
†Electronic supplementary information (ESI) available: Experimental and computational details, characterization of new compounds, and copies of NMR spectra. CCDC 1023711 and 1030561. For ESI and crystallographic data in CIF or other electronic format see DOI: 10.1039/c4ob02318h



**DOI:** 10.1039/c4ob02318h

**Published:** 2014-12-18

**Authors:** Mathias Pichler, Johanna Novacek, Raphaël Robiette, Vanessa Poscher, Markus Himmelsbach, Uwe Monkowius, Norbert Müller, Mario Waser

**Affiliations:** a Institute of Organic Chemistry , Johannes Kepler University Linz , Altenbergerstraße 69 , 4040 Linz , Austria . Email: Mario.waser@jku.at ; Fax: +43 732 2468 8747 ; Tel: +43 732 2468 8748; b Institute of Condensed Matter and Nanosciences , Université catholique de Louvain , Place Louis Pasteur 1 box L4.01.02 , 1348 Louvain-la-Neuve , Belgium; c Institute of Analytical Chemistry , Johannes Kepler University Linz , Altenbergerstraße 69 , 4040 Linz , Austria; d Institute of Inorganic Chemistry , Johannes Kepler University Linz , Altenbergerstraße 69 , 4040 Linz , Austria

## Abstract

The use of carbonyl-stabilised ammonium ylides to access chiral glycidic amides and the corresponding aziridines has so far been limited to racemic *trans*-selective protocols. We herein report the development of an asymmetric approach to access such compounds with high levels of stereoselectivity using easily accessible chiral auxiliary-based ammonium ylides. The use of phenylglycinol as the chiral auxiliary was found to be superior to Evans or pseudoephedrine-based auxiliaries resulting in good to excellent stereoselectivities in both, epoxidation and aziridination reactions.

## Abstract 2

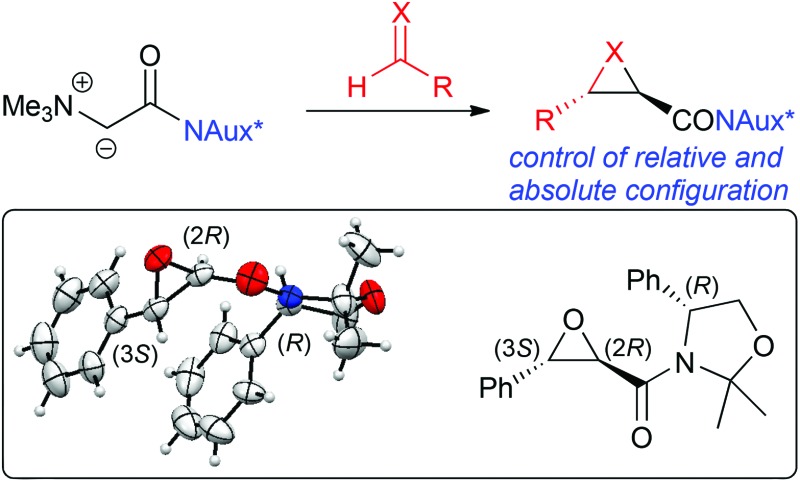
Phenylglycinol serves as a powerful chiral auxiliary in ammonium ylide-mediated reactions to obtain chiral epoxides/aziridines with excellent stereoselectivities.

## Introduction

The use of onium ylides has emerged as a powerful strategy for (dia)-stereoselective epoxide, aziridine, and cyclopropane syntheses.^1,2^ Whereas sulfonium ylides have been very frequently used for a variety of often very selective three-ring forming reactions,^3,4^ easily available ammonium ylides have been less routinely employed in the past.^5–9^ Especially the use of chiral amines to render such reactions enantioselective has been mainly limited to cyclopropanations^5^ so far. Indeed, their use in asymmetric epoxidation and aziridination reactions was found to be rather difficult, mainly due to the weaker leaving group ability of the amine group compared to the use of sulfonium ylides.^6,7,9^

**Scheme 1:**
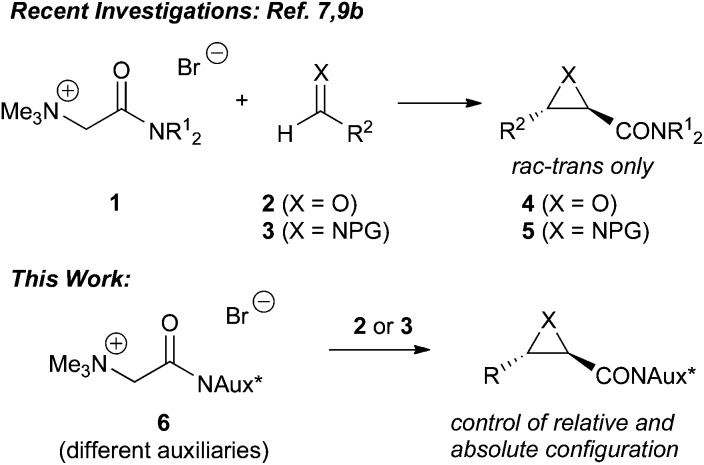
Recently developed racemic *trans*-epoxidation and aziridination protocol and the targeted auxiliary-based stereoselective approach.

We have recently introduced a highly *trans*-selective protocol for the synthesis of glycidic amides and the corresponding aziridines by reacting amide-stabilised achiral ammonium ylide precursors **1** with aldehydes **2** or imines **3** (Scheme 1).^7,9b^ Key to high yields was the use of trimethylamine as the amine leaving group, whereas the use of sterically more demanding chiral amines like Cinchona alkaloids did not result in any product formation. Due to this limitation, alternative strategies to control the absolute stereochemistry in these epoxide and aziridine forming reactions are necessary. The use of chiral auxiliary containing amides to control the face selectivity in enolate reactions is a very commonly employed and powerful strategy for numerous applications.^10^ A few examples about their use in sulfonium ylide-mediated reactions have been reported in the past^11^ but the use of chiral amide-based ammonium ylides has, to the best of our knowledge, not been reported yet. We have therefore undertaken a systematic study about the use of different classes of chiral auxiliaries to access chiral ammonium ylide precursors **6** and explore their potential in asymmetric three-membered ring heterocycle-forming reactions (Scheme 1). The main focus was on the development of a protocol for epoxidation reactions first, followed by a proof of concept for asymmetric aziridinations.

## Results and discussion

**Scheme 2:**
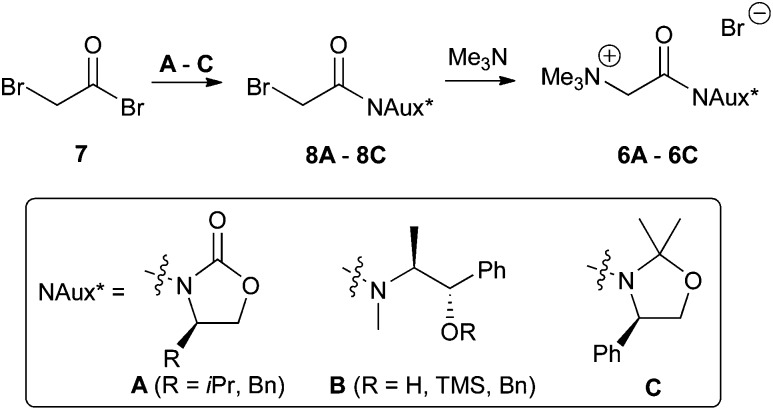
Tested chiral ammonium salts **6**.

Our main focus was on three different classes of auxiliaries: α-amino acid based oxazolidinones (Evans auxiliaries)^12^ to obtain ammonium salts **6A**, pseudoephedrine^13^-derived ammonium salts **6B**, and α-amino acid based 1,3-oxazolidine-containing salt **6C**. Synthesis of all three classes could be straightforwardly achieved by reacting bromoacetyl bromide **7** with the auxiliaries first, followed by treatment of the α-bromo acetamides **8** with trimethylamine (Scheme 2).

**Scheme 3:**
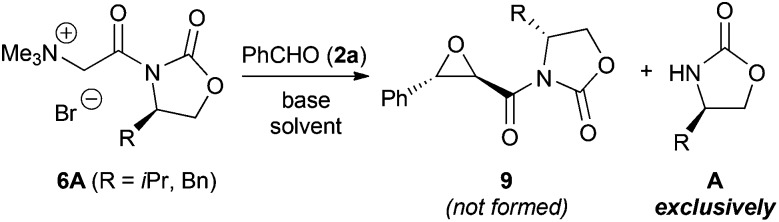
Attempted use of the Evans auxiliary containing ammonium salts **6A**.

We started testing the applicability of these ammonium salts for the asymmetric epoxidation with benzaldehyde (**2a**) first. Our recently developed protocol for the synthesis of epoxides **4** (Scheme 1) required use of a large excess of aqueous NaOH (>100 eq.).^7^ Initial experiments with ammonium salts **6A** resulted in hydrolysis of the auxiliary under these strongly basic conditions. Surprisingly, under relatively mild and weaker basic conditions, using Cs_2_CO_3_ as a solid base, full hydrolysis of the auxiliary also occurred (Scheme 3).^14^ Unfortunately, we were never able to suppress this hydrolysis of the amide bond and in no case formation of epoxide product could be observed. One possible mechanistic explanation for this extraordinary base-sensitivity of these usually rather base-stable amide motives^10,12^ may be an *in situ* ketene formation under the basic conditions. We could however not obtain experimental evidence supporting this hypothesis.

**Scheme 4:**
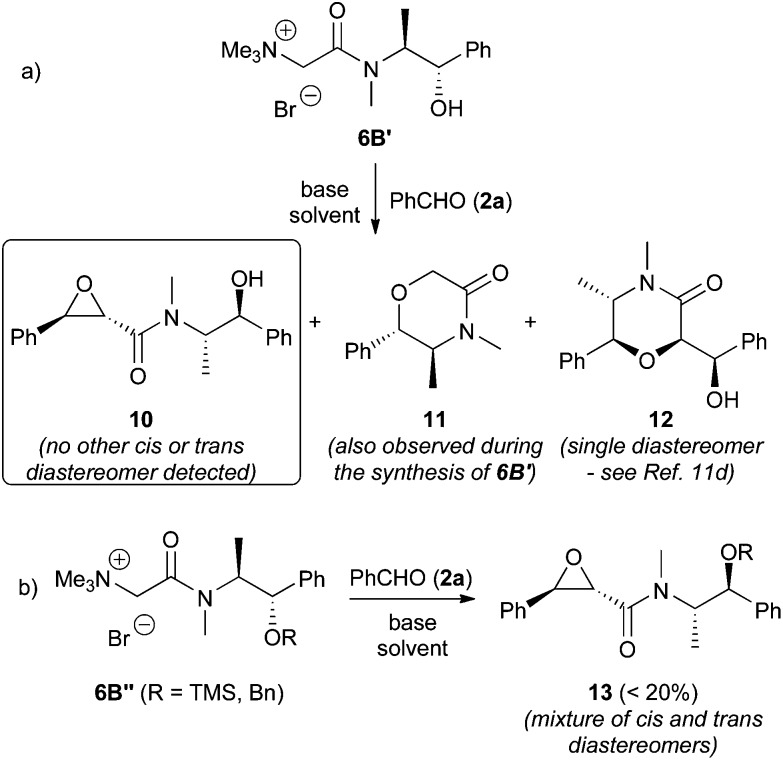
Use of the pseudoephedrine containing ammonium salts **6B**.

Next, we put our focus on the use of the pseudoephedrine based ammonium salts **6B** for the targeted epoxidation. Pseudophedrine has recently been reported as a useful auxiliary in sulfonium ylide-mediated epoxidation reactions.^11*d*^ First experiments using the free-OH containing ammonium ylide precursor **6B′** indicated formation of the target epoxide **10** as a single *trans*-diastereomer (as far as it could be judged by ^1^H NMR analysis of the crude product where **10** is present as a mixture of rotamers) and the two cyclization products **11** and **12** (Scheme 4a). Formation of **11** was also observed during the preparation of the ammonium acetamide **6B′**. Unfortunately **11** turned out to be the main product under a variety of different conditions (always at least 50%). Formation of **12** on the other hand occurs *via* a base-mediated epoxide opening of **10** as indicated by the increasing amount of **12** formed under prolonged reaction conditions. This is a known transformation which was already described by Teran *et al.* when using sulfonium ylides.^11*d*^ Interestingly, in their case no formation of **11** was observed during the epoxidation and the transformation of **10** to **12** required the use of sodium as a base in a distinct step, whereas in our experiments significant amounts of **12** were detected under either conditions (>20%). Compound **12** was always formed as a single diastereomer which fully matched the analytical data reported before.^11*d*^ This also suggests that the initial epoxide formation occurs with a high level of face differentiation. However, due to the rather fast cyclization of the starting material to give **11** and the base-mediated epoxide opening, we were not able to obtain reasonable amounts (>30%) of epoxide **10** in any case. To overcome this obstacle, we tested the O-protected ammonium salts **6B′′** (Scheme 4b). Unfortunately this turned out to be non-satisfactory as the face selectivity was found to be rather low giving a mixture of diastereomers beside significant amounts of various decomposition products (due to the initial lack of selectivity the reaction conditions were not further optimized).

**Scheme 5:**
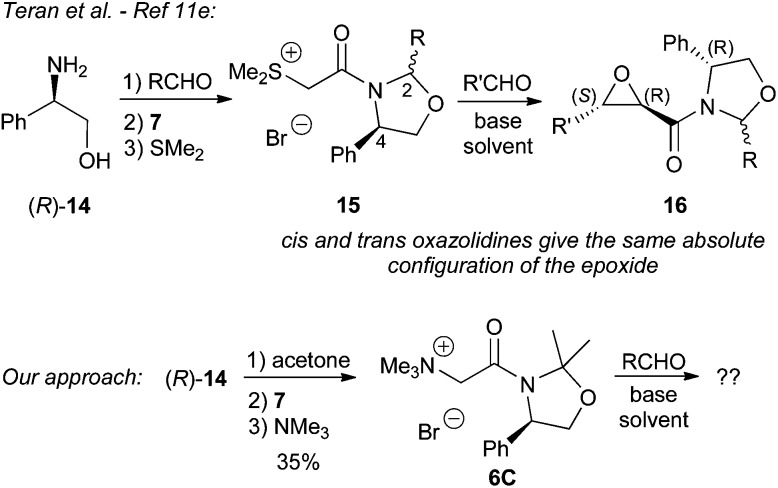
Use of phenylglycinol (**14**) to access chiral sulfonium^11*e*^ and ammonium salts for asymmetric epoxidation reactions.

Because of the limited applicability of the Evans oxazolidinones and pseudoephedrine as chiral auxiliaries for our ammonium ylide-mediated epoxidation we next turned our attention on the use of 1,3-oxazolidine-containing auxiliaries. These are less commonly employed in asymmetric transformations as compared to oxazolidinones or pseudoephedrine. However, we reasoned that the corresponding ammonium salts **6C** may work well for our target reaction as they should be stable under the basic reaction conditions and the addition step should hopefully proceed with satisfying face-selectivity too. In addition, during the initial phase of this project, Teran *et al.* reported the use of chiral 1,3-oxazolidines (derived from the reaction of phenylglycinol (**14**) with different aldehydes) in sulfur ylide-mediated epoxidation reactions.^11*e*^ Interestingly, they found that the absolute configuration of the product **16** depends exclusively on the configuration of the phenylglycinol moiety (C4) – and not on the configuration of the newly installed stereogenic center (C2) of the auxiliary. We opted for a slightly different approach by carrying out the oxazolidine formation by reacting (*R*)-**14** with acetone, which proceeds very quickly giving the auxiliary in high yield and purity. We anticipated that this ketone-based 1,3-oxazolidine should show a higher stability under basic conditions than aldehyde-based ones and should therefore be suitable for our approach. The ylide precursor **6C** could then easily be obtained in two more steps (Scheme 5).

In the initial epoxidation experiment of **6C** with benzaldehyde (**2a**) in dichloromethane (using 6 eq. of Cs_2_CO_3_ as a solid base) epoxide **17a** was formed as a single *trans*-diastereomer. Based on this promising result a systematic screening of different conditions was undertaken (Table 1 gives an overview about the most significant results obtained herein). In our attempts to determine the best-suited base and the optimum stoichiometric ratio we found that the strongly basic aqueous conditions that worked well in our racemic protocol^7^ did not give any product herein (entry 2). The use of K_2_CO_3_ (entry 3) or an equimolar amount of Cs_2_CO_3_ (entry 4) yielded also almost no product. However, an excess of Cs_2_CO_3_ and two equivalents of aldehyde allowed us to obtain *trans*-**17a** in 50% isolated yield after 24 h and in 74% after 72 h reaction time (entries 5 and 6). As expected from our previous experience using amide-stabilised ammonium ylides^7^ no *cis*-epoxide was formed under either conditions. In addition, also no second diastereoisomeric *trans*-epoxide could be observed in NMR spectra of the crude reaction mixture, illustrating that this chiral auxiliary leads to a high face-differentiation in the ylide addition step. Further screening of different solvents showed that toluene and i-PrOH can also be used as solvents for this reaction. Whereas the reaction was found to be slow in toluene, requiring either a prolonged reaction time (entry 9) or a higher reaction temperature (entry 10) to obtain **17a** in good yield, reactions in i-PrOH were usually significantly faster (entry 12). However, hereby also, significant amounts of Cannizzaro disproportionation products of benzaldehyde could be detected whereas this side reaction is less pronounced in toluene. In addition, we also found that these reactions can be accelerated by ultrasonication, giving **17a** in comparable quality and yield after 3 h (entries 11 and 13). Accordingly, this screening allowed us to identify different conditions which all gave the target epoxide **17a** with very high diastereoselectivity and with isolated yields >70%.

**Table 1:** Identification of the optimum reaction conditions for the epoxidation using the chiral ammonium ylide precursor **6C**

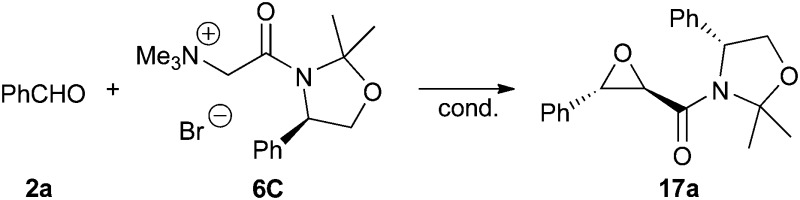							
Entry	**2a** (eq.)	Solv.	Base (eq.)	*T* [°C]	*t* [h]	Yield^*a*^ [%]	dr^*b*^ [%] (*trans*)^*c*^
1	4	CH_2_Cl_2_	Cs_2_CO_3_ (6×)	25	24	35	>98
2	2	CH_2_Cl_2_	NaOH (aq) (140×)	25	24	n.r.	n.d.
3	2	CH_2_Cl_2_	K_2_CO_3_ (20×)	25	24	n.r.	n.d.
4	1	CH_2_Cl_2_	Cs_2_CO_3_ (1×)	25	24	<10	>98
5	2	CH_2_Cl_2_	Cs_2_CO_3_ (20×)	25	24	50^*d*^	>98
6	2	CH_2_Cl_2_	Cs_2_CO_3_ (20×)	25	72	74	>98
7	2	THF	Cs_2_CO_3_ (20×)	25	24	n.r.	n.d.
8	2	Toluene	Cs_2_CO_3_ (20×)	25	24	50^*d*^	>98
9	2	Toluene	Cs_2_CO_3_ (20×)	25	72	72	>98
10	2	Toluene	Cs_2_CO_3_ (20×)	60	24	73	>98
11	2	Toluene	Cs_2_CO_3_ (20×)	60^*e*^	3	75	>98
12	2	i-PrOH	Cs_2_CO_3_ (20×)	25	24	78	>98
13	2	i-PrOH	Cs_2_CO_3_ (20×)	60^*e*^	3	84	>98

^*a*^Isolated yield.

^*b*^Determined by ^1^H NMR of the crude reaction mixture.

^*c*^In neither experiment any *cis*-diastereomer could be detected.

^*d*^Incomplete conversion of **6C**.

^*e*^Carried out in an ultrasonic bath.

**Scheme 6:**
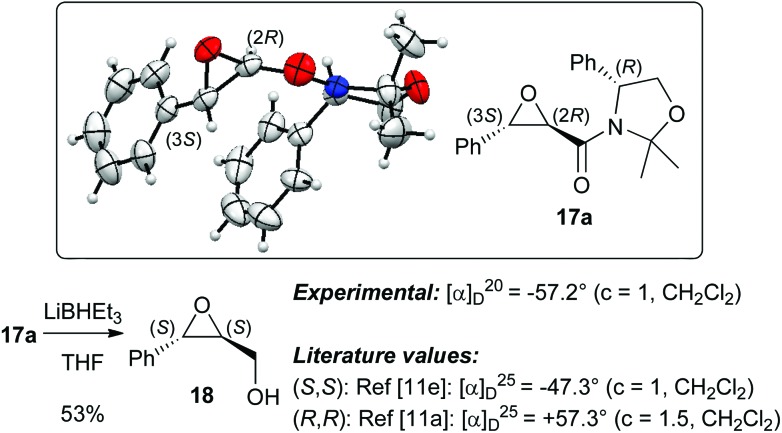
Molecular structure of **17a** and conversion into the known epoxyalcohol **18**.

The absolute configuration of **17a** was unambiguously proven by two different methods. First X-ray diffraction analysis of crystals of **17a** proved the (2*R*,3*S*)-configuration of the *trans*-epoxyamide moiety.^15^ In addition **17a** was also transferred into the known epoxyalcohol **18** upon treatment with LiBHEt_3_. Comparison of the specific optical rotation with literature values of **18** ^11a,e^ also confirmed this absolute configuration of the epoxy moiety (Scheme 6). Unfortunately, this transformation did not allow us to recover the auxiliary.

**Scheme 7:**
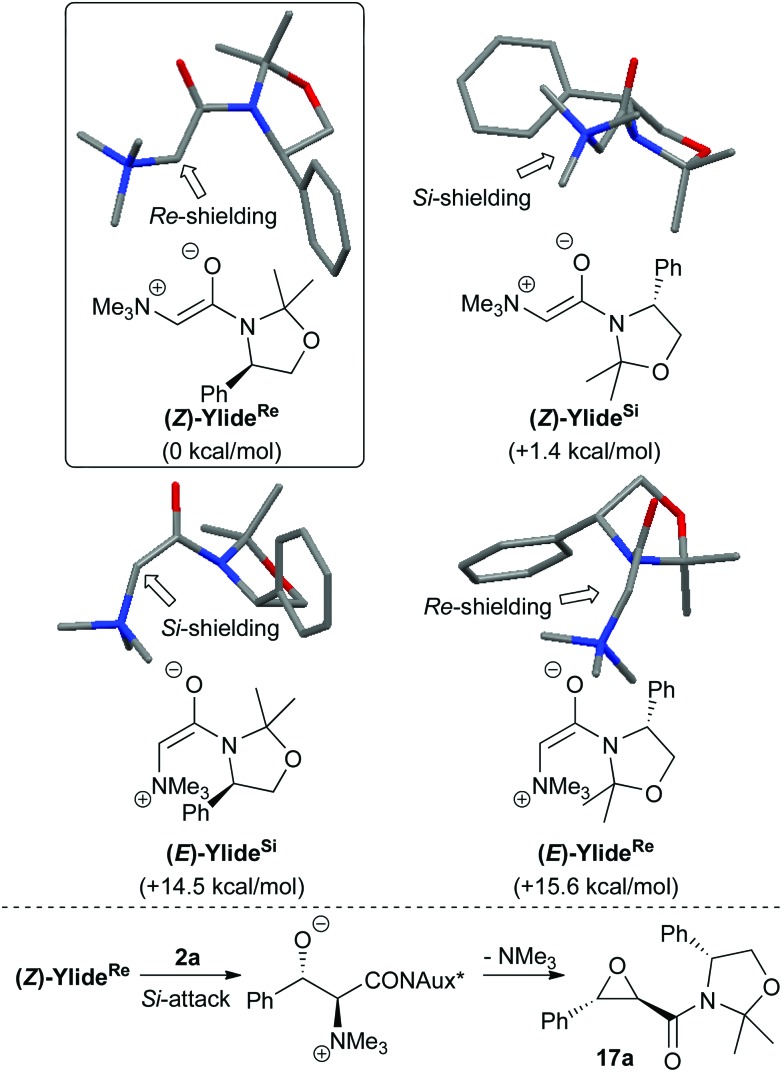
DFT calculations of the ylide geometry (relative energies given in brackets) and proposed rationale for observed diastereoselectivity.^16^

In order to get an insight into the interpretation of the observed high diastereoselectivity in all these epoxidation reactions, we undertook DFT calculations investigating the geometry of the most stable conformer of the ylide.^16,17^ It turned out that (*Z*)-ylides are significantly more stable than (*E*)-ylides (Scheme 7). This can most probably be accounted for by destabilizing steric interactions between the ammonium group and the auxiliary in the (*E*)-ylide and the presence of stabilizing electrostatic interactions between the ammonium group and the enolate oxygen in the (*Z*)-isomer.^18^ In addition, the (*Z*)-ylide in which the phenyl group blocks the *Re*-face of the α-carbon (**(*Z*)-ylide^*Re*^**) is thermodynamically more stable than the one where the *Si*-face is shielded (**(*Z*)-ylide^*Si*^**) (Scheme 7, upper part).

Based on these results, the observed stereoselectivity could be explained by addition of the most stable ylide (**(*Z*)-ylide^*Re*^**) to benzaldehyde **2a** *via* the *Si*-face (Scheme 7, lower part).^17^

With a set of high yielding reaction conditions available we next investigated the application scope of this epoxidation reaction. As shown in Table 2 a series of different aldehydes was tested. Notably, in neither case any *cis*-epoxide and no second *trans*-diastereomer could be detected. Due to the faster reaction rate of the parent benzaldehyde **2a** in i-PrOH at room temperature the majority of the reactions were carried out under these conditions (cond. A). These worked reasonably fine for a series of aromatic aldehydes such as *para*- or *ortho*-methyl benzaldehydes (entries 3 and 4), halide-substituted ones (entries 5 and 6) and the biphenyl carbaldehyde **2f** (entry 7). Also the more electron-rich *para*-methoxy benzaldehyde **2g** could be reacted in good yield under these conditions, whereas the less active dimethylamino benzaldehyde **2h** required harsher conditions (60 °C in toluene) to obtain the epoxide **17h** in good crude yield. Remarkably, this was the first time that this aldehyde could be successfully used in any of our ammonium ylide-mediated epoxidation reactions.^7^ Unfortunately, epoxide **17h** quickly decomposed during different purification methods. As expected, the more reactive cyano- and nitrobenzaldehydes **2i** and **2j** did not give any epoxide under the standard conditions (cond. A) but mainly the corresponding Cannizzaro disproportionation products. However, carrying out the reaction in toluene as a less polar solvent at room temperature allowed us to obtain the epoxides **17i** and **17j** in reasonable isolated yields. Notably, for the first time we have been able to use the aliphatic aldehyde **2k** (entry 12) to obtain the corresponding epoxide in moderate yield. Such enolisable aldehydes had primarily undergone self aldol condensation reactions under the strongly basic conditions developed previously.^7^ Now the use of Cs_2_CO_3_ in toluene allowed us to overcome this limitation to some extent. Similar yield was obtained when reacting cyclohexanecarbaldehyde (**2l**) under these conditions (entry 13).

**Table 2:** Application scope of the asymmetric epoxidation using amide **6C**

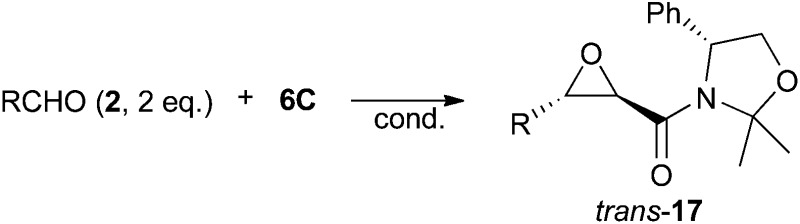						
Entry	R	Ald.	Prod.	Cond.^*a*^	Yield^*b*^ [%]	dr^*c*^ [%]
1	Ph	**2a**	**17a**	A	78	>98
2				B	73	>98
3	4-MeC_6_H_4_–	**2b**	**17b**	A	80	>98
4	2-MeC_6_H_4_–	**2c**	**17c**	A	79	>98
5	4-ClC_6_H_4_–	**2d**	**17d**	A	75	>98
6	4-BrC_6_H_4_–	**2e**	**17e**	A	85	>98
7	4-PhC_6_H_4_–	**2f**	**17f**	A	89	>98
8	4-MeOC_6_H_4_–	**2g**	**17g**	A	73	>98
9	4-Me_2_NC_6_H_4_–	**2h**	**17h**	B	(60)^*d*^	>98
10	4-CNC_6_H_4_–	**2i**	**17i**	C	62	>98
11	3-NO_2_C_6_H_4_–	**2j**	**17j**	C	68	>98
12	*n*-Decyl–	**2k**	**17k**	D	42	>98
13	Cyclohexyl–	**2l**	**17l**	D	39	>98

^*a*^A: i-PrOH, 25 °C, 24 h, Cs_2_CO_3_ (s, 20 eq.); B: toluene, 60 °C, 24 h, Cs_2_CO_3_ (s, 20 eq.); C: toluene, 25 °C, 72 h, Cs_2_CO_3_ (s, 20 eq.); D: toluene, 25 °C, 24 h, Cs_2_CO_3_ (s, 20 eq.).

^*b*^Isolated yield.

^*c*^Determined by ^1^H NMR of the crude reaction mixture.

^*d*^Full decomposition on silica gel, crude NMR yield given in brackets.

Based on the detailed knowledge obtained on the use of the chiral amide **6C** for asymmetric ylide-mediated epoxidations at hand, we carried out a short screening to test the potential of this concept for the related aziridination reaction (Table 3). In analogy to our previous experience using achiral amides **6** for racemic aziridinations^9*b*^ the use of Cs_2_CO_3_ in CH_2_Cl_2_ was also found superior here compared to the use of i-PrOH or toluene as solvents. Interestingly, the use of *N*-tosyl imine **3a** allowed the synthesis of the *trans*-aziridine **19a** in high selectivity (entry 1). Unfortunately this compound was found to be relatively unstable and partially decomposed under a variety of purification conditions. This may be attributed to the rather strained nature of this *trans*-aziridine with the bulky tosyl group being *cis* to either the phenyl group or the chiral auxiliary.^4*c*^ The use of *N*-Boc imine **3b** gave an interesting result (entry 2). Formation of the expected major *trans*-aziridine **19b** (isolated in 61% yield) was accompanied by minor amounts of the second *trans*-aziridine as well as around 10% of *cis*-**19b**. Besides this reduced stereoselectivity in the aziridine formation (compared to epoxidation) also notable amounts of the α,β-unsaturated β-amino amide **20b** were obtained. Formation of analogous olefins was already observed during our previous racemic studies but only when using electron-poor *N*-Boc benzaldimines (*e.g.* NO_2_-substituted) or heteroaromatic *N*-Boc aldimines, whereas not even traces thereof could be detected using more electron-rich imines like **3b**.^9*b*^ Unfortunately we were not able to suppress formation of **20** by changing the reaction conditions (in contrast: using less base gave a larger amount of **20**). When using more electron-rich imines **3c** and **3d** the formation of olefins **20c** and **20d** was reduced, but still not totally suppressed (entries 3 and 4). Again, formation of the minor *trans*-aziridine and *cis*-diastereomer was observed too. While the isolated yield of **19c** may be a bit lower because of a reduced rate of addition due to the *ortho*-substituent, the more electron-rich *p*-methoxy containing **19d** was found to be totally unstable on silica gel and also partially decomposed during alumina column chromatography.^19^ The naphthyl-based imine **3e** performed similarly to imine **3b** (compare entries 5 and 2) giving the major *trans*-aziridine **19e** in a reasonable isolated yield of 59%. By contrast, the less electron-rich bromine-substituted **3f** gave the olefin **20f** as the main product (entry 6), while, as expected, the electron-poor **3g** did not allow us to obtain any aziridine at all (entry 7).

**Table 3:** Asymmetric aziridination using amide **6C**

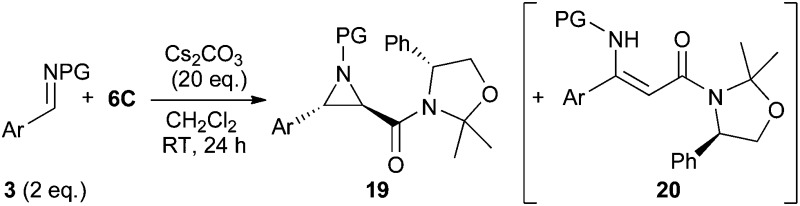						
Entry	PG	Ar	**3**	**19** (dr)^*a*^ ^,^ ^*b*^ [%]	**20** ^*a*^ [%]	Yield [%] (*trans*-**19**)^*c*^
1	Tos	Ph	**3a**	>98 *trans* ^*d*^	n.d.	39 (70)^*e*^
2	Boc		**3b**	82 (87/3/10)^*b*^	18	62
3		2-MeC_6_H_4_–	**3c**	96 (85/3/12)^*b*^	4	56
4		4-MeOC_6_H_4_–	**3d**	95 (88/3/9)^*b*^	5	57 (75)^*f*^
5		Napht-2-yl	**3e**	77 (85/3/12)^*b*^	23	58
6		4-BrC_6_H_4_–	**3f**	39 (86/0/14)^*b*^	61	32
7		3-NO_2_C_6_H_4_–	**3g**	n.d.	>99	n.d.

^*a*^Determined by ^1^H NMR of the crude reaction mixture.

^*b*^Values in brackets give the diastereomeric ratios of aziridines (*trans*_major_/*trans*_minor_/*cis*) – only one *cis*-isomer could be detected.

^*c*^Isolated yield of the major *trans*-aziridine.

^*d*^Only one *trans*-aziridine detected.

^*e*^Partial decomposition on silica gel, crude NMR yield given in brackets.

^*f*^Full decomposition on silica gel and partial on alumina, crude NMR yield given in brackets.

**Fig. 1:**
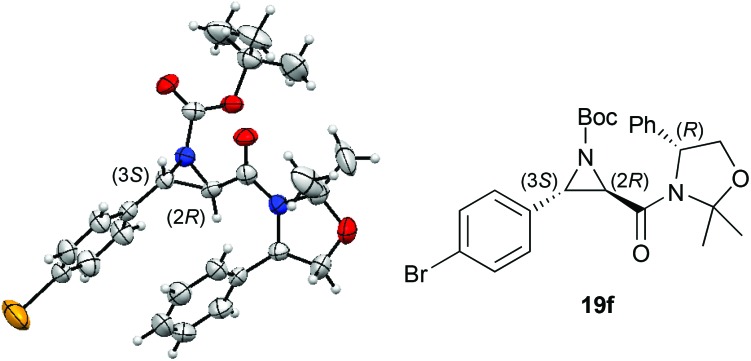
Molecular structure of **19f**.

The absolute configuration of the major *trans*-aziridine was determined by anomalous X-ray diffraction analysis of crystals of bromine-containing **19f**. Again, in analogy to the epoxidation reaction, the (2*R*,3*S*)-configuration of the *trans*-heterocyclic moiety was found for the major isomer (Fig. 1).^15^

## Experimental

### General

^1^H- and ^13^C-NMR spectra were recorded on a Bruker Avance DRX 500 MHz spectrometer, a Bruker Avance III 300 MHz spectrometer, and on a Bruker Avance III 700 MHz spectrometer with TCI cryoprobe. All NMR spectra were referenced using the residual ^1^H solvent peak as secondary reference. High resolution mass spectra were obtained using a Thermo Fisher Scientific LTQ Orbitrap XL with an Ion Max API Source. All analyses were made in the positive ionisation mode. IR spectra were recorded on a Bruker Tensor 27 FT-IR spectrometer with ATR unit. All chemicals were purchased from commercial suppliers and used without further purification unless otherwise stated. All reactions were performed under an Ar-atmosphere. CH_2_Cl_2_ was distilled over P_2_O_5_ and stored under Ar (it was not necessary to dry CH_2_Cl_2_ prior to every experiment and usually this quality could be used successfully in these reactions over the course of 3–4 weeks after distillation). Single-crystal structure analyses were carried out on a Bruker Smart X2S diffractometer operating with Mo-K_α_ radiation (*λ* = 0.71073 Å).

Geometry optimization has been performed using the Jaguar 8.0 pseudospectral program package using the well-established B3LYP hybrid density functional with the D3 dispersion correction and the standard split valence polarized 6-31G* basis as implemented in Jaguar. All the optimization calculations were carried out using the Poisson–Boltzmann polarizable continuum method as incorporated in Jaguar, and parameters for CH_2_Cl_2_. Energies were obtained by single point energy calculations at the B3LYP-D3/6-311+G**(CH_2_Cl_2_) level. The correct nature of each stationary point has been checked by performing frequency calculations at the B3LYP/6-31G*(CH_2_Cl_2_) level of theory. Thermal and entropic contributions to free energy (at 298.15 K) and zero-point energy have been obtained from these frequency calculations. We have made a systematic attempt to locate all possible local minima, with the data presented referring to the lowest energy form.

### Ammonium amide **6C**

(*R*)-**14** (3.00 g, 21.9 mmol) was dissolved in 50 mL acetone and 3.2 g anhydrous MgSO_4_ were added and the reaction mixture was stirred for 3 h at 20 °C. After filtration and evaporation to dryness the product **21** was obtained in 95% (3.68 g, 20.7 mmol) and used without further purification. The ^1^H-NMR-spectrum is in full accordance to literature.^20^ ^1^H-NMR (300 MHz, *δ*, CDCl_3_, 298 K): 1.45 (s, 3H), 1.52 (s, 3H), 2.04 (b, 1H), 3.71 (t, 1H, *J* = 7.8 Hz), 4.29 (t, 1H, *J* = 7.8 Hz), 4.54 (t, 1H, *J* = 7.8 Hz), 7.26–7.41 (m, 5H, Ar–H) ppm.

Compound **21** (3.68 g, 20.7 mmol) was dissolved in 25 mL CH_2_Cl_2_ and 83 mL aqueous saturated Na_2_CO_3_ solution were added. Then bromide **7** (2.9 mL, 21.7 mmol, 1.05 eq.) was added and the mixture was vigorously stirred for 4 h. After addition of aqueous saturated NaHCO_3_ the aqueous layer was separated and washed three times with 20 mL CH_2_Cl_2_. The combined organic phases were dried over anhydrous MgSO_4_, filtrated and the solvent removed under reduced pressure. The product was purified by column chromatography (silica gel, heptanes–EtOAc = 5 : 1) to give **8C** (2.57 g, 8.6 mmol, 41% yield) as a light-brown solid. The ^1^H-NMR-spectrum was in accordance to literature.^21^ ^1^H-NMR (300 MHz, *δ*, CDCl_3_, 298 K): 1.64 (s, 3H), 1.87 (s, 3H), 3.45 (d, 1H, *J* = 11.0 Hz), 3.52 (d, 1H, *J* = 11.0 Hz), 3.94 (dd, 1H, *J*_1_ = 9.0 Hz, *J*_2_ = 2.7 Hz), 4.41 (dd, 1H, *J*_1_ = 9.0 Hz, *J*_2_ = 6.5 Hz), 5.07 (dd, 1H, *J*_1_ = 6.5 Hz, *J*_2_ = 2.7 Hz), 7.25–7.45 (m, 5H) ppm.

Compound **8C** (2.57 g, 8.6 mmol) was dissolved in THF (26 mL) and NMe_3_ (2.50 mL, 20.4 mmol, 1.2 eq., 33% solution in EtOH) was added. After stirring for 24 h at r.t. the solvent was removed with vacuum distillation. The crude product (purity >90%) was purified by column chromatography (heptanes → CH_2_Cl_2_–MeOH = 5 : 1) to give the ammonium salt **6C** in 90% yield (2.90 g, 8.1 mmol) as a white foam. [*α*]22D (*c* = 0.6, CH_2_Cl_2_) = –92; ^1^H-NMR (700 MHz, *δ*, CDCl_3_, 298 K): 1.65 (s, 3H), 1.86 (s, 3H), 2.98 (d, 1H, *J* = 16.3 Hz), 3.44 (s, 9H), 3.91 (dd, 1H, *J*_1_ = 9.2 Hz, *J*_2_ = 1.7 Hz), 4.46 (dd, 1H, *J*_1_ = 9.2 Hz, *J*_2_ = 6.5 Hz), 5.83 (dd, 1H, *J*_1_ = 6.5 Hz, *J*_2_ = 1.7 Hz), 6.07 (d, 1H, *J* = 16.3 Hz), 7.28–7.51 (m, 5H) ppm; ^13^C NMR (176 MHz, *δ*, CDCl_3_, 298 K): 23.5, 25.5, 54.7, 60.1, 64.8, 71.8, 97.5, 126.8, 128.5, 129.5, 140.5, 161.0 ppm; IR (film): *ν̄* = 3011, 2987, 2937, 2882, 1654, 1434, 1412, 1378, 1351, 1237, 1204, 1133, 1064, 1048, 923, 896, 843, 703, 664, 604, 579, 563, 517, 501 cm^–1^; HRMS (ESI): *m*/*z* calcd for C_16_H_25_N_2_O_2_^+^: 277.1910 [M]^+^; found: 277.1904.

### General epoxidation procedure using ammonium amide **6C**

Ammonium salt **6C** was dissolved in the appropriate solvent (20 mL mmol^–1^ ammonium salt) and Cs_2_CO_3_ (20 eq.) was added to the reaction mixture. After 5 min the aldehyde (2 eq.) was added and the suspension was stirred for the indicated time at the given temperature. The reaction was quenched with water and extracted with toluene. The organic phase was washed with brine and dried with anhydrous Na_2_SO_4_, filtrated and the solvent was removed under reduced pressure. The epoxide was purified by column chromatography (silica gel, heptanes–EtOAc = 7 : 3).

#### *trans*-Epoxide **17a**

Obtained in 78% (1 mmol scale) as a white solid after column chromatography (Cond. A). [*α*]22D (*c* = 1.4, CH_2_Cl_2_) = –178; ^1^H NMR (700 MHz, *δ*, CDCl_3_, 298 K): 1.72 (s, 3H), 1.88 (s, 3H), 3.22 (d, 1H, *J* = 1.8 Hz), 3.68 (d, 1H, *J* = 1.8 Hz), 3.90 (dd, 1H, *J*_1_ = 9.2 Hz, *J*_2_ = 4.2 Hz), 4.41 (dd, 1H, *J*_1_ = 9.2 Hz, *J*_2_ = 6.6 Hz), 5.10 (dd, 1H, *J*_1_ = 6.6 Hz, *J*_2_ = 4.2 Hz), 6.78 (m, 2H), 7.00–7.20 (m, 8H) ppm; ^13^C NMR (176 MHz, *δ*, CDCl_3_, 298 K): 24.3, 25.1, 58.4, 58.9, 61.5, 72.1, 97.3, 125.9, 126.1, 128.4, 128.5, 128.7, 129.4, 135.3, 140.6, 164.2 ppm; IR (film): *ν̄* = 3032, 2989, 2933, 2873, 1658, 1458, 1437, 1387, 1363, 1252, 1205, 1081, 1066, 894, 849, 772, 749, 695, 660, 596, 552, 513 cm^–1^; HRMS (ESI): *m*/*z* calcd for C_20_H_21_NO_3_: 324.1594 [M + H]^+^; found: 324.1595.

### General procedure for the preparation of aziridines

A mixture of **6C** (0.4 mmol), aldimine **3** (2 eq.), and Cs_2_CO_3_ (20 eq.) in CH_2_Cl_2_ (8 mL) was vigorously stirred for 24 h at room temperature. CH_2_Cl_2_ and brine were added and the phases separated. The aqueous layer was extracted twice with CH_2_Cl_2_, the combined organic layers were extracted with brine and the aqueous layer was re-extracted twice with CH_2_Cl_2_. The combined organic layers were dried over Na_2_SO_4_, filtrated, evaporated, and dried *in vacuo*. Column chromatography (silica gel, heptanes–EtOAc = 20 : 1–2 : 1) gave the aziridines **19** in the reported yields. In most cases the minor *cis*-isomers and the olefins **20** could not be obtained in pure form.

#### *trans-N*-Boc aziridine **19b**

Obtained in 62% as a colourless residue. [*α*]23D (*c* = 1.6, CH_2_Cl_2_) = –110; ^1^H NMR (500 MHz, *δ*, CDCl_3_, 298 K): 1.48 (s, 9H), 1.73 (s, 3H), 1.89 (s, 3H), 2.87 (d, 1H, *J* = 2.5 Hz), 3.69 (d, 1H, *J* = 2.5 Hz), 3.96 (dd, 1H, *J* = 9.1, 3.5 Hz), 4.47 (dd, 1H, *J* = 9.1, 6.8 Hz), 5.28 (dd, 1H, *J* = 6.8, 3.5 Hz), 6.88 (d, 2H, *J* = 6.9 Hz), 7.19–7.28 (m, 8H) ppm; ^13^C NMR (125 MHz, *δ*, CDCl_3_, 298 K): 23.8, 25.0, 28.0, 44.5, 44.8, 61.5, 71.6, 81.7, 96.8, 125.7, 126.5, 127.7, 128.0, 128.1, 129.1, 135.3, 140.9, 159.2, 163.7 ppm; IR (film): *ν̄* = 3009, 2984, 2935, 2868, 1715, 1652, 1433, 1395, 1364, 1333, 1253, 1223, 1204, 1074, 1053, 819, 755, 746, 705, 691, 635 cm^–1^; HRMS (ESI): *m*/*z* calcd for C_25_H_30_N_2_O_4_: 423.2278 [M + H]^+^; found: 423.2287.

## Conclusions

The use of easily available phenylglycinol as a chiral auxiliary in ammonium ylide-mediated reactions was found to be a promising strategy to obtain chiral three-membered ring heterocycles with good to excellent stereoselectivities and in good yields. In general it was found that the epoxidation reaction is rather broad in its application scope giving glycidic amides as single stereoisomers in all cases. The aziridination is slightly less selective giving the major *trans*-isomer with at least 85% diastereoselectivity but, depending on the electronic nature of the starting imine, with varying amounts of an α,β-unsaturated β-amino amide side-product.
