# Supplementary material for: Asymmetric syntheses of three-membered heterocycles using chiral amide-based ammonium ylides
Source: Org Biomol Chem. 2014 Dec 18;13(7):2092–9. doi: 10.1039/c4ob02318h (PMC4323751; doi:10.1039/c4ob02318h)
Supplement: Supplementary file 1 [file OB-013-C4OB02318H-s001.pdf]

Electronic Supplementary Material (ESI) for Organic & Biomolecular Chemistry.
This journal is © The Royal Society of Chemistry 2014

SUPPORTING INFORMATION

Asymmetric Syntheses of Three-Membered Heterocycles Using Chiral Amide-Based Ammonium Ylides

Mathias Pichler,[a] Johanna Novacek,[a] Raphaël Robiette,[b] Vanessa Poscher,[a] Markus Himmelsbach,[c] Uwe Monkowius,[d] Norbert Müller,[a] and Mario Waser[a,*]

[a] *Institute of Organic Chemistry, Johannes Kepler University Linz, Altenbergerstraße 69, 4040 Linz, Austria. Fax: +43 732 2468 8747; Tel: +43 732 2468 8748; E-mail: Mario.waser@jku.at*

[b] *Institute of Condensed Matter and Nanosciences, Université catholique de Louvain, Place Louis Pasteur 1 box L4.01.02, 1348 Louvain-la-Neuve, Belgium.*

[c] *Institute of Analytical Chemistry, Johannes Kepler University Linz, Altenbergerstraße 69, 4040 Linz, Austria.*

[d] *Institute of Inorganic Chemistry, Johannes Kepler University Linz, Altenbergerstraße 69, 4040 Linz, Austria*

1. General Information: 2
2. Synthesis of Ammonium Amide 6C. 3
3. Syntheses of Epoxides Using Amide 6C: 4
4. Reduction of Epoxide 17a: 9
5. Syntheses of Aziridines Using Amide 6C: 10
6. DFT Calculations: 14
7. X-Ray Crystallography: 17
8. Copies of selected NMR spectra: 19

1. General Information:

Melting points were measured on a Kofler melting point microscope (Reichert, Vienna). ^{1}H- and ^{13}C-NMR spectra were recorded on a Bruker Avance III 300 MHz spectrometer, a Bruker Avance DRX 500 MHz, and on a Bruker Avance III 700 MHz spectrometer with TCI cryoprobe. All NMR spectra were referenced on the solvent peak. High resolution mass spectra were obtained using a Thermo Fisher Scientific LTQ Orbitrap XL with an Ion Max API Source. All analyses were made in the positive ionisation mode. IR spectra were recorded on a Bruker Tensor 27 FT-IR spectrometer with ATR unit. All chemicals were purchased from commercial suppliers and used without further purification unless otherwise stated. All reactions were performed under an Ar-atmosphere. CH_2Cl_2 was distilled over P_2O_5 and stored under Ar (it was not necessary to dry CH_2Cl_2 prior to every experiment and usually this quality could be used successfully in these reactions over the course of 3-4 weeks after distillation). Column chromatography was carried out using silica gel and heptanes/EtOAc or CH_2Cl_2/MeOH (different ratios) as the eluent. Flushing the column with Et_3N (1% in heptanes) prior to use was found beneficial in some cases. Starting imines[1] were prepared according to literature procedures. Single-crystal structure analyses were carried out on a Bruker Smart X2S diffractometer operating with Mo-K_α radiation (λ= 0.71073 Å). Geometry optimization has been performed using the Jaguar 8.0 pseudospectral program package using the well established B3LYP hybrid density functional with the D3 dispersion correction and the standard split valence polarized 6-31G* basis as implemented in Jaguar. All the optimization calculations were carried out using the Poisson-Boltzmann polarizable continuum method as incorporated in Jaguar, and parameters for CH_2Cl_2. Energies were obtained by single point energy calculations at the B3LYP-D3/6-311+G**(CH_2Cl_2) level. The correct nature of each stationary point has been checked by performing frequency calculations at the B3LYP/6-31G*(CH_2Cl_2) level of theory. Thermal and entropic contributions to free energy (at 298.15 K) and zero-point energy have been obtained from these frequency calculations. We have made a systematic attempt to locate all possible local minima, with the data presented referring to the lowest energy form.

[1] (a) L. Huang and W. D. Wulff, *J. Am. Chem. Soc.* 2011, **133**, 8892-8895; (b) T. Regiani, V. G. Santos, M. N. Godói, B. G. Vaz, M. N. Eberlin and F. Coelho, *Chem. Commun.* 2011, **47**, 6593-6595; (c) K. Yoshida, N. Akashi and A. Yanagisawa, *Tetrahedron: Asymmetry* 2011, **22**, 1225-1230.

2. Synthesis of Ammonium Amide 6C.

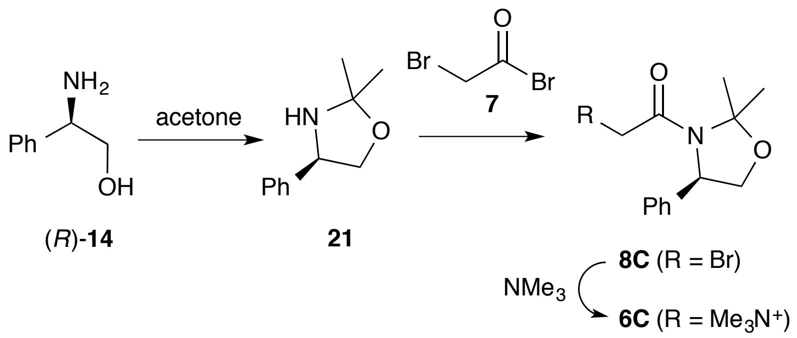

Step 1: (*R*)-**14** (3.00 g, 21.9 mmol) was dissolved in 50 mL acetone and 3.2 g anhydrous $MgSO_4$ were added and the reaction mixture was stirred for 3 h at 20 °C. After filtration and evaporation to dryness the product **21** was obtained in 95% (3.68 g, 20.7 mmol) and used without further purification. The ^{1}H-NMR-spectrum is in full accordance to literature.[2] ^{1}H-NMR (300 MHz, δ, $CDCl_3$, 298 K): 1.45 (s, 3H), 1.52 (s, 3H), 2.04 (b, 1H), 3.71 (t, 1H, J = 7.8 Hz), 4.29 (t, 1H, J = 7.8 Hz), 4.54 (t, 1H, J = 7.8 Hz), 7.26 - 7.41 (m, 5H, Ar-H) ppm.

Step 2: Compound **21** (3.68 g, 20.7 mmol, 1 eq.) was dissolved in 25 mL CH_2Cl_2 and 83 mL aqueous saturated Na_2CO_3 solution were added. Then bromide **7** (2.9 mL, 21.7 mmol, 1.05 eq.) was added and the mixture was vigorously stirred for 4 h. After addition of aqueous saturated $NaHCO_3$ the aqueous layer was separated and washed three times with 20 mL CH_2Cl_2. The combined organic phases were dried over anhydrous $MgSO_4$, filtrated and the solvent removed under reduced pressure. The product was purified by column chromatography (silica gel, heptanes:EtOAc = 5:1) to give **8C** (2.57 g, 8.6 mmol, 41 % yield) as a light-brown solid. The ^{1}H-NMR-spectrum was in accordance to literature.[3] ^{1}H-NMR (300 MHz, δ, $CDCl_3$, 298 K): 1.64 (s, 3H), 1.87 (s, 3H), 3.45 (d, 1H, J = 11.0 Hz), 3.52 (d, 1H, J = 11.0 Hz), 3.94 (dd, 1H, J_1 = 9.0 Hz, J_2 = 2.7 Hz), 4.41 (dd, 1H, J_1 = 9.0 Hz, J_2 = 6.5 Hz), 5.07 (dd, 1H, J_1 = 6.5 Hz, J_2 = 2.7 Hz), 7.25 - 7.45 (m, 5H) ppm.

Step 3: Compound **8C** (2.57 g, 8.6 mmol) was dissolved in THF (26 mL) and NMe_3 (2.50 mL, 20.4 mmol, 1.2 eq., 33 % solution in EtOH) was added. After stirring for 24 h at r.t. the solvent was removed with vacuum distillation. The crude product (purity >90%) was purified by column chromatography (heptanes → CH_2Cl_2:MeOH = 5:1) to give the ammonium salt **6C** in 90% yield (2.90 g, 8.1 mmol) as a white hygroscopic foam. $[\alpha]_D^{22}$ (c = 0.6, DCM) = -92; ^{1}H-NMR (700 MHz, δ, $CDCl_3$, 298 K): 1.65 (s, 3H), 1.86 (s, 3H), 2.98 (d, 1H, J = 16.3 Hz), 3.44 (s, 9H), 3.91 (dd, 1H, J_1 = 9.2 Hz, J_2 = 1.7 Hz), 4.46 (dd, 1H, J_1 =

---

[2] S. Kanemasa and K. Onimura, *Tetrahedron* 1992, **48**, 8631-8644.
[3] R. J. R. Lumby, P. M. Joensuu and H. W. Lam, *Tetrahedron* 2008, **64**, 7729-7740.

9.2 Hz, J_2 = 6.5 Hz), 5.83 (dd, 1H, J_1 = 6.5 Hz, J_2 = 1.7 Hz), 6.07 (d, 1H, J = 16.3 Hz), 7.28 - 7.51 (m, 5H) ppm; ^{13}C NMR (176 MHz, δ, $CDCl_3$, 298 K): 23.5, 25.5, 54.7, 60.1, 64.8, 71.8, 97.5, 126.8, 128.5, 129.5, 140.5, 161.0 ppm; IR (film): $\tilde{\nu}$ = 3011, 2987, 2937, 2882, 1654, 1434, 1412, 1378, 1351, 1237, 1204, 1133, 1064, 1048, 923, 896, 843, 703, 664, 604, 579, 563, 517, 501 cm^{-1}; HRMS (ESI): *m/z* calcd for $C_{16}H_{25}N_2O_2^+$: 277.1910 $[M]^+$; found: 277.1904.

3. Syntheses of Epoxides Using Amide 6C:

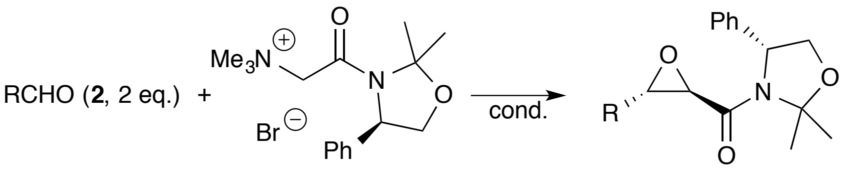

General Procedure: Ammonium salt **6C** was dissolved in the appropriate solvent (20 mL/mmol ammonium salt) and Cs_2CO_3 (20 eq.) was added to the reaction mixture. After 5 min the aldehyde (2 eq) was added and the suspension was stirred for the indicated time at the given temperature. The reaction was quenched with water and extracted with toluene. The organic phase was washed with brine and dried with anhydrous Na_2SO_4, filtrated and the solvent was removed under reduced pressure. The epoxide was purified by column chromatography (silica gel, heptanes:EtOAc = 7:3).

Cond. A: *i*-PrOH, 24 h, 25 °C

Cond. B: toluene, 24 h, 60 °C

Cond. C: toluene, 72 h, 25 °C

Cond. D: toluene, 24 h, 25 °C

***trans*-epoxide 17a.** Obtained in 78% (1 mmol scale) as a white solid after column

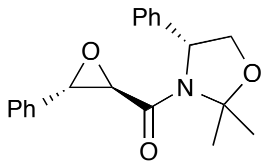

17a

chromatography (Cond. A). M.p.: 174 - 177 °C; $[\alpha]_D^{22}$ (c = 1.4, DCM) = -178; ^{1}H NMR (700 MHz, δ, $CDCl_3$, 298 K): 1.72 (s, 3H), 1.88 (s, 3H), 3.22 (d, 1H, J = 1.8 Hz), 3.68 (d, 1H, J = 1.8 Hz), 3.90 (dd, 1H, J_1 = 9.2 Hz, J_2 = 4.2 Hz), 4.41 (dd, 1H, J_1 = 9.2 Hz, J_2 = 6.6 Hz), 5.10 (dd, 1H, J_1 = 6.6 Hz, J_2 = 4.2 Hz), 6.78 (m, 2H), 7. 00 - 7.20 (m, 8H) ppm; ^{13}C NMR (176 MHz, δ, $CDCl_3$, 298 K): 24.3, 25.1, 58.4, 58.9, 61.5, 72.1, 97.3, 125.9, 126.1, 128.4,

128.5, 128.7, 129.4, 135.3, 140.6, 164.2 ppm; IR (film): $\bar{\nu}$ = 3032, 2989, 2933, 2873, 1658, 1458, 1437, 1387, 1363, 1252, 1205, 1081, 1066, 894, 849, 772, 749, 695, 660, 596, 552, 513 cm^{-1}; HRMS (ESI): *m/z* calcd for $C_{20}H_{21}NO_3$: 324.1594 [M + H]$^+$; found: 324.1595.

***trans*-epoxide 17b.** Obtained in 80% (0.28 mmol scale) as a white solid after column chromatography (Cond. A). M.p.: 139 - 141 °C; $[\alpha]_D^{22}$ (c = 0.6, DCM) = -161; ^{1}H NMR (300 MHz, δ, $CDCl_3$, 298 K): 1.71 (s, 3H), 1.88 (s, 3H), 2.28 (s, 3H), 3.22 (d, 1H, J = 1.5 Hz), 3.68 (d, 1H, J = 1.5 Hz), 3.90 (dd, 1H, J_1 = 9.0 Hz, J_2 = 4.0 Hz), 4.40 (dd, 1H, J_1 = 9.0 Hz, J_2 = 6.8 Hz), 5.11 (dd, 1H, J_1 = 6.8 Hz, J_2 =4.0 Hz), 6.67 (d, 2H, J = 8.1 Hz), 6.95 (m, 2H), 7.10 - 7.21 (m, 5H) ppm; ^{13}C NMR (75 MHz, δ, $CDCl_3$, 298 K): 21.5, 24.3, 25.2, 58.4, 58.6, 61.0, 72.3, 97.7, 125.9, 126.1, 128.3, 129.0, 129.4, 132.3, 138.5, 140.7, 164.5 ppm; IR (film): $\bar{\nu}$ = 2981, 2934, 2865, 2873, 1619, 1444, 1415, 1378, 1361, 1321, 1305, 1289, 1255, 1205, 1168, 1141, 1076, 1064, 1056, 891, 849, 830, 796, 772, 697, 683, 664, 603, 553, 522, 513, 492 cm^{-1}; HRMS (ESI): *m/z* calcd for $C_{21}H_{23}NO_3$: 338.1751 [M + H]$^+$; found: 338.1757.

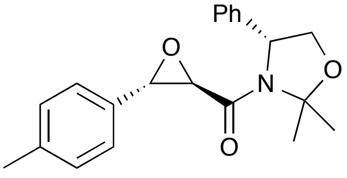

17b

***trans*-epoxide 17c.** Obtained in 79% (0.15 mmol scale) as a colourless residue after column chromatography (Cond. A). M.p.: 94 - 97 °C; $[\alpha]_D^{22}$ (c = 1.0, DCM) = -32; ^{1}H NMR (300 MHz, δ, $CDCl_3$, 298 K): 1.71 (s, 3H), 1.91 (s, 3H), 2.08 (s, 3H), 3.21 (d, 1H, J = 1.4 Hz), 3.95 (dd, 1H, J_1 = 9.0 Hz, J_2 = 3.3 Hz), 3.98 (d, 1H, J = 1.4 Hz), 4.43 (dd, 1H, J_1 = 9.0 Hz, J_2 = 6.2 Hz), 5.16 (dd, 1H, J_1 = 6.2 Hz, J_2 =3.3 Hz), 6.60 (d, 1H, J = 7.9 Hz), 6.97 - 7.00 (m, 2H), 7.06 - 7.25 (m, 6H) ppm; ^{13}C NMR (75 MHz, δ, $CDCl_3$, 298 K): 19.0, 24.0, 25.4, 56.7, 57.5, 61.4, 72.0, 97.3, 124.4, 126.0, 126.3, 128.3, 128.4, 129.5, 130.1, 133.7, 137.1, 141.2, 164.9 ppm; IR (film): $\bar{\nu}$ = 3026, 2936, 1646, 1436, 1389, 1376, 1364, 1316, 1239, 1064, 1043, 892, 843, 765, 706, 655, 609, 587, 564, 523 cm^{-1}; HRMS (ESI): *m/z* calcd for $C_{21}H_{23}NO_3$: 338.1751 [M + H]$^+$; found: 338.1756.

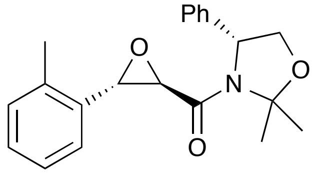

17c

***trans*-epoxide 17d.** Obtained in 75% (0.3 mmol scale) as a colourless residue after column

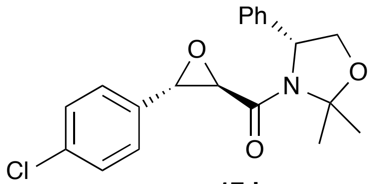

17d

chromatography (Cond. A). M.p.: 133 - 136 °C; $[\alpha]_D^{22}$ (c = 1.8, DCM) = -119; ^{1}H NMR (300 MHz, δ, $CDCl_3$, 298 K): 1.70 (s, 3H), 1.87 (s, 3H), 3.17 (d, 1H, J = 1.4 Hz), 3.62 (d, 1H, J = 1.4 Hz), 3.85 (dd, 1H, J_1 = 9.2 Hz, J_2 = 4.2 Hz), 4.40 (dd, 1H, J_1 = 9.2 Hz, J_2 = 6.8 Hz), 5.08 (dd, 1H, J_1 = 6.8 Hz, J_2 = 4.2 Hz), 6.70 (d, 2H, J = 8.3 Hz), 7.05 - 7.16 (m, 7H) ppm; ^{13}C NMR (75 MHz, δ, $CDCl_3$, 298 K): 24.4, 25.1, 57.6, 58.8, 62.3, 72.2, 97.3, 126.1, 127.2, 128.5, 128.6, 129.5, 133.9, 134.6, 140.6, 164.2 ppm; IR (film): $\tilde{\nu}$ = 2981, 2937, 2863, 2384, 2349, 2307, 1657, 1600, 1495, 1445, 1412, 1377, 1362, 1247, 1320, 1303, 1255, 1205, 1166, 1141, 1077, 1064, 1013, 891, 848, 833, 791, 770, 758, 731, 699, 665, 655, 553 cm^{-1}; HRMS (ESI): *m/z* calcd for $C_{20}H_{20}ClNO_3$: 358.1204 $[M + H]^+$; found: 358.1209.

***trans*-epoxide 17e.** Obtained in 85% (0.6 mmol scale) as a slightly yellowish solid after

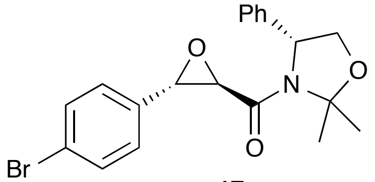

17e

column chromatography (Cond. A). M.p.: 258 - 260 °C; $[\alpha]_D^{22}$ (c = 0.5, DCM) = -75; ^{1}H NMR (300 MHz, δ, $CDCl_3$, 298 K): 1.70 (s, 3H), 1.88 (s, 3H), 3.17 (d, 1H, J = 1.3 Hz), 3.61 (d, 1H, J = 1.3 Hz), 3.88 (dd, 1H, J_1 = 9.1 Hz, J_2 = 4.2 Hz), 4.41 (dd, 1H, J_1 = 9.1 Hz, J_2 = 6.8 Hz), 5.08 (dd, 1H, J_1 = 6.8 Hz, J_2 = 4.2 Hz), 6.63 (d, 2H, J = 7.8 Hz), 7.05 - 7.31 (m, 7H) ppm; ^{13}C NMR (75 MHz, δ, $CDCl_3$, 298 K): 24.3, 25.1, 57.6, 58.8, 61.4, 72.2, 97.4, 122.6, 126.1, 127.5, 128.6, 129.5, 131.5, 134.4, 140.7, 164.2 ppm; IR (film): $\tilde{\nu}$ = 2982, 2934, 2862, 2655, 1594, 1489, 1444, 1408, 1377, 1360, 1319, 1253, 1205, 1166, 1139, 1066, 1010, 971, 935, 889, 845, 831, 804, 789, 768, 757, 723, 699, 664, 638, 562, 553 cm^{-1}; HRMS (ESI): *m/z* calcd for $C_{20}H_{20}BrNO_3$: 402.0699 $[M + H]^+$; found: 402.0702.

***trans*-epoxide 17f.** Obtained in 89% (0.25 mmol scale) as a white solid after column

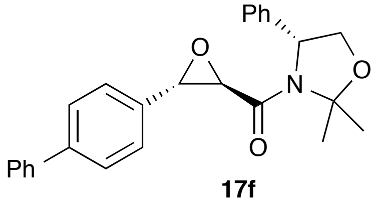

17f

chromatography (Cond. A). M.p.: 140 - 143 °C; $[\alpha]_D^{22}$ (c = 0.6, DCM) = -11; ^{1}H NMR (700 MHz, δ, $CDCl_3$, 298 K): 1.72 (s, 3H), 1.90 (s, 3H), 3.26 (d, 1H, J = 1.8 Hz), 3.72 (d, 1H, J = 1.8 Hz), 3.90 (dd, 1H, J_1 = 9.2 Hz, J_2 = 4.2 Hz), 4.42 (dd, 1H, J_1 = 9.2 Hz, J_2 = 6.9 Hz), 5.12 (dd, 1H, J_1 = 6.9 Hz, J_2 = 4.2 Hz), 6.84 - 6.68 (m, 2H), 7.05 - 7.82 (m, 12H) ppm; ^{13}C NMR (176 MHz, δ, $CDCl_3$, 298 K): 24.6, 25.3, 58.3, 58.9, 61.5, 72.2, 97.2, 126.2, 126.3, 127.1, 127.3, 127.8, 128.5, 129.2, 129.5, 134.8, 140.6, 140.9, 141.7, 164.5

ppm; IR (film): $\tilde{\nu}$ = 3029, 2970, 2928, 2876, 1656, 1442, 1411, 1377, 1362, 1324, 1253, 1201, 1136, 1066, 893, 851, 772, 759, 743, 696, 662, 563, 549, 515 cm^{-1}; HRMS (ESI): *m/z* calcd for $C_{26}H_{25}NO_3$: 400.1907 $[M + H]^+$; found: 400.1907.

***trans*-epoxide 17g.** Obtained in 73% (0.5 mmol scale) as a yellowish residue after column chromatography (Cond. A, compound **17g** partially decomposes during silica gel column chromatography).

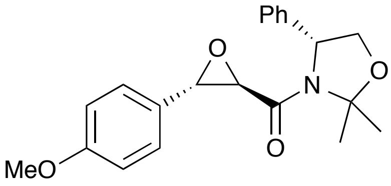

17g

$[\alpha]_D^{22}$ (c = 1.0, DCM) = -132; ^{1}H NMR (300 MHz, δ, $CDCl_3$, 298 K): 1.71 (s, 3H), 1.87 (s, 3H), 3.22 (d, 1H, J = 1.4 Hz), 3.62 (d, 1H, J = 1.4 Hz), 3.82 (s, 3H), 3.88 (dd, 1H, J_1 = 9.1 Hz, J_2 = 4.0 Hz), 4.39 (dd, 1H, J_1 = 9.1 Hz, J_2 = 6.8 Hz), 5.10 (dd, 1H, J_1 = 6.8 Hz, J_2 = 4.0 Hz), 6.62 - 6.74 (m, 4H), 7.02 - 7.20 (m, 5H) ppm; ^{13}C NMR (75 MHz, δ, $CDCl_3$, 298 K): 24.3, 25.3, 56.0, 58.0, 58.2, 61.2, 71.6, 97.2, 114.1, 126.1, 127.1, 127.2, 128.4, 129.6, 140.7, 160.1, 164.8 ppm; IR (film): $\tilde{\nu}$ = 2976, 2935, 2862, 2838, 1655, 1612, 1515, 1442, 1420, 1386, 1377, 1362, 1322, 1247, 1205, 1175, 1138, 1079, 1064, 1032, 892, 848, 833, 800, 772, 754, 698, 680, 661, 570, 552, 514 cm^{-1}; HRMS (ESI): *m/z* calcd for $C_{21}H_{23}NO_4$: 354.1700 $[M + H]^+$; found: 354.1707.

***trans*-epoxide 17h.** Obtained in 60% (0.4 mmol scale) crude yield (Cond. B, compound **17h** fully decomposes during silica gel column chromatography).

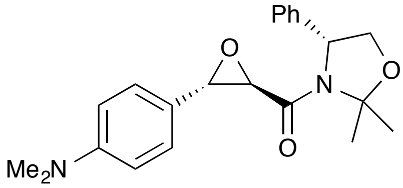

17h

^{1}H NMR (700 MHz, δ, $CDCl_3$, 298 K): 1.69 (s, 3H), 1.87 (s, 3H), 2.89 (s, 6H), 3.26 (d, 1H, J = 1.9 Hz), 3.63 (d, 1H, J = 1.9 Hz), 3.90 (dd, 1H, J_1 = 9.0 Hz, J_2 = 3.8 Hz), 4.40 (dd, 1H, J_1 = 9.0 Hz, J_2 = 6.9 Hz), 5.10 (dd, 1H, J_1 = 6.9 Hz, J_2 = 3.8 Hz), 6.48 (m, 2H), 6.65 (m, 2H), 7.05 - 7.36 (m, 5H) ppm; ^{13}C NMR (75 MHz, δ, $CDCl_3$, 298 K): 24.3, 24.9, 40.8, 58.4, 58.7, 61.2, 72.3, 97.3, 112.4, 125.5, 126.2, 126.4, 127.8, 129.4, 140.8, 150.1, 165.4 ppm; HRMS (ESI): *m/z* calcd for $C_{22}H_{26}N_2O_3$: 367.2016 $[M + H]^+$; found: 367.2019.

***trans*-epoxide 17i.** Obtained in 62% (0.45 mmol scale) as a slightly yellowish residue after

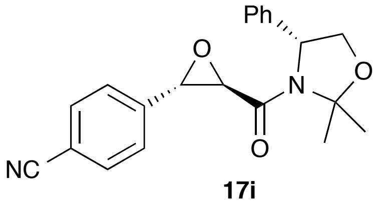

17i

column chromatography (Cond. C). M.p.: 85 - 88 °C; $[\alpha]_D^{22}$ (c = 0.5, DCM) = -113; ^{1}H NMR (300 MHz, δ, $CDCl_3$, 298 K): 1.71 (s, 3H), 1.88 (s, 3H), 3.16 (d, 1H, *J* = 1.7 Hz), 3.69 (d, 1H, *J* = 1.7 Hz), 3.87 (dd, 1H, J_1 = 9.3 Hz, J_2 = 4.4 Hz), 4.42 (dd, 1H, J_1 = 9.3 Hz, J_2 = 6.8 Hz), 5.07 (dd, 1H, J_1 = 6.8 Hz, J_2 = 4.4 Hz), 6.97 (m, 2H), 7.11 (m, 5H), 7.44 (m, 2H) ppm; ^{13}C NMR (75 MHz, δ, $CDCl_3$, 298 K): 24.4, 25.2, 57.5, 59.0, 61.5, 72.2, 97.5, 112.7, 119.0, 126.1, 126.4, 128.6, 129.5, 132.2, 140.6, 140.8, 165.6 ppm; IR (film): $\tilde{\nu}$ = 3093, 3046, 2857, 2744, 2229, 1702, 1658, 1607, 1571, 1416, 1383, 1361, 1309, 1295, 1251, 1201, 1171, 1139, 1071, 1016, 893, 828, 772, 736, 705, 643, 562, 545 cm^{-1}; HRMS (ESI): *m/z* calcd for $C_{21}H_{20}N_2O_3$: 349.1547 $[M + H]^+$; found: 349.1552.

***trans*-epoxide 17j.** Obtained in 68% (0.5 mmol scale) as a yellow solid after column

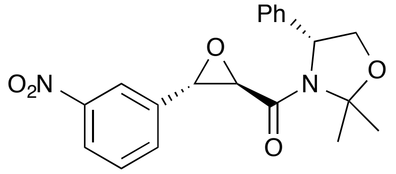

17j

chromatography (Cond. C). M.p.: 117 - 120 °C; $[\alpha]_D^{22}$ (c = 0.4, DCM) = -183; ^{1}H NMR (300 MHz, δ, $CDCl_3$, 298 K): 1.72 (s, 3H), 1.89 (s, 3H), 3.20 (d, 1H, *J* = 1.8 Hz), 3.72 (d, 1H, *J* = 1.8 Hz), 3.87 (dd, 1H, J_1 = 9.2 Hz, J_2 = 4.6 Hz), 4.42 (dd, 1H, J_1 = 9.2 Hz, J_2 = 6.8 Hz), 5.07 (dd, 1H, J_1 = 6.8 Hz, J_2 = 4.6 Hz), 6.96 - 7.17 (m, 6H), 7.35 (m, 1H), 7.58 (m, 1H), 8.10 (m, 1H) ppm; ^{13}C NMR (75 MHz, δ, $CDCl_3$, 298 K): 24.5, 25.1, 57.1, 59.1, 61.6, 72.3, 97.5, 121.0, 123.6, 126.1, 128.5, 129.5, 129.6, 131.5, 137.7, 140.2, 148.3, 163.5 ppm; IR (film): $\tilde{\nu}$ = 2986, 2934, 2877, 1659, 1651, 1528, 1454, 1433, 1378, 1349, 1305, 1252, 1203, 1167, 1138, 1064, 889, 847, 802, 775, 735, 700, 677, 610, 565, 552, 515 cm^{-1}; HRMS (ESI): *m/z* calcd for $C_{20}H_{20}N_2O_5$: 369.1445 $[M + H]^+$; found: 369.1449.

***trans*-epoxide 17k.** Obtained in 42% (0.4 mmol scale) as a colourless hygroscopic residue

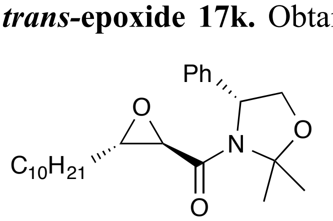

17k

after column chromatography (Cond. D). $[\alpha]_D^{22}$ (c = 0.5, DCM) = -68; ^{1}H NMR (300 MHz, δ, $CDCl_3$, 298 K): 0.81 - 1.36 (m, 21H), 1.67 (s, 3H), 1.85 (s, 3H), 2.83 (dt, 1H, J_1 = 5.6 Hz, J_2 = 2.1 Hz), 2.93 (d, 1H, *J* = 2.1 Hz), 3.91 (dd, 1H, J_1 = 9.1 Hz, J_2 = 3.9 Hz), 4.42 (dd, 1H, J_1 = 9.1 Hz, J_2 = 6.8 Hz), 5.15 (dd, 1H, J_1 = 6.8 Hz, J_2 = 3.9 Hz), 7.24 - 7.32 (m, 5H) ppm; ^{13}C NMR (75 MHz, δ, $CDCl_3$, 298 K): 14.5, 23.0, 24.1, 25.2, 25.5, 29.5, 29.6, 29.8 (2x), 29.9, 31.1, 32.2, 54.5, 59.2, 61.4, 71.8, 97.5, 126.3, 128.5, 129.6, 141.5, 165.8

ppm; IR (film): $\tilde{\nu}$ = 2923, 2853, 1657, 1450, 1399, 1376, 1363, 1253, 1204, 1169, 1140, 1067, 904, 848, 758, 700, 664, 518 cm^{-1}; HRMS (ESI): *m/z* calcd for $C_{24}H_{37}NO_3$: 388.2846 $[M + H]^+$; found: 388.2847.

***trans*-epoxide 17l.** Obtained in 39% (0.5 mmol scale) as a colourless residue after column

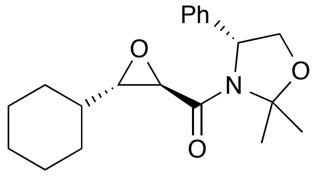

17l

chromatography (Cond. D). M.p.: 63 - 65 °C; $[\alpha]_D^{22}$ (c = 0.6, DCM) = -76; ^{1}H NMR (300 MHz, δ, $CDCl_3$, 298 K): 0.46 - 1.63 (m, 11H), 1.66 (s, 3H), 1.86 (s, 3H), 2.76 (dd, 1H, J_1 = 5.5 Hz, J_2 = 1.9 Hz), 3.04 (d, 1H, J = 1.9 Hz), 3.91 (dd, 1H, J_1 = 9.1 Hz, J_2 = 3.3 Hz), 4.42 (dd, 1H, J_1 = 9.1 Hz, J_2 = 6.4 Hz), 5.16 (dd, 1H, J_1 = 6.4 Hz, J_2 = 3.3 Hz), 7.25 - 7.42 (m, 5H) ppm; ^{13}C NMR (75 MHz, δ, $CDCl_3$, 298 K): 24.0, 25.2, 25.7, 25.8, 26.3, 28.3, 28.8, 38.9, 52.3, 61.6, 63.3, 71.9, 97.3, 126.3, 128.5, 129.4, 141.9, 166.1 ppm; IR (film): $\tilde{\nu}$ = 2984, 2926, 2850, 1644, 1444, 1395, 1374, 1362, 1339, 1314, 1283, 1247, 1235, 1205, 1164, 1132, 1064, 974, 900, 885, 844, 772, 703, 660, 578, 564, 514 cm^{-1}; HRMS (ESI): *m/z* calcd for $C_{20}H_{27}NO_3$: 320.2064 $[M + H]^+$; found: 320.2070.

4. Reduction of Epoxide 17a:

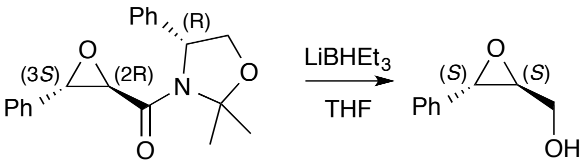

In analogy to literature,[4] **17a** (130 mg, 0.4 mmol) was dissolved in 5 mL THF. Then $LiBHEt_3$ (1.0 mL, 1 mmol, 1 M THF, 2.5 eq.) was added to the mixture and stirred for 35 min. The reaction was quenched with aqueous NH_4Cl solution followed by the extraction with Et_2O and brine. The organic phase was dried with anhydrous Na_2SO_4, filtrated, and the solvent removed under reduced pressure. Epoxyalcohol **18** (32 mg, 0.21 mmol) was obtained after column chromatography (silica gel, heptanes: EtOAc = 8:2 → 1:1) as a colourless oil in 53% non-optimized yield. Analytical data are in accordance to literature.[5] $[\alpha]_D^{20}$ (c = 1.0, DCM) = -57; ^{1}H-NMR (300 MHz, δ, $CDCl_3$, 298 K): 1.80 (br, 1H), 3.10 (m, 1H), 3.76 (dd, 1H, J_1 =

[4] F. Sarabia, C. Vivar-Garcia, M. Garcia-Castro and J. Martin-Ortiz, *J. Org. Chem.* 2011, **76**, 3139-3150.
[5] a) S. K., Cherian and P. Kumar, *Tetrahedron: Asymmetry*. 2007, **18**, 982-987; b) F. Sarabia, S. Chammaa, M. Garica-Castro and F. Martin-Galvez, *Chem. Commun.*, 2009, 5763-5765; c) P. G. Gordillo, D. M. Aparicio, M. Flores, A. Mendoza, L. Orea, J. R. Juarez, G. Huelgas, D. Gnecco and J. L. Teran, *Eur. J. Org. Chem.*, 2013, 5561-5565.

12.7 Hz, J_2 = 3.9 Hz), 3.93 (d, 1H, J = 1.8 Hz), 3.98 (dd, 1H, J_1 = 12.7 Hz, J_2 = 2.2 Hz), 7.20 - 7.42 (m, 5H) ppm.

5. Syntheses of Aziridines Using Amide 6C:

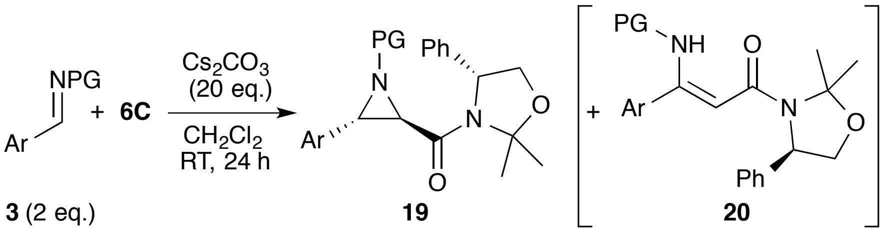

3 (2 eq.) 19 20

General procedure for the preparation of aziridines: A mixture of **6C** (0.4 mmol), aldimine **3** (2 eq.), and 20 equivalents of solid Cs_2CO_3 in CH_2Cl_2 (8 mL) was vigorously stirred for 24 h at room temperature. CH_2Cl_2 and brine were added and the phases separated. The aqueous layer was extracted twice with CH_2Cl_2, the combined organic layers were extracted with brine and the aqueous layer was re-extracted twice with CH_2Cl_2. The combined organic layers were dried over Na_2SO_4, filtrated, evaporated, and dried *in vacuo*. Column chromatography (silica gel, heptanes/EtOAc = 20:1 – 2:1) gave the aziridines 1**9** in the reported yields. In most cases the minor *cis*-isomers and the olefins **20** could not be obtained in pure form.

***trans*-N-tosyl aziridine 19a.** Obtained in 39% as a colourless oil after column

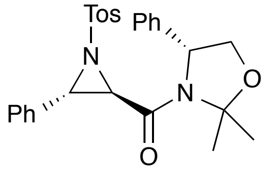

19a

chromatography (crude NMR yield 70%). $[\alpha]_D^{23}$ (c = 1.2, DCM) = -78; ^{1}H NMR (300 MHz, δ, $CDCl_3$, 298 K): 1.76 (s, 3H), 1.95 (s, 3H), 2.42 (s, 3H), 3.21 (d, 1H, J = 4.1 Hz), 4.03 (dd, 1H, J = 8.9, 1.6 Hz), 4.44 (d, 1H, J = 4.1 Hz), 4.53 (dd, 1H, J = 8.9, 6.3 Hz), 5.56 (dd, 1H, J = 6.3, 1.6 Hz), 6.85 – 6.93 (m, 2H), 7.21 – 7.48 (m, 10H), 7.77 – 7.85 (m, 2H) ppm; ^{13}C NMR (75 MHz, δ, $CDCl_3$, 298 K): 21.6, 22.3, 25.4, 47.1, 49.5, 61.2, 71.5, 96.9, 126.2, 126.9, 127.6, 128.1, 128.3, 128.4, 129.2, 129.7, 132.9, 136.5, 141.3, 144.6, 161.4 ppm; IR (film): $\tilde{\nu}$ = 2970, 1662, 1432, 1378, 1329, 1238, 1204, 1159, 1085, 1063, 929, 812, 756, 695, 579 cm^{-1}; HRMS (ESI): *m/z* calcd for $C_{27}H_{28}N_2O_4S$: 477.1843 $[M + H]^+$; found: 477.1834.

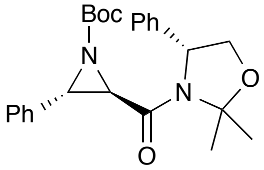

19b

***trans*-N-Boc aziridine 19b.** Obtained in 62% as a colourless residue. M.p.: 136 - 139 °C; $[\alpha]_D^{23}$ (c = 1.6, DCM) = -110; ^{1}H NMR (500 MHz, δ, $CDCl_3$, 298 K): 1.48 (s, 9H), 1.73 (s, 3H), 1.89 (s, 3H), 2.87 (d, 1H, *J* = 2.5 Hz), 3.69 (d, 1H, *J* = 2.5 Hz), 3.96 (dd, 1H, *J* = 9.1, 3.5 Hz), 4.47 (dd, 1H, *J* = 9.1, 6.8 Hz), 5.28 (dd, 1H, *J* = 6.8, 3.5 Hz), 6.88 (d, 2H, *J* = 6.9 Hz), 7.19 – 7.28 (m, 8H) ppm; ^{13}C NMR (125 MHz, δ, $CDCl_3$, 298 K): 23.8, 25.0, 28.0, 44.5, 44.8, 61.5, 71.6, 81.7, 96.8, 125.7, 126.5, 127.7, 128.0, 128.1, 129.1, 135.3, 140.9, 159.2, 163.7 ppm; IR (film): $\bar{\nu}$ = 3009, 2984, 2935, 2868, 1715, 1652, 1433, 1395, 1364, 1333, 1253, 1223, 1204, 1074, 1053, 819, 755, 746, 705, 691, 635 cm^{-1}; HRMS (ESI): *m/z* calcd for $C_{25}H_{30}N_2O_4$: 423.2278 $[M + H]^+$; found: 423.2287.

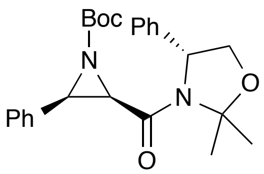

cis-**19b**

***cis*-N-Boc aziridine 19b.** Obtained in trace amounts and lower purity during the isolation of *trans*-**19b**. ^{1}H NMR (500 MHz, δ, $CDCl_3$, 298 K): 1.41 (s, 9H), 1.46 (s, 3H), 1.54 (s, 3H), 3.27 (d, 1H, *J* = 6.8 Hz), 3.33 (d, 1H, *J* = 6.8 Hz), 3.87 (dd, 1H, *J* = 8.9, 2.4 Hz), 4.29 (dd, 1H, *J* = 8.9, 6.5 Hz), 5.29 (dd, 1H, *J* = 6.5, 2.4 Hz), 7.02 – 7.09 (m, 2H), 7.18 – 7.25 (m, 4H), 7.34 – 7.40 (m, 4H) ppm; ^{13}C NMR (125 MHz, δ, $CDCl_3$, 298 K): 22.9, 25.1, 27.8, 44.5, 45.6, 60.8, 71.6, 82.1, 96.8, 126.4, 127.6, 127.8, 127.9, 128.0, 129.0, 132.9, 141.7, 161.0, 162.1 ppm; HRMS (ESI): *m/z* calcd for $C_{25}H_{30}N_2O_4$: 423.2278 $[M + H]^+$; found: 423.2282.

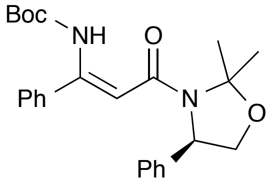

20b

Olefine 20b. Obtained in minor amounts during the isolation of *trans*-**19b**. ^{1}H NMR (700 MHz, δ, $CDCl_3$, 298 K): 1.34 (s, 9H), 1.72 (s, 3H), 1.91 (s, 3H), 3.91 (dd, 1H, *J* = 8.9, 3.2 Hz), 4.37 (dd, 1H, *J* = 8.9, 6.7 Hz), 4.86 (s, 1H), 4.91 (dd, 1H, *J* = 6.7, 3.2 Hz), 6.97 (d, 2H, *J* = 7.3 Hz), 7.21 (t, 2H, *J* = 7.8 Hz), 7.27 - 7.34 (m, 4H), 7.36 – 7.41 (m, 2H), 10.84 (s, 1H) ppm; ^{13}C NMR (176 MHz, δ, $CDCl_3$, 298 K): 23.9, 25.8, 28.2, 61.9, 71.6, 80.9, 96.4, 101.6, 126.3, 127.2, 127.8, 128.1, 129.1, 129.2, 137.3, 141.4, 152.2, 152.9, 166.1 ppm; HRMS (ESI): *m/z* calcd for $C_{25}H_{30}N_2O_4$: 423.2278 $[M + H]^+$; found: 423.2284.

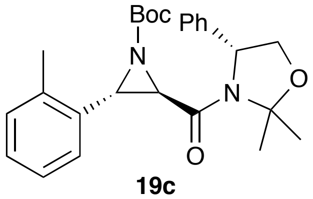

19c

***trans*-N-Boc aziridine 19c.** Obtained in 63% as a mixture of *trans* and *cis*-aziridine (7.5:1). $[\alpha]_D^{23}$ (c = 2.6, DCM) = -99; ^{1}H NMR (500 MHz, δ, $CDCl_3$, 298 K): *major-trans*: 1.46 (s, 9H), 1.74 (s, 3H), 1.92 (s, 3H), 2.21 (s, 3H), 2.94 (d, 1H, *J* = 2.2 Hz), 3.86 (d, 1H, *J* = 2.2 Hz), 4.00 (dd, 1H, *J* = 8.9, 2.1 Hz), 4.49 (dd, 1H, *J* = 8.9, 6.8 Hz), 5.31 (dd, 1H,

J = 6.8, 2.1 Hz), 6.53 (d, 1H, J = 7.5 Hz), 6.90 – 6.95 (m, 1H), 7.00 – 7.10 (m, 1H), 7.12 – 7.40 (m, 6H) ppm; ^{13}C NMR (125 MHz, δ, $CDCl_3$, 298 K): 18.9, 23.5, 25.2, 27.9, 43.2, 43.4, 61.4, 71.5, 81.7, 96.8, 125.6, 125.8, 125.9, 127.7, 128.0, 129.2, 129.7, 133.4, 137.4, 141.5, 159.5, 163.9 ppm; IR (film): $\bar{\nu}$ = 2979, 2934, 1725, 1652, 1429, 1390, 1365, 1328, 1305, 1284, 1249, 1205, 1151, 1083, 1069, 1050, 814, 765, 753, 703 cm^{-1}; HRMS (ESI): *m/z* calcd for $C_{26}H_{32}N_2O_4$: 437.2435 [M + H]$^+$; found: 437.2433.

***trans*-N-Boc aziridine 19d.** Obtained in 57% after neutral alumina column chromatography (**19d** is relatively unstable and quickly decomposed at higher temperature or under more acidic conditions). $[\alpha]_D^{23}$ (c = 1.5, DCM) = -96; ^{1}H NMR (300 MHz, δ, $CDCl_3$, 298 K): 1.37 (s, 9H), 1.64 (s, 3H), 1.79 (s, 3H), 2.76 (d, 1H, J = 2.6 Hz), 3.56 (d, 1H, J = 2.6 Hz), 3.67 (s, 3H), 3.87 (dd, 1H, J = 8.9, 3.4 Hz), 4.37 (dd, 1H, J = 8.9, 6.7 Hz), 5.19 (dd, 1H, J = 6.7, 3.4 Hz), 6.58 (d, 2H, J = 8.6 Hz), 6.69 (d, 2H, J = 8.6 Hz), 7.05 – 7.18 (m, 5H) ppm; ^{13}C NMR (75 MHz, δ, $CDCl_3$, 298 K): 23.8, 25.0, 27.9, 44.2, 44.6, 52.2, 61.5, 81.6, 96.8, 113.5, 125.8, 127.4, 127.7, 128.0, 129.1, 141.0, 159.2, 159.3, 163.9 ppm; IR (film): $\bar{\nu}$ = 2933, 1713, 1651, 1518, 1420, 1398, 1365, 1289, 1155, 1080, 1064, 1033, 815, 752, 703, 619 cm^{-1}; HRMS (ESI): *m/z* calcd for $C_{26}H_{32}N_2O_5$: 453.2384 [M + H]$^+$; found: 453.2383.

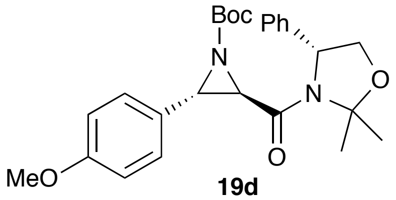

19d

***trans*-N-Boc aziridine 19e.** Obtained in 58% as a colourless hygroscopic residue (containing some co-eluting starting imine-based impurities). $[\alpha]_D^{23}$ (c = 0.2, DCM) = -93; ^{1}H NMR (300 MHz, δ, $CDCl_3$, 298 K): 1.49 (s, 9H), 1.75 (s, 3H), 1.91 (s, 3H), 2.97 (d, 1H, J = 2.6 Hz), 3.84 (d, 1H, J = 2.6 Hz), 3.96 (dd, 1H, J = 9.0, 3.6 Hz), 4.47 (dd, 1H, J = 9.0, 6.8 Hz), 5.29 (dd, 1H, J = 6.8, 3.6 Hz), 6.96 – 7.03 (m, 2H), 7.11 – 7.25 (m, 3H), 7.32 (s, 1H), 7.41 – 7.79 (m, 6H) ppm; ^{13}C NMR (75 MHz, δ, $CDCl_3$, 298 K): 23.9, 25.0, 28.0, 44.7, 44.9, 61.5, 71.6, 81.8, 96.8, 124.0, 125.7, 125.8, 125.9, 126.1, 127.6, 127.7, 127.8, 128.0, 128.1, 129.1, 132.9, 133.0, 140.9, 159.3, 164.0 ppm; IR (film): $\bar{\nu}$ = 2970, 2929, 1725, 1651, 1435, 1324, 1249, 1148, 1050, 815, 753, 702 cm^{-1}; HRMS (ESI): *m/z* calcd for $C_{29}H_{32}N_2O_4$: 473.2435 [M + H]$^+$; found: 473.2429.

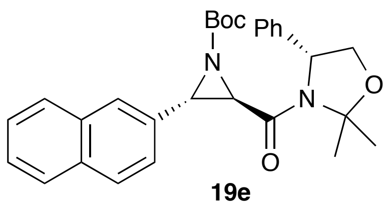

19e

***trans*-N-Boc aziridine 19f.** Obtained in 32% as a colourless solid (Olefine **20f** could not be isolated in pure form due to co-elution of different impurities and starting imine decomposition products). M.p.: 149 - 151 °C; $[\alpha]_D^{23}$ (c = 1.1, DCM) = -99; ^{1}H NMR (700 MHz, δ, $CDCl_3$, 298 K): 1.46 (s, 9H), 1.70 (s, 3H), 1.85 (s, 3H), 2.75 (d, 1H, *J* = 2.5 Hz), 3.56 (d, 1H, *J* = 2.5 Hz), 3.92 (dd, 1H, *J* = 9.1, 3.8 Hz), 4.43 (dd, 1H, *J* = 9.1, 6.8 Hz), 5.21 (dd, 1H, *J* = 6.8, 3.8 Hz), 6.70 (d, 2H, *J* = 8.3 Hz), 7.10 – 7.14 (m, 1H), 7.18 – 7.23 (m, 6H) ppm; ^{13}C NMR (176 MHz, δ, $CDCl_3$, 298 K): 24.1, 25.0, 28.1, 43.9, 45.3, 61.8, 71.8, 82.1, 97.0, 121.8, 125.9, 127.8, 128.2, 128.3, 129.4, 131.3, 131.8, 134.8, 140.9, 159.2, 163.5 ppm; IR (film): $\tilde{\nu}$ = 2981, 2970, 1717, 1654, 1438, 1412, 1387, 1365, 1295, 1250, 1155, 1073, 1012, 873, 850, 806, 729, 705, 656 cm^{-1}; HRMS (ESI): *m/z* calcd for $C_{25}H_{29}BrN_2O_4$: 501.1383 $[M + H]^+$; found: 501.1376.

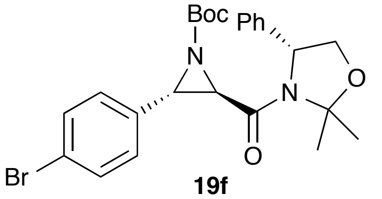

19f

Olefine 20g. Obtained in around 50% containing some co-eluting impurities that could not be separated. ^{1}H NMR (300 MHz, δ, $CDCl_3$, 298 K): 1.38 (s, 9H), 1.76 (s, 3H), 1.94 (s, 3H), 3.96 (dd, 1H, *J* = 9.0, 3.7 Hz), 4.42 (dd, 1H, *J* = 9.0, 6.7 Hz), 4.93 (s, 1H), 4.94 (dd, 1H, *J* = 6.7, 3.7 Hz), 7.30 – 7.48 (m, 7H), 7.75 – 7.82 (m, 1H), 8.13 – 8.21 (m, 1H), 10.92 (s, 1H) ppm; ^{13}C NMR (75 MHz, δ, $CDCl_3$, 298 K): 23.9, 25.4, 28.0, 61.9, 71.4, 81.5, 96.3, 102.9, 121.9, 123.7, 126.1, 128.3, 128.5, 129.3, 133.1, 138.7, 140.8, 147.8, 149.8, 152.0, 165.3 ppm; IR (film): $\tilde{\nu}$ = 2979, 2934, 2874, 1731, 1614, 1529, 1487, 1390, 1348, 1247, 1224, 1203, 1147, 1069, 1049, 836, 806, 735, 700, 666 cm^{-1}; HRMS (ESI): *m/z* calcd for $C_{25}H_{29}N_3O_6$: 468.2129 $[M + H]^+$; found: 468.2124.

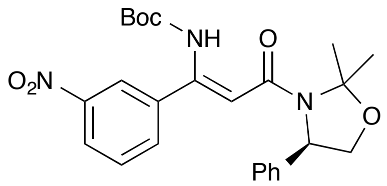

20g

6. DFT Calculations:

Energies and geometries

All geometries (cartesian coordinates in Å) were obtained after geometry optimization at the B3LYP-d3/6-31G*(CH_2Cl_2) level of theory. Energies (in a.u.) were obtained after single point calculations at the indicated level of theory.

Frequency calculations have been carried out at the B3LYP/6-31G*(CH_2Cl_2) level of theory in order to check the correct nature of the point on the potential energy surface and to obtain the thermal and entropic contributions to free energy (at 298.15 K) and zero-point energies.

(Z)-Ylide^{Re}

E(B3LYP-d3/6-31G*(CH_2Cl_2)) = -884.058884

E(B3LYP-d3/6-311+G**(CH_2Cl_2)) = -884.304324

G_{tot}(B3LYP-d3/6-31G*(CH_2Cl_2)) = -883.721283

C	2.40502	7.82372	13.76974
H	1.77337	7.50810	12.94133
O	-0.41306	6.11546	18.13832
N	1.85360	6.11952	17.69753
C	3.29982	8.88515	13.60265
C	2.31899	7.16503	14.99629
H	1.62297	6.34286	15.13577
C	3.12165	7.55918	16.07391
C	3.10860	6.79372	17.38563
H	3.42559	7.46666	18.20151
C	4.01016	8.62623	15.90261
H	4.63657	8.94224	16.73615
C	4.10282	9.28486	14.67336
H	4.80204	10.10947	14.5527
C	2.13022	4.80624	18.34157
O	3.57135	4.71168	18.33590
C	4.06211	5.58796	17.33152
C	1.53025	3.66774	17.51384
H	1.75470	2.69646	17.97142
H	1.93980	3.68652	16.49774
H	0.44830	3.80875	17.45726
C	1.71126	4.74211	19.80941
H	2.14404	5.58989	20.35099
H	2.08278	3.81157	20.25348
H	0.62468	4.78087	19.89369
C	0.72954	8.20050	18.08835
C	0.64512	6.81169	17.98597
N	-0.52235	9.01278	18.13940
H	1.57756	8.77940	17.76344
H	-1.50090	7.65998	19.42303
H	-0.64718	8.99605	20.25204
H	-2.20873	9.31975	19.42245
C	-1.28847	8.72784	19.41109
C	-1.41970	8.74133	16.95151
H	-1.66189	7.67928	16.96874
H	-2.32276	9.35494	17.02399
H	-0.85876	8.98831	16.04957
C	-0.14370	10.46694	18.13012
H	-1.04620	11.07501	18.22422
H	0.53232	10.65337	18.96785
H	0.36108	10.69252	17.18832

H	5.10019	5.84597	17.56208
H	4.02331	5.12212	16.33485
H	3.36953	9.39659	12.64492

(*Z*)-Ylide^{Si}

E(B3LYP-d3/6-31G*(CH_2Cl_2)) = -884.054861
E(B3LYP-d3/6-311+G**(CH_2Cl_2)) = -884.301148
G_{tot}(B3LYP-d3/6-31G*(CH_2Cl_2)) = -883.718214

C	2.98382	7.91794	13.74858
H	2.48525	7.63943	12.82166
O	0.86283	7.93826	18.69050
N	1.77007	6.13769	17.45347
C	3.91177	8.96438	13.75665
C	2.69371	7.22756	14.92688
H	1.96831	6.41895	14.93450
C	3.32342	7.57183	16.13001
C	3.08835	6.76633	17.39455
H	3.26797	7.40432	18.27259
C	4.24422	8.62699	16.13156
H	4.73410	8.90797	17.06300
C	4.54123	9.31769	14.95335
H	5.26249	10.13278	14.96970
C	1.89965	4.81895	18.13812
O	3.31732	4.66306	18.37028
C	3.99437	5.52938	17.46639
C	1.41376	3.69770	17.21197
H	1.55438	2.71609	17.68087
H	1.96816	3.72439	16.26759
H	0.35013	3.83586	16.99017
C	1.22322	4.75415	19.50683
H	1.54966	5.59520	20.12381
H	1.48930	3.81403	20.00194
H	0.13641	4.79977	19.39391
C	-0.50314	6.68263	17.19292
C	0.67010	7.01365	17.84312
N	-1.76903	7.42368	17.46759
H	-0.55218	6.01614	16.34698
H	-0.79450	9.27951	17.69117
H	-1.39663	8.94434	16.03968
H	-2.55628	9.41523	17.33126
C	-1.62440	8.88643	17.10507
C	-2.14648	7.31487	18.92705
H	-1.30788	7.70039	19.50555
H	-3.05542	7.89522	19.11330
H	-2.31189	6.25957	19.15086
C	-2.87134	6.82367	16.64149
H	-3.80366	7.35496	16.84943
H	-2.61256	6.92051	15.58388
H	-2.96835	5.76756	16.90337
H	4.99232	5.74015	17.86209
H	4.09551	5.07033	16.47035
H	4.14052	9.50162	12.83822

(*E*)-Ylide^{Re}

E(B3LYP-d3/6-31G*(CH_2Cl_2)) = -884.036428
E(B3LYP-d3/6-311+G**(CH_2Cl_2)) = -884.282907
G_{tot}(B3LYP-d3/6-31G*(CH_2Cl_2)) = -883.695308

C	4.84926	6.66759	14.07569
H	5.56417	5.96809	13.64578
O	0.97095	7.10096	19.43915
N	1.76417	6.15252	17.40489

C	4.42430	7.77579	13.33568
C	4.36726	6.45441	15.36882
H	4.71560	5.59782	15.93769
C	3.44510	7.34134	15.94790
C	2.88779	7.14400	17.35050
H	2.50738	8.10118	17.71160
C	3.04295	8.45861	15.20202
H	2.35103	9.17412	15.64419
C	3.52074	8.67485	13.90596
H	3.19364	9.54988	13.34634
C	2.38640	4.84789	17.82410
O	3.76675	5.15035	18.16162
C	3.88474	6.55887	18.35513
C	2.45850	3.85101	16.65849
H	3.20282	3.08052	16.88753
H	2.74739	4.35597	15.73327
H	1.49684	3.35160	16.50474
C	1.71272	4.19597	19.03491
H	1.78996	4.84073	19.90986
H	2.20511	3.23826	19.23718
H	0.65363	4.00820	18.82975
C	-0.61105	6.67514	17.78958
C	0.68445	6.67250	18.28363
H	3.58829	6.84301	19.37231
H	4.92740	6.83943	18.17369
H	4.80185	7.94078	12.32827
H	-1.38896	7.10637	18.40433
N	-1.07082	6.45793	16.38033
C	-0.44519	7.45265	15.42740
H	0.62979	7.28103	15.43251
H	-0.85301	7.30639	14.42223
H	-0.67186	8.45623	15.79030
C	-0.78164	5.06888	15.87275
H	0.29667	4.95853	15.84012
H	-1.22001	4.35196	16.56929
H	-1.21174	4.95072	14.87379
C	-2.56578	6.65471	16.35152
H	-2.92292	6.49429	15.33199
H	-3.02302	5.93503	17.03268
H	-2.79188	7.67352	16.67235

(*E*)-Ylide^{Si}
E(B3LYP-d3/6-31G*(CH_2Cl_2)) = -884.033835
E(B3LYP-d3/6-311+G**(CH_2Cl_2)) = -884.282209
G_{tot}(B3LYP-d3/6-31G*(CH_2Cl_2)) = -883.695300

C	2.00779	8.24398	14.44598
H	1.15368	8.21149	13.77245
O	-0.24058	5.90651	17.69680
N	2.04936	5.81307	18.09589
C	3.06408	9.12294	14.19204
C	2.03621	7.40440	15.56257
H	1.20408	6.73627	15.76352
C	3.12611	7.43633	16.44126
C	3.26082	6.48244	17.61811
H	3.75595	7.02761	18.43455
C	4.17575	8.33200	16.18888
H	5.02444	8.37117	16.87186
C	4.15049	9.16708	15.07027
H	4.97617	9.85147	14.88533
C	2.16790	4.35342	17.87396
O	3.59368	4.15734	17.93009

C	4.17183	5.27039	17.26040
C	1.63973	3.86791	16.51441
H	1.87191	2.80393	16.38629
H	2.09686	4.42646	15.69180
H	0.56239	4.02816	16.47012
C	1.55368	3.56778	19.02588
H	2.00447	3.88574	19.97098
H	1.72930	2.49512	18.88810
H	0.47665	3.75107	19.05703
C	0.59717	7.56206	19.07324
C	0.76348	6.42561	18.28112
H	5.19871	5.39561	17.61596
H	4.19798	5.11680	16.17298
H	3.03939	9.77265	13.31972
H	-0.38134	8.02096	19.06624
N	1.47926	8.16807	20.11931
C	2.38507	9.24684	19.55858
H	3.03250	8.81079	18.80395
H	1.75058	10.00233	19.09247
H	2.98202	9.69031	20.36244
C	2.28991	7.14082	20.87289
H	2.88215	6.56201	20.17116
H	2.93011	7.65244	21.59643
H	1.59080	6.47382	21.37952
C	0.57878	8.83944	21.13306
H	1.19473	9.29258	21.91455
H	-0.00608	9.60919	20.62471
H	-0.08626	8.08343	21.55343

7. X-Ray Crystallography:

Single-crystal structure analyses were carried out on a Bruker Smart X2S diffractometer operating with Mo-K_α radiation (λ= 0.71073 Å). Further crystallographic and refinement data can be found in Table 1. The structures were solved by direct methods (SHELXS-97)[6] and refined by full-matrix least squares on F^2 (SHELXL-97).[7] The H atoms were calculated geometrically, and a riding model was applied in the refinement process. The Flack parameter for the crystal structure of **17a** is 0.7(13) and hence not suitable for the determination of the absolute configuration. Therefore, the configurations of the three stereogenic carbon atoms were deduced from the known *R*-configuration of the starting material **6C**. The presence of the bromine atom permitted the determination of the absolute configuration of **19f** by refining the Flack parameter [0.002(13)]. The determined configuration is in agreement with the absolute configuration of stereogenic center of the starting material **6**C. The oxygen atoms of the oxazolidine rings of both compounds were found to be disordered over two positions

[6] (a) G. M. Sheldrick, *SHELXS-97, Program for the Solution of Crystal Structures*, Göttingen, Germany, 1997. See also: G. M. Sheldrick, *Acta Crystallographica*, 1990, **A46**, 467-473.
[7] (a) G. M. Sheldrick, *SHELXL-97, Program for crystal structure refinement*, Göttingen, Germany, 1997. See also: G. M. Sheldrick, *Acta Crystallographica*, 2008, **A64**, 112-122.

(refined occupancy 55:45 for **17a** and 65:35 for **19f**). CCDC 1023711 contains the supplementary crystallographic data for compound **17a**. CCDC 1030561 contains the supplementary crystallographic data for compound **19f**. These data can be obtained free of charge from The Cambrige Crystallographic Data Centre at www.ccdc.cam.ac.uk.

Table 1: Crystal Data and Data Collection and Structure Refinement Details for Compounds **17a** and **19f**.

**Crystal Data**	**17a**	**19f**
Empirical formula	$C_{20}H_{21}NO_3$	$C_{25}H_{29}BrN_2O_4$
Formula weight	323.38	501.41
Crystal size (mm)	0.87 × 0.48 × 0.42	0.67 × 0.41 × 0.30
Crystal system	orthorhombic	orthorhombic
Space group	$P2_12_12_1$	$P2_12_12_1$
a (Å)	7.4058(6)	7.880(2)
b (Å)	12.3833(12)	12.465(3)
c (Å)	18.9412(18)	25.404(4)
V (Å^3)	1737.1(3)	2495.2(9)
D_{calcd} (g cm^{-1})	1.237	1.335
Z	4	4
μ (mm^{-1})	0.08	1.68
T (K)	233	300
θ range (°)	2.7 – 25.1	2.3 – 23.3
No. of reflections measured	19092	17475
No. of independent reflections	3073	3589
Parameters refined/restraints	230/0	304/0
R_{int}	0.053	0.079
Absorption correction	multi scan	multi scan
T_{min}, T_{max}	0.93, 0.97	0.44, 0.63
Largest diff. peak and hole (e Å^{-3})	0.15 / -0.16	0.20 / -0.30
Flack parameter	0.7(13)	0.002(13)
Final *R* indices [$I \geq 2\sigma(I)$]	$R_1 = 0.035$	$R_1 = 0.047$
	$wR_2 = 0.098$	$wR_2 = 0.108$
CCDC no.	1023711	1030561

8. Copies of selected NMR spectra:

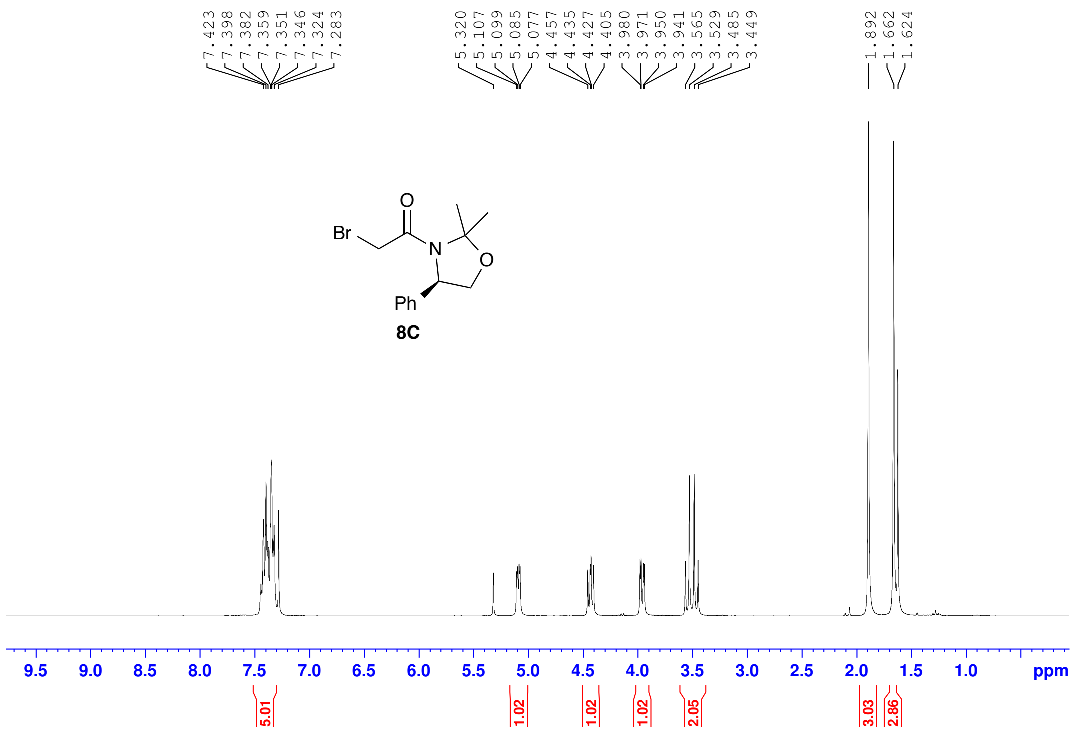

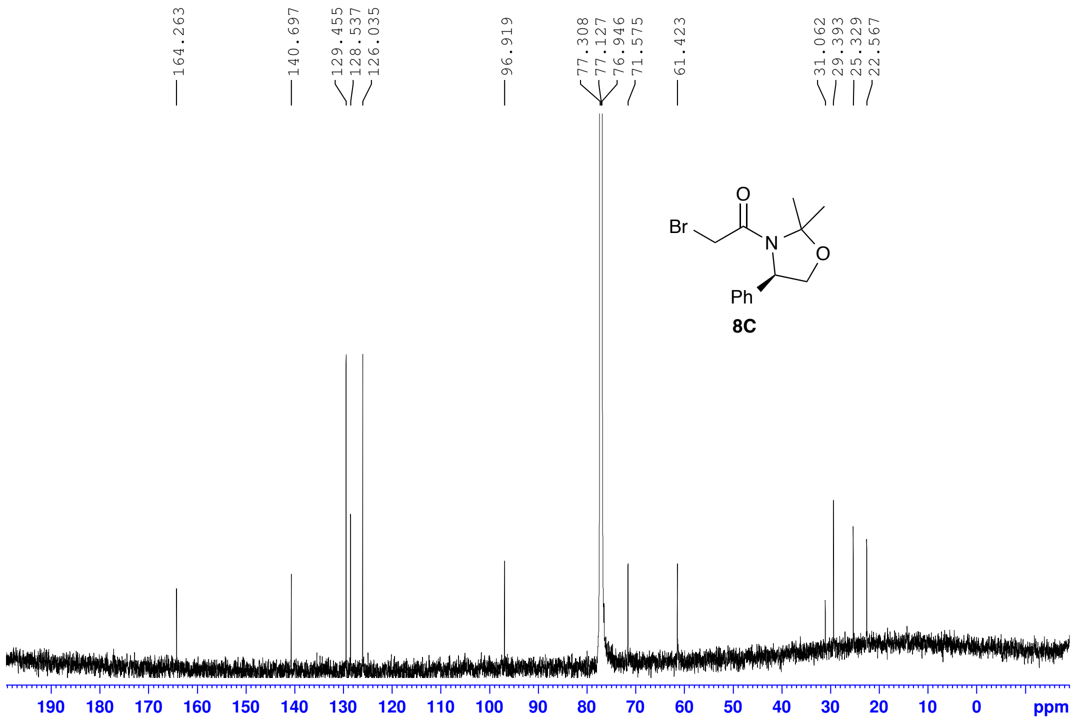

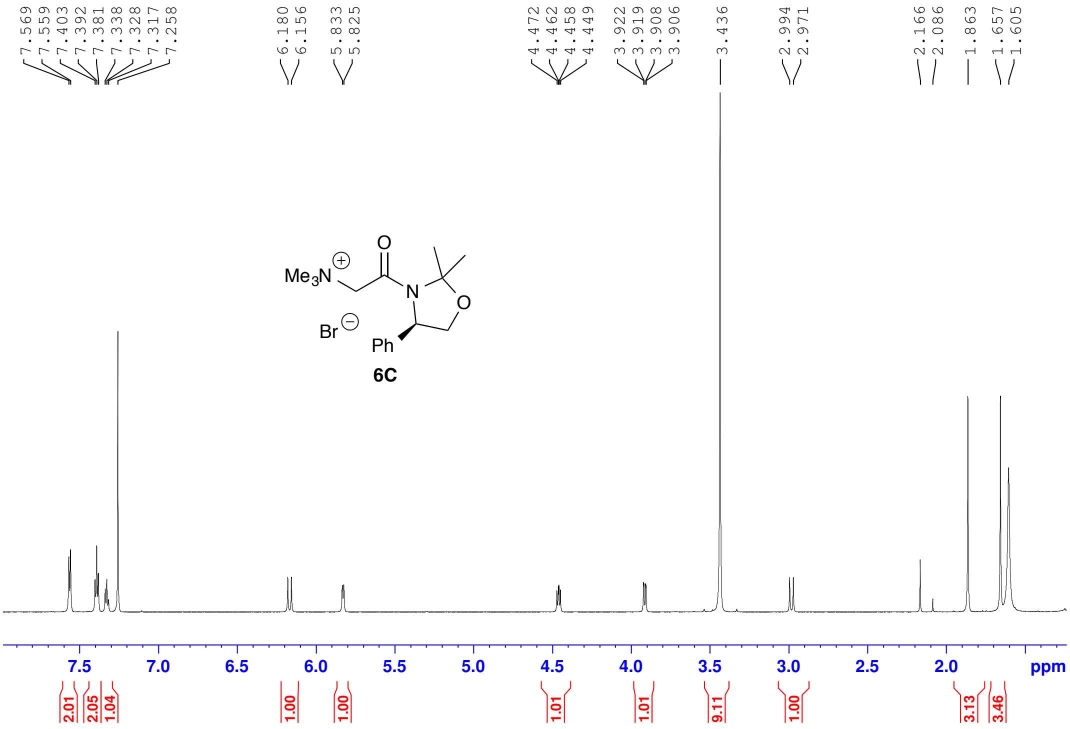
7.569 7.559 7.403 7.392 7.381 7.338 7.328 7.317 7.258
6.180 6.156
5.833 5.825
4.472 4.462 4.458 4.449
3.922 3.919 3.908 3.906
3.436
2.994 2.971
2.166 2.086
1.863
1.657 1.605
Me3N
Br
Ph
O
N
O
6C
2.01 2.05 1.04 1.00 1.00 1.01 1.01 9.11 1.00 3.13 3.46
7.5 7.0 6.5 6.0 5.5 5.0 4.5 4.0 3.5 3.0 2.5 2.0 ppm

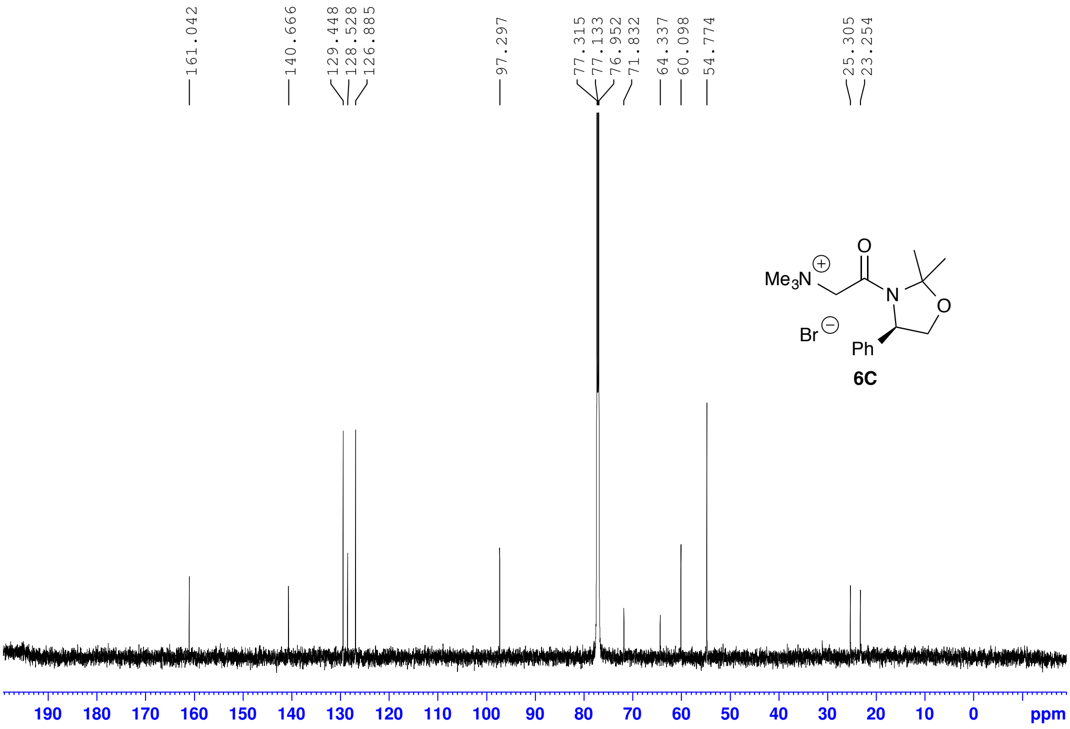
161.042
140.666
129.448 128.528 126.885
97.297
77.315 77.133 76.952 71.832
64.337
60.098
54.774
25.305 23.254
Me3N
Br
Ph
O
N
O
6C
190 180 170 160 150 140 130 120 110 100 90 80 70 60 50 40 30 20 10 0 ppm

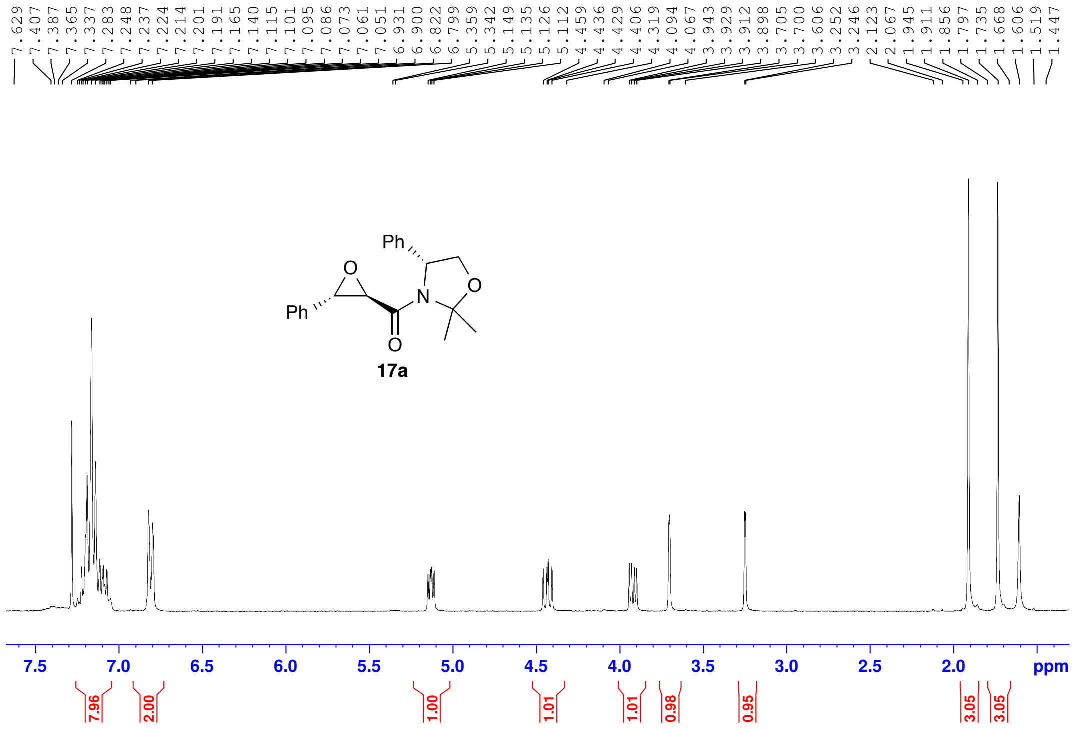
7.629
7.407
7.387
7.365
7.337
7.283
7.248
7.237
7.224
7.214
7.201
7.191
7.165
7.140
7.115
7.101
7.095
7.086
7.073
7.061
7.051
6.931
6.900
6.822
6.799
5.359
5.342
5.149
5.135
5.126
5.112
4.459
4.436
4.429
4.406
4.319
4.094
4.067
3.943
3.929
3.912
3.898
3.705
3.700
3.606
3.252
3.246
2.123
2.067
1.945
1.911
1.856
1.797
1.735
1.668
1.606
1.519
1.447
Ph
O
N
O
Ph
O
17a
7.5
7.0
6.5
6.0
5.5
5.0
4.5
4.0
3.5
3.0
2.5
2.0
ppm
7.96
2.00
1.00
1.01
1.01
0.98
0.95
3.05
3.05

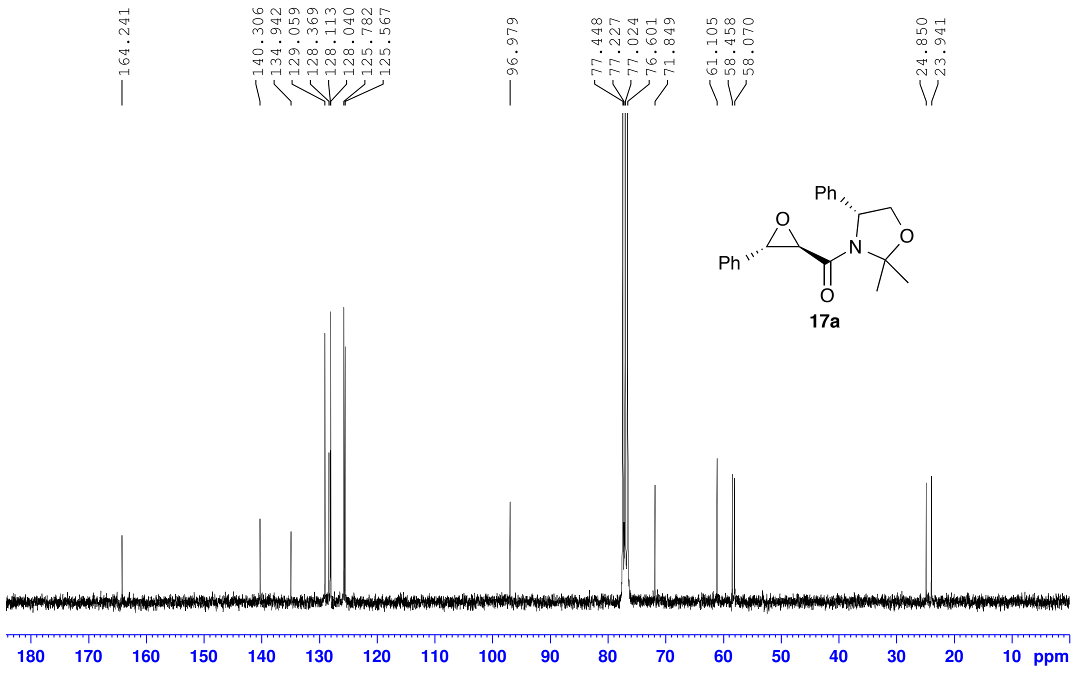
164.241
140.306
134.942
129.059
128.369
128.113
128.040
125.782
125.567
96.979
77.448
77.227
77.024
76.601
71.849
61.105
58.458
58.070
24.850
23.941
Ph
O
N
O
Ph
O
17a
180
170
160
150
140
130
120
110
100
90
80
70
60
50
40
30
20
10
ppm

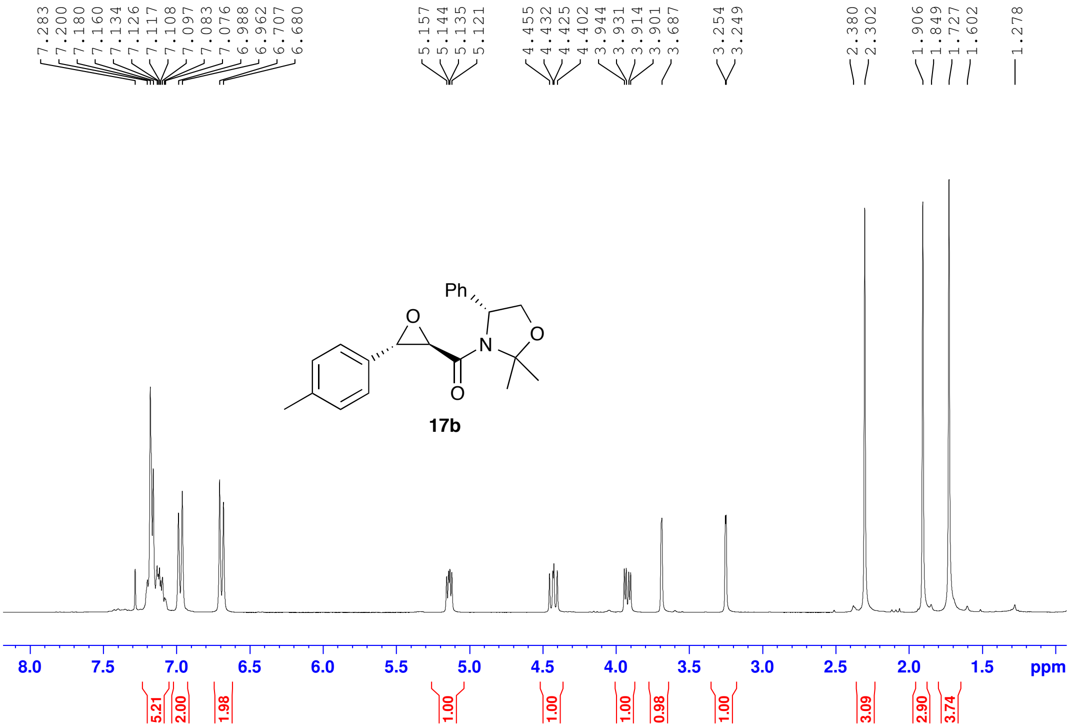
7.283 7.200 7.180 7.160 7.134 7.126 7.117 7.108 7.097 7.083 7.076 6.988 6.962 6.707 6.680
5.157 5.144 5.135 5.121
4.455 4.432 4.425 4.402 3.944 3.931 3.914 3.901 3.687
3.254 3.249
2.380 2.302
1.906 1.849 1.727 1.602
1.278
Ph
O
N
O
O
17b
8.0 7.5 7.0 6.5 6.0 5.5 5.0 4.5 4.0 3.5 3.0 2.5 2.0 1.5 ppm
5.21 2.00 1.98 1.00 1.00 1.00 0.98 1.00 3.09 2.90 3.74

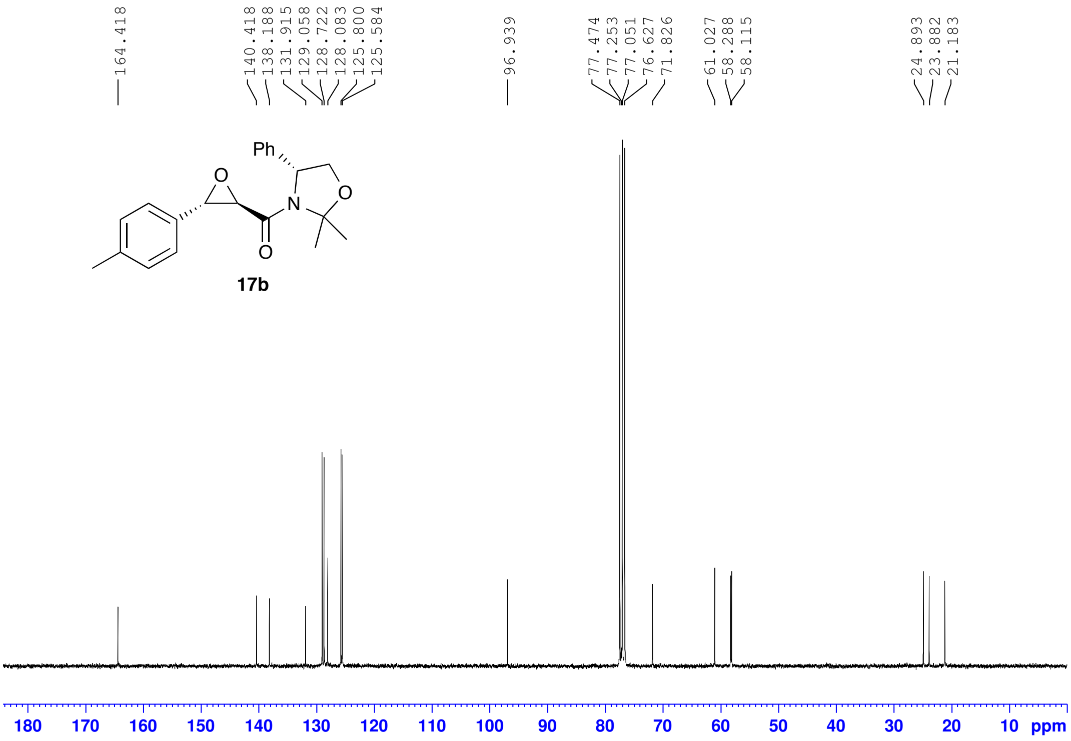
164.418
140.418 138.188 131.915 129.058 128.722 128.083 125.800 125.584
96.939
77.474 77.253 77.051 76.627 71.826
61.027 58.288 58.115
24.893 23.882 21.183
Ph
O
N
O
O
17b
180 170 160 150 140 130 120 110 100 90 80 70 60 50 40 30 20 10 ppm

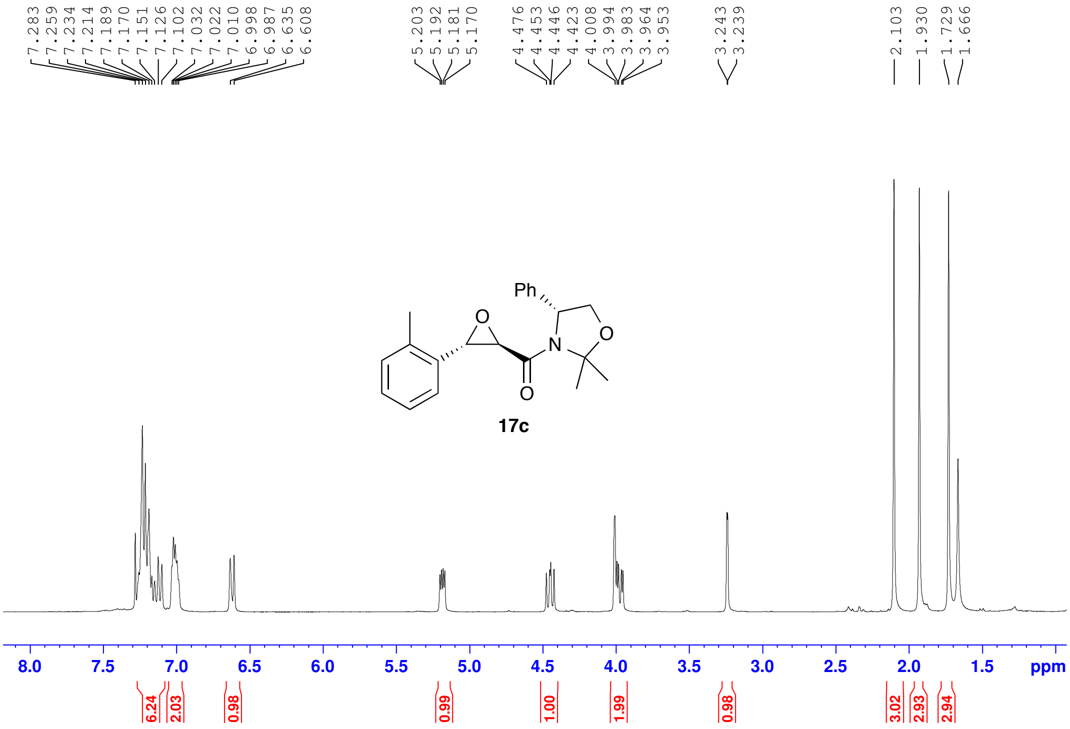
7.283 7.259 7.234 7.214 7.189 7.170 7.151 7.126 7.102 7.032 7.022 7.010 6.998 6.987 6.635 6.608
5.203 5.192 5.181 5.170
4.476 4.453 4.446 4.423 4.008 3.994 3.983 3.964 3.953
3.243 3.239
2.103 1.930 1.729 1.666
Ph
O
N
O
O
17c
8.0 7.5 7.0 6.5 6.0 5.5 5.0 4.5 4.0 3.5 3.0 2.5 2.0 1.5 ppm
6.24 2.03 0.98 0.99 1.00 1.99 0.98 3.02 2.93 2.94

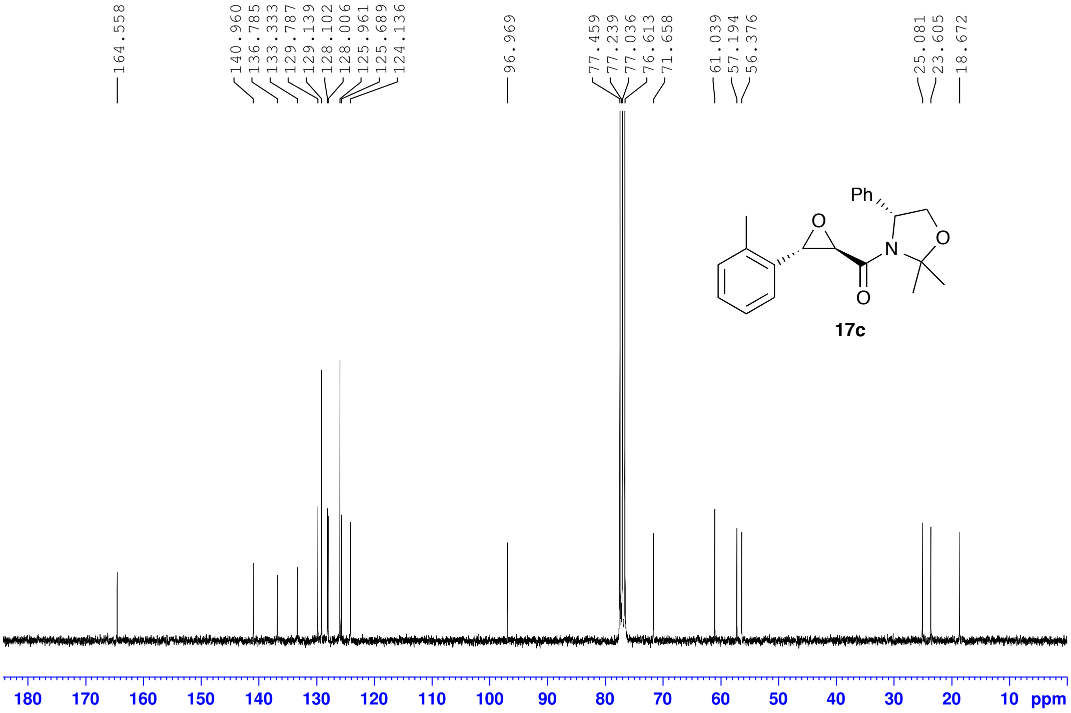
164.558
140.960 136.785 133.333 129.787 129.139 128.102 128.006 125.961 125.689 124.136
96.969
77.459 77.239 77.036 76.613 71.658
61.039 57.194 56.376
25.081 23.605 18.672
Ph
O
N
O
O
17c
180 170 160 150 140 130 120 110 100 90 80 70 60 50 40 30 20 10 ppm

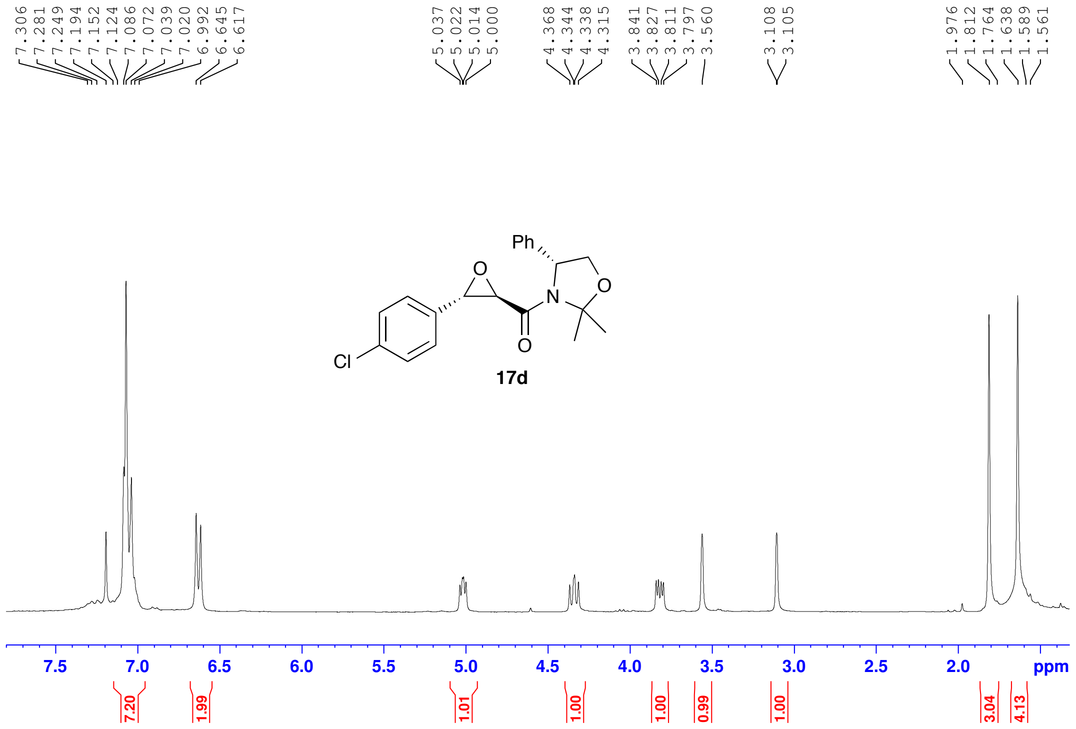
7.306 7.281 7.249 7.194 7.152 7.124 7.086 7.072 7.039 7.020 6.992 6.645 6.617
5.037 5.022 5.014 5.000
4.368 4.344 4.338 4.315
3.841 3.827 3.811 3.797 3.560
3.108 3.105
1.976 1.812 1.764 1.638 1.589 1.561
Ph
O
N
O
Cl
O
17d
7.5 7.0 6.5 6.0 5.5 5.0 4.5 4.0 3.5 3.0 2.5 2.0 ppm
7.20 1.99 1.01 1.00 1.00 0.99 1.00 3.04 4.13

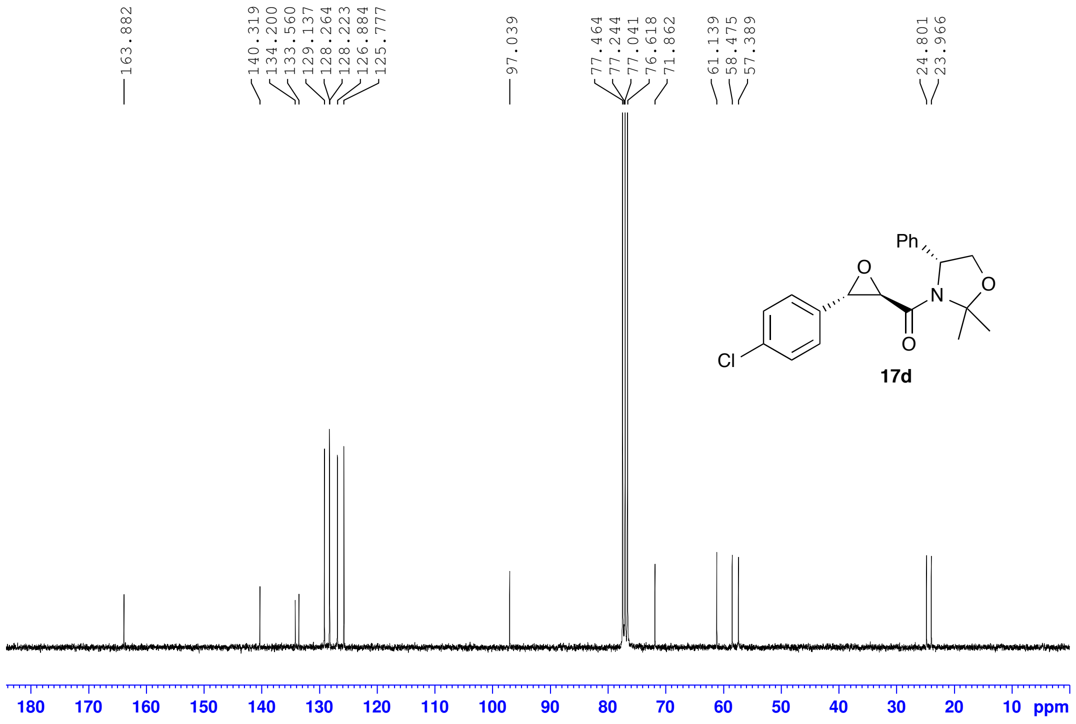
163.882
140.319 134.200 133.560 129.137 128.264 128.223 126.884 125.777
97.039
77.464 77.244 77.041 76.618 71.862
61.139 58.475 57.389
24.801 23.966
Ph
O
N
O
Cl
O
17d
180 170 160 150 140 130 120 110 100 90 80 70 60 50 40 30 20 10 ppm

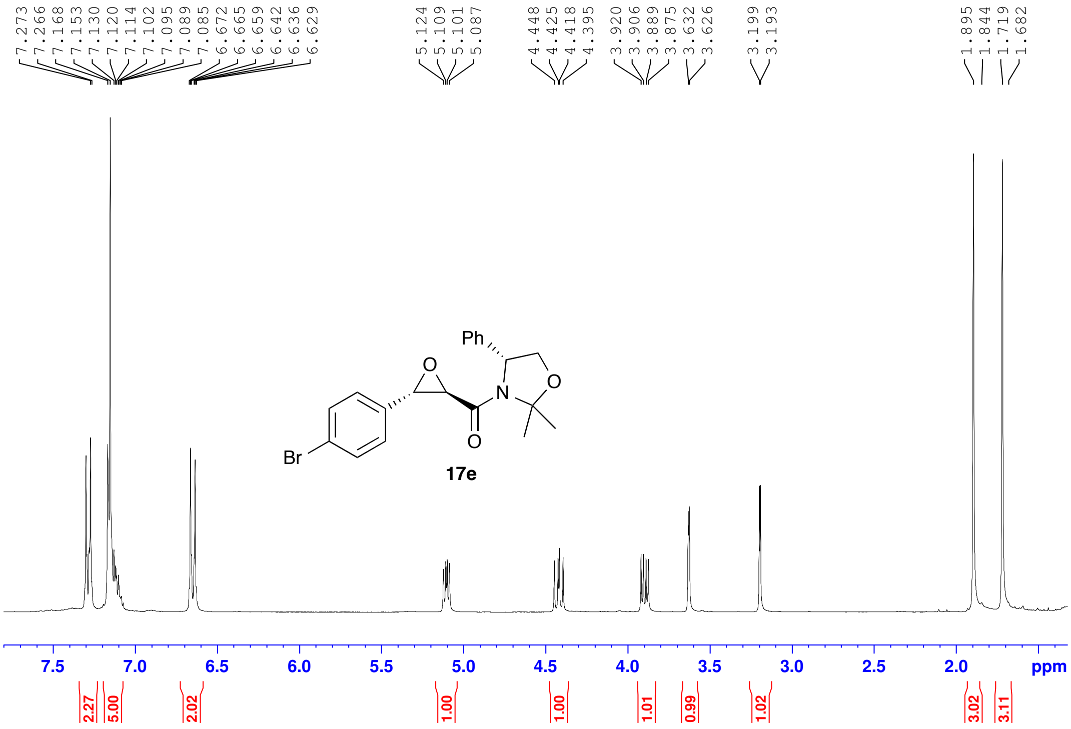
7.273 7.266 7.168 7.153 7.130 7.120 7.114 7.102 7.095 7.089 7.085 6.672 6.665 6.659 6.642 6.636 6.629
5.124 5.109 5.101 5.087
4.448 4.425 4.418 4.395
3.920 3.906 3.889 3.875 3.632 3.626
3.199 3.193
1.895 1.844 1.719 1.682
Ph
O
N
O
O
Br
17e
7.5 7.0 6.5 6.0 5.5 5.0 4.5 4.0 3.5 3.0 2.5 2.0 ppm
2.27 5.00 2.02 1.00 1.00 1.01 0.99 1.02 3.02 3.11

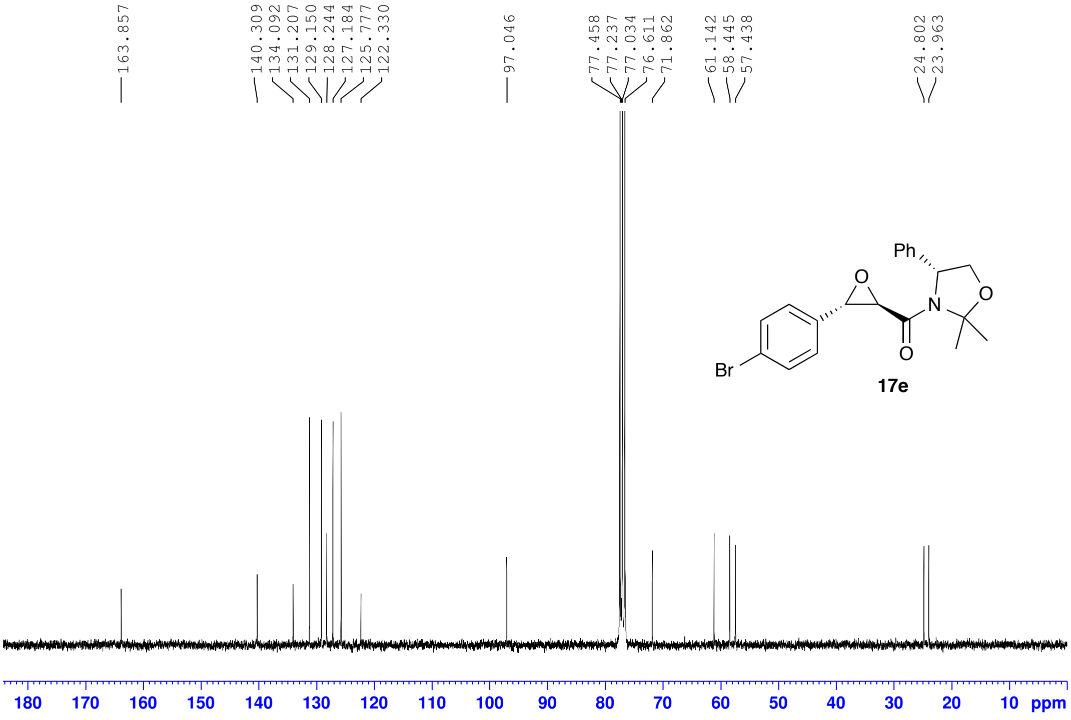
163.857
140.309 134.092 131.207 129.150 128.244 127.184 125.777 122.330
97.046
77.458 77.237 77.034 76.611 71.862
61.142 58.445 57.438
24.802 23.963
Ph
O
N
O
O
Br
17e
180 170 160 150 140 130 120 110 100 90 80 70 60 50 40 30 20 10 ppm

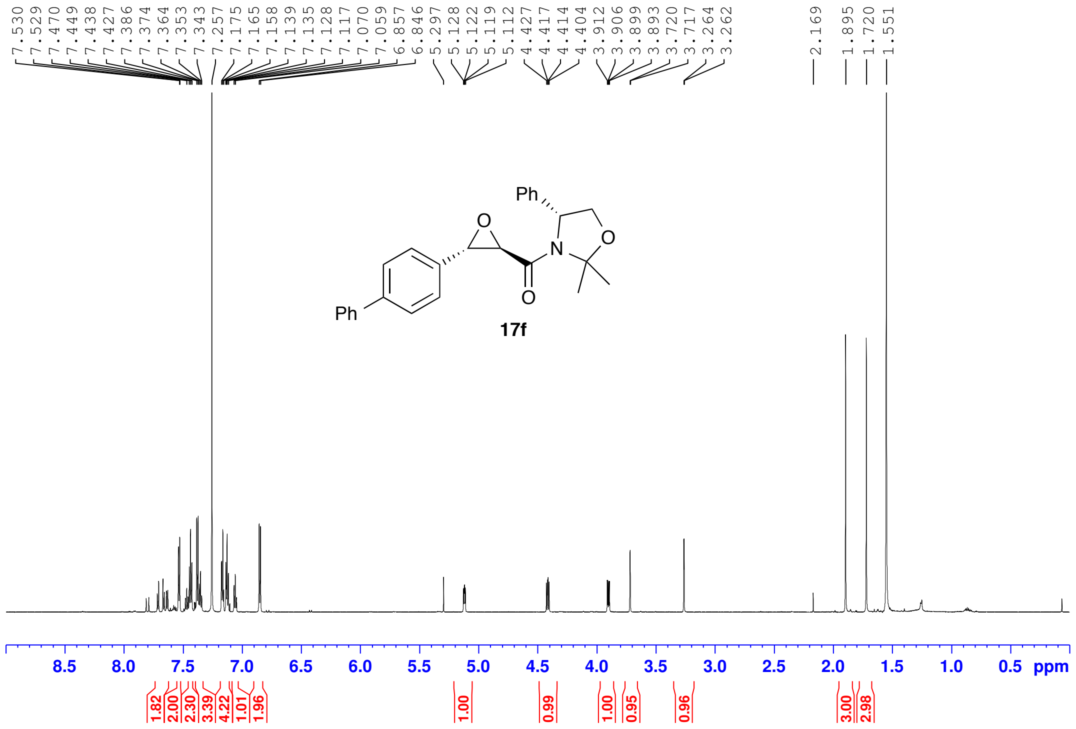
7.530
7.529
7.470
7.449
7.438
7.427
7.386
7.374
7.364
7.353
7.343
7.257
7.175
7.165
7.158
7.139
7.135
7.128
7.117
7.070
7.059
6.857
6.846
5.297
5.128
5.122
5.119
5.112
4.427
4.417
4.414
4.404
3.912
3.906
3.899
3.893
3.720
3.717
3.264
3.262
2.169
1.895
1.720
1.551
Ph
O
N
O
Ph
O
17f
8.5 8.0 7.5 7.0 6.5 6.0 5.5 5.0 4.5 4.0 3.5 3.0 2.5 2.0 1.5 1.0 0.5 ppm
1.82
2.00
2.30
3.39
4.22
1.01
1.96
1.00
0.99
1.00
0.95
0.96
3.00
2.98

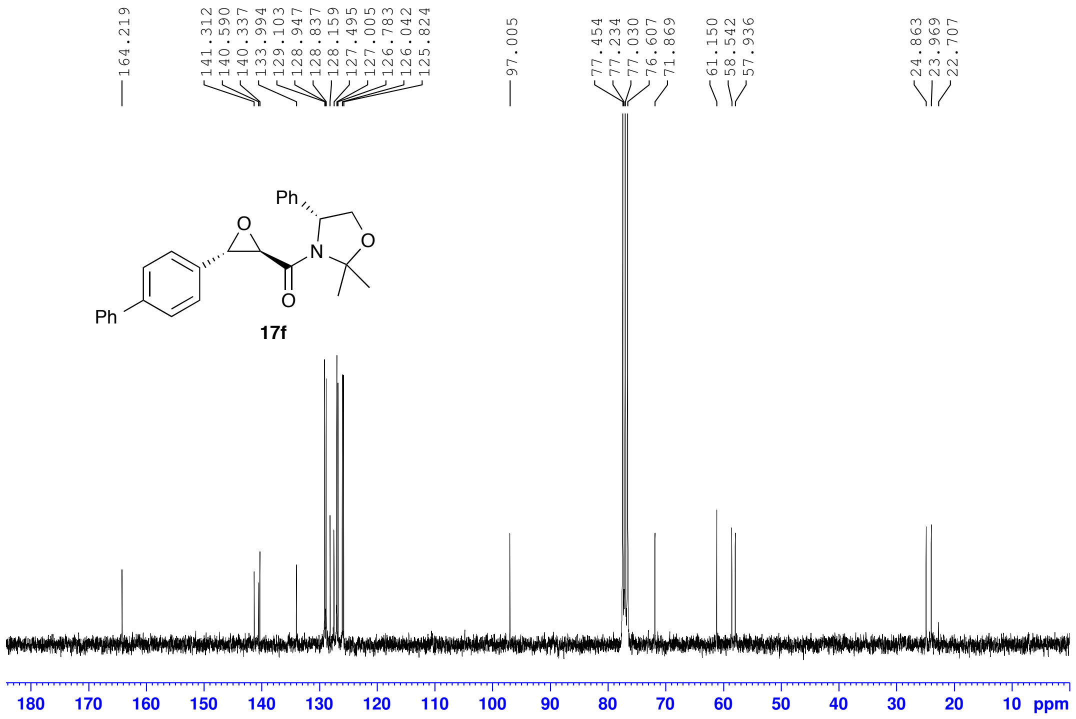
164.219
141.312
140.590
140.337
133.994
129.103
128.947
128.837
128.159
127.495
127.005
126.783
126.042
125.824
97.005
77.454
77.234
77.030
76.607
71.869
61.150
58.542
57.936
24.863
23.969
22.707
Ph
O
N
O
Ph
O
17f
180 170 160 150 140 130 120 110 100 90 80 70 60 50 40 30 20 10 ppm

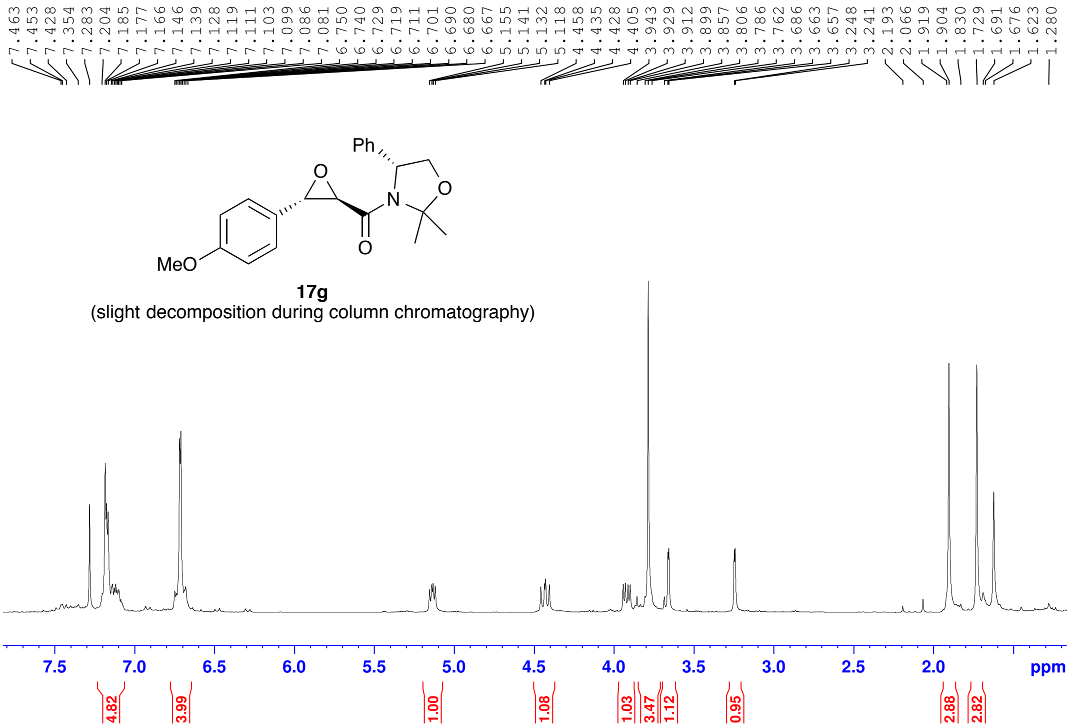
7.463
7.453
7.428
7.354
7.283
7.204
7.185
7.177
7.166
7.146
7.139
7.128
7.119
7.111
7.103
7.099
7.086
7.081
6.750
6.740
6.729
6.719
6.711
6.701
6.690
6.680
6.667
5.155
5.141
5.132
5.118
4.458
4.435
4.428
4.405
3.943
3.929
3.912
3.899
3.857
3.806
3.786
3.762
3.686
3.663
3.657
3.248
3.241
2.193
2.066
1.919
1.904
1.830
1.729
1.691
1.676
1.623
1.280
Ph
O
N
O
O
MeO
17g
(slight decomposition during column chromatography)
7.5
7.0
6.5
6.0
5.5
5.0
4.5
4.0
3.5
3.0
2.5
2.0
ppm
4.82
3.99
1.00
1.08
1.03
3.47
1.12
0.95
2.88
2.82

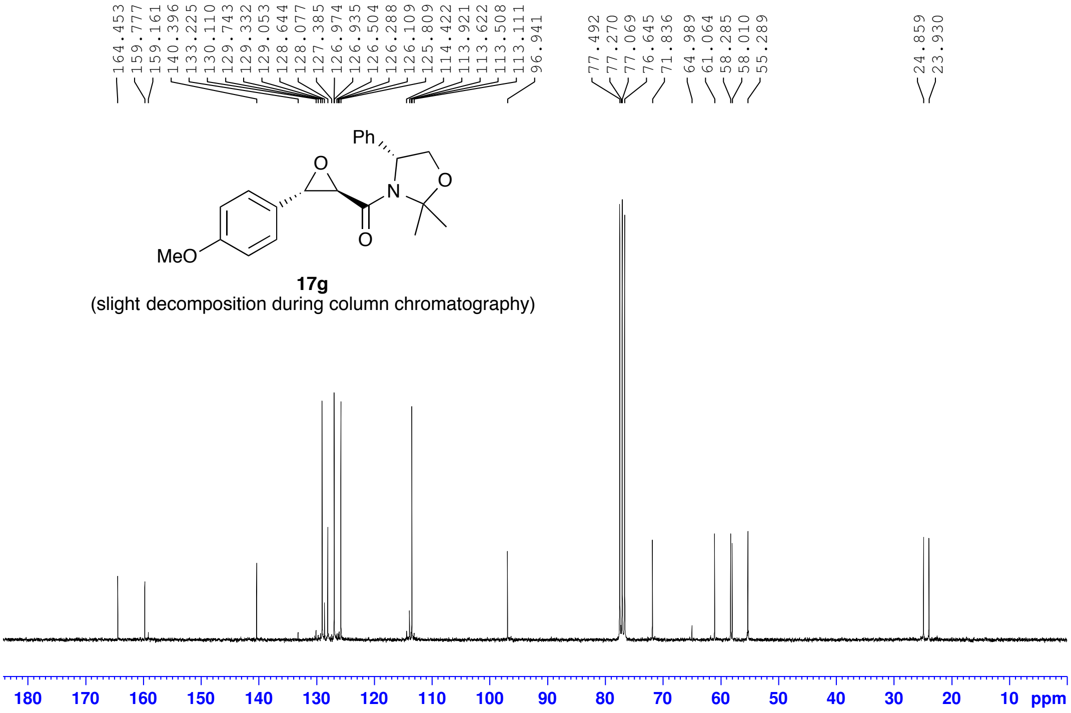
164.453
159.777
159.161
140.396
133.225
130.110
129.743
129.332
129.053
128.644
128.077
127.385
126.974
126.935
126.504
126.288
126.109
125.809
114.422
113.921
113.622
113.508
113.111
96.941
77.492
77.270
77.069
76.645
71.836
64.989
61.064
58.285
58.010
55.289
24.859
23.930
Ph
O
N
O
O
MeO
17g
(slight decomposition during column chromatography)
180
170
160
150
140
130
120
110
100
90
80
70
60
50
40
30
20
10
ppm

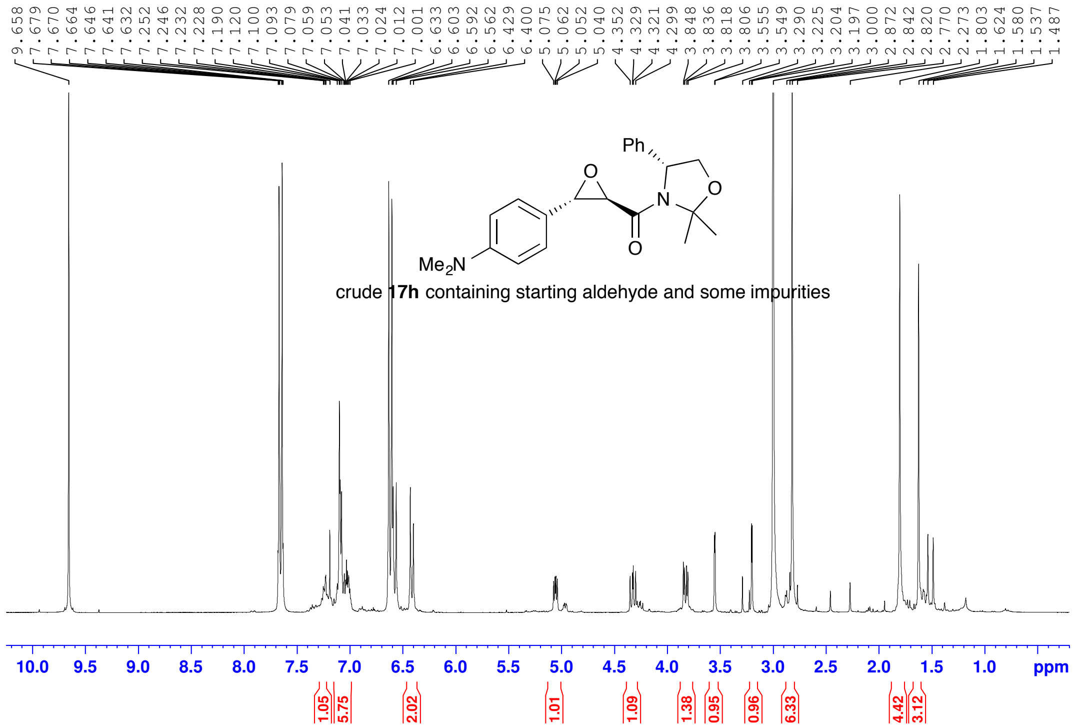
9.658 7.679 7.670 7.664 7.646 7.641 7.632 7.252 7.246 7.232 7.228 7.190 7.120 7.100 7.093 7.079 7.059 7.053 7.041 7.033 7.024 7.012 7.001 6.633 6.603 6.592 6.562 6.429 6.400 5.075 5.062 5.052 5.040 4.352 4.329 4.321 4.299 3.848 3.836 3.818 3.806 3.555 3.549 3.290 3.225 3.204 3.197 3.000 2.872 2.842 2.820 2.770 2.273 1.803 1.624 1.580 1.537 1.487
Ph
O
N
O
O
Me2N
crude **17h** containing starting aldehyde and some impurities
10.0 9.5 9.0 8.5 8.0 7.5 7.0 6.5 6.0 5.5 5.0 4.5 4.0 3.5 3.0 2.5 2.0 1.5 1.0 ppm
1.05 5.75 2.02 1.01 1.09 1.38 0.95 0.96 6.33 4.42 3.12

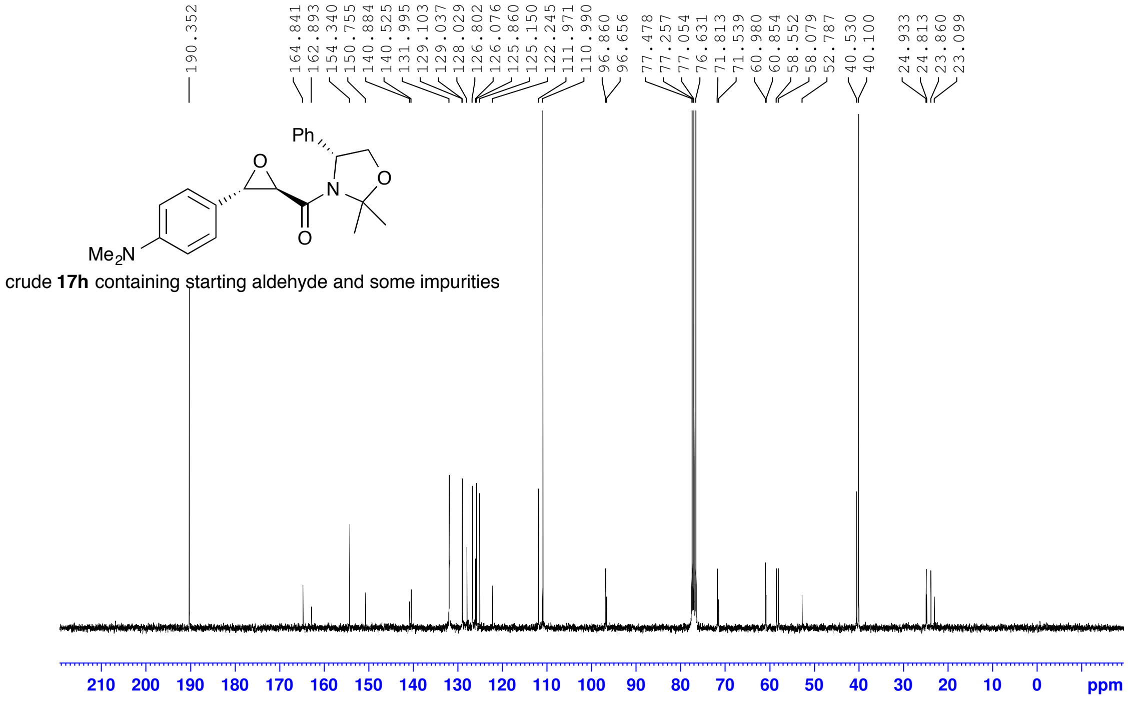
190.352
164.841 162.893 154.340 150.755 140.884 140.525 131.995 129.103 129.037 128.029 126.802 126.076 125.860 125.150 122.245 111.971 110.990 96.860 96.656 77.478 77.257 77.054 76.631 71.813 71.539 60.980 60.854 58.552 58.079 52.787 40.530 40.100 24.933 24.813 23.860 23.099
Ph
O
N
O
O
Me2N
crude **17h** containing starting aldehyde and some impurities
210 200 190 180 170 160 150 140 130 120 110 100 90 80 70 60 50 40 30 20 10 0 ppm

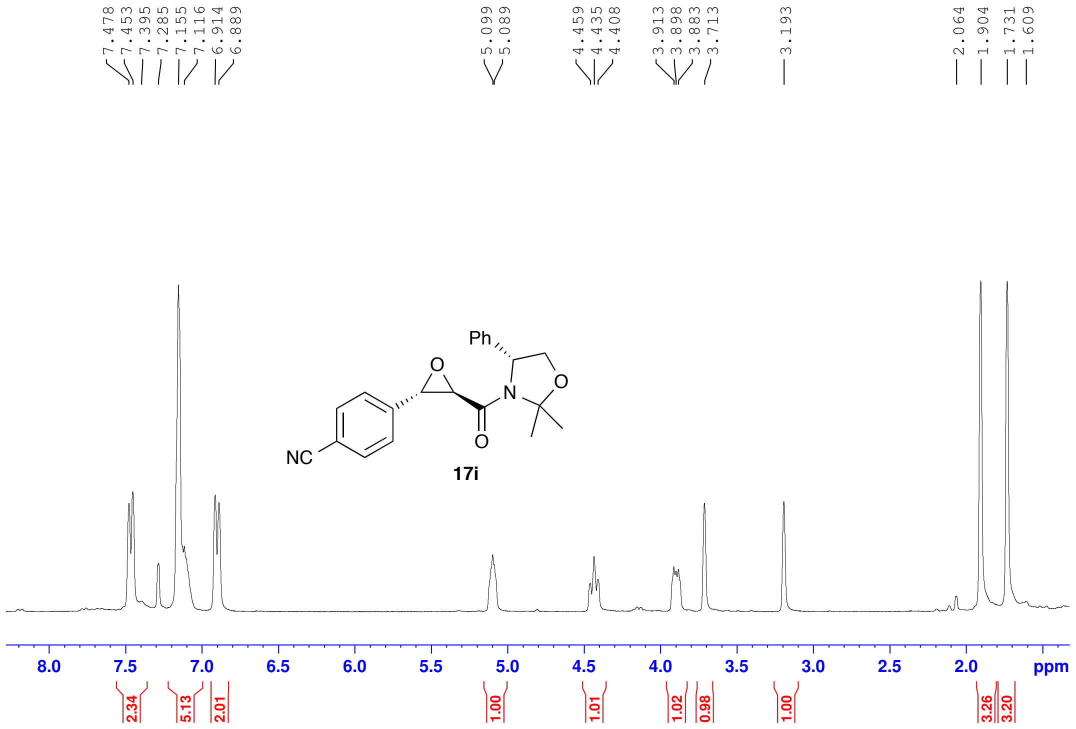
7.478
7.453
7.395
7.285
7.155
7.116
6.914
6.889
5.099
5.089
4.459
4.435
4.408
3.913
3.898
3.883
3.713
3.193
2.064
1.904
1.731
1.609
Ph
O
N
O
NC
O
17i
8.0
7.5
7.0
6.5
6.0
5.5
5.0
4.5
4.0
3.5
3.0
2.5
2.0
ppm
2.34
5.13
2.01
1.00
1.01
1.02
0.98
1.00
3.26
3.20

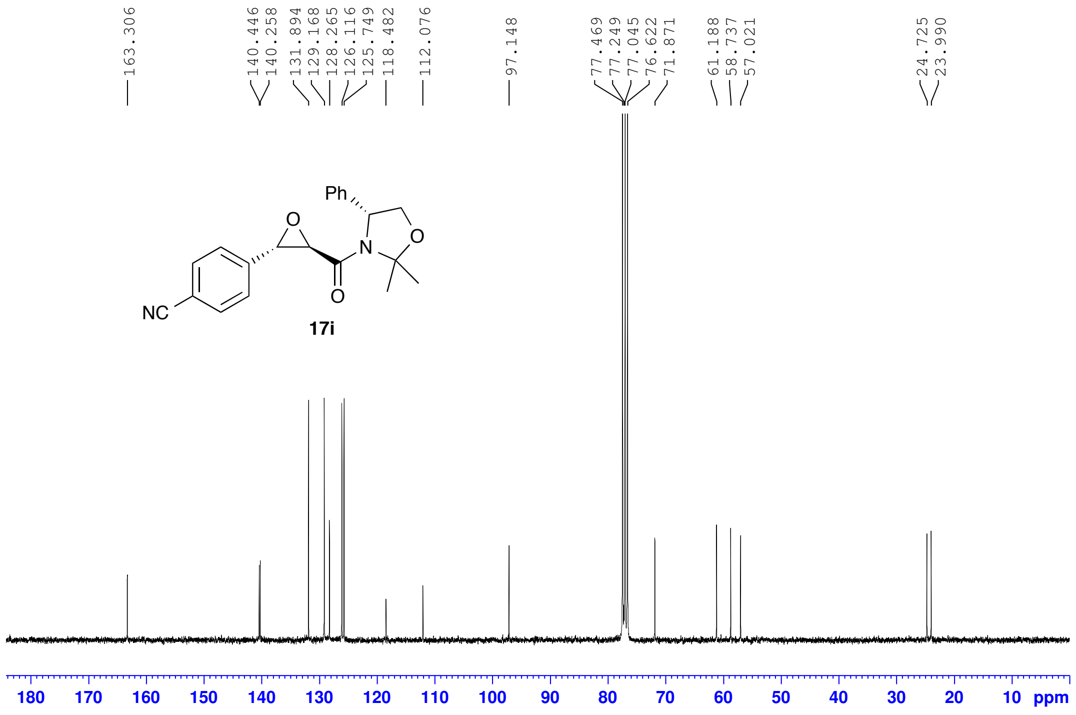
163.306
140.446
140.258
131.894
129.168
128.265
126.116
125.749
118.482
112.076
97.148
77.469
77.249
77.045
76.622
71.871
61.188
58.737
57.021
24.725
23.990
Ph
O
N
O
NC
O
17i
180
170
160
150
140
130
120
110
100
90
80
70
60
50
40
30
20
10
ppm

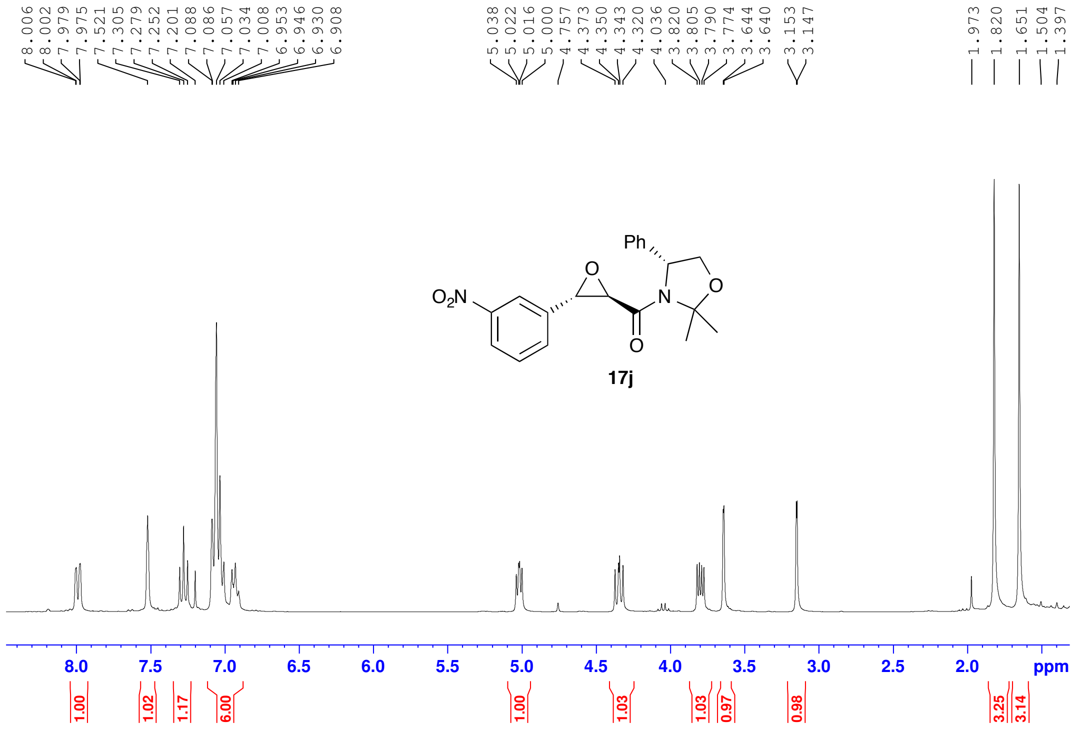
8.006 8.002 7.979 7.975 7.521 7.305 7.279 7.252 7.201 7.088 7.086 7.057 7.034 7.008 6.953 6.946 6.930 6.908
5.038 5.022 5.016 5.000 4.757 4.373 4.350 4.343 4.320 4.036 3.820 3.805 3.790 3.774 3.644 3.640 3.153 3.147
1.973 1.820 1.651 1.504 1.397
Ph
O
O_2N
N
O
O
17j
8.0 7.5 7.0 6.5 6.0 5.5 5.0 4.5 4.0 3.5 3.0 2.5 2.0 ppm
1.00 1.02 1.17 6.00 1.00 1.03 1.03 0.97 0.98 3.25 3.14

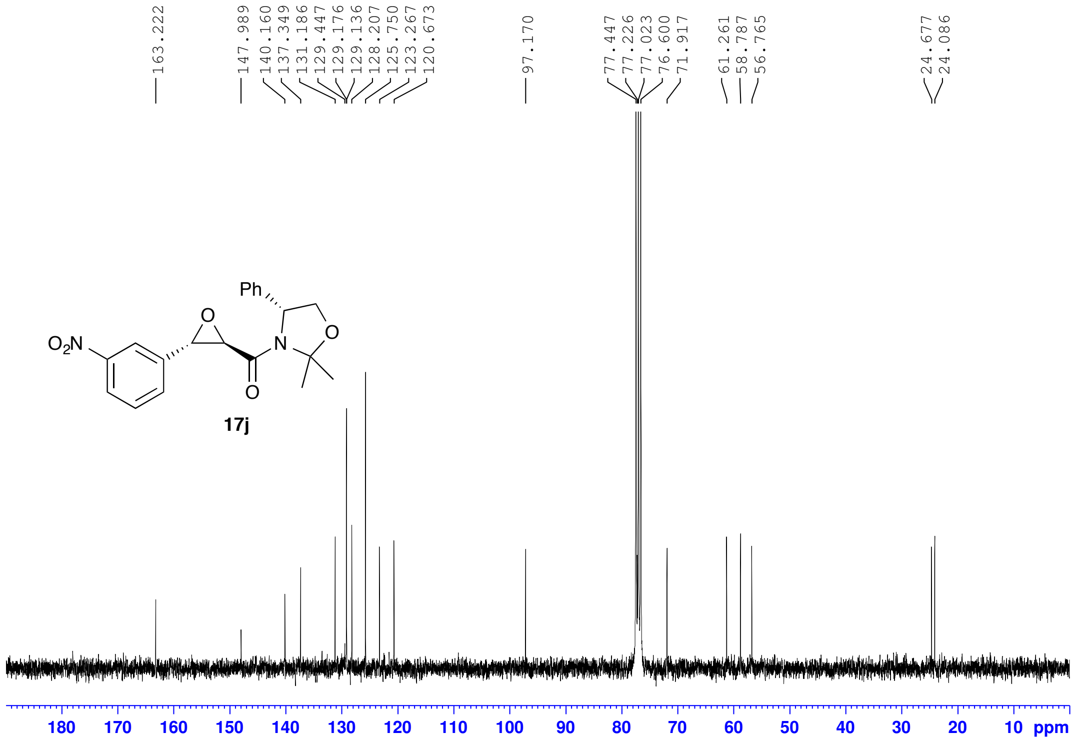
163.222
147.989 140.160 137.349 131.186 129.447 129.176 129.136 128.207 125.750 123.267 120.673
97.170
77.447 77.226 77.023 76.600 71.917
61.261 58.787 56.765
24.677 24.086
Ph
O
O_2N
N
O
O
17j
180 170 160 150 140 130 120 110 100 90 80 70 60 50 40 30 20 10 ppm

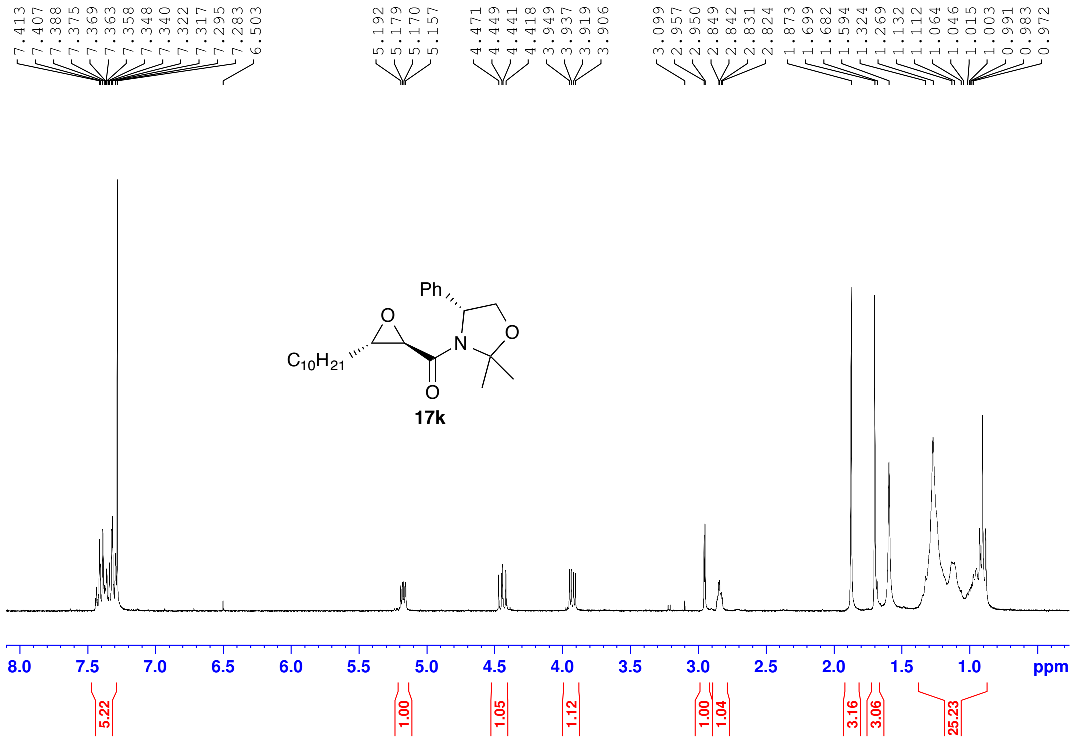
7.413 7.407 7.388 7.375 7.369 7.363 7.358 7.348 7.340 7.322 7.317 7.295 7.283 6.503
5.192 5.179 5.170 5.157
4.471 4.449 4.441 4.418 3.949 3.937 3.919 3.906
3.099 2.957 2.950 2.849 2.842 2.831 2.824
1.873 1.699 1.682 1.594 1.324 1.269 1.132 1.112 1.064 1.046 1.015 1.003 0.991 0.983 0.972
Ph
O
N
O
O
$C_{10}H_{21}$
17k
8.0 7.5 7.0 6.5 6.0 5.5 5.0 4.5 4.0 3.5 3.0 2.5 2.0 1.5 1.0 ppm
5.22 1.00 1.05 1.12 1.00 1.04 3.16 3.06 25.23

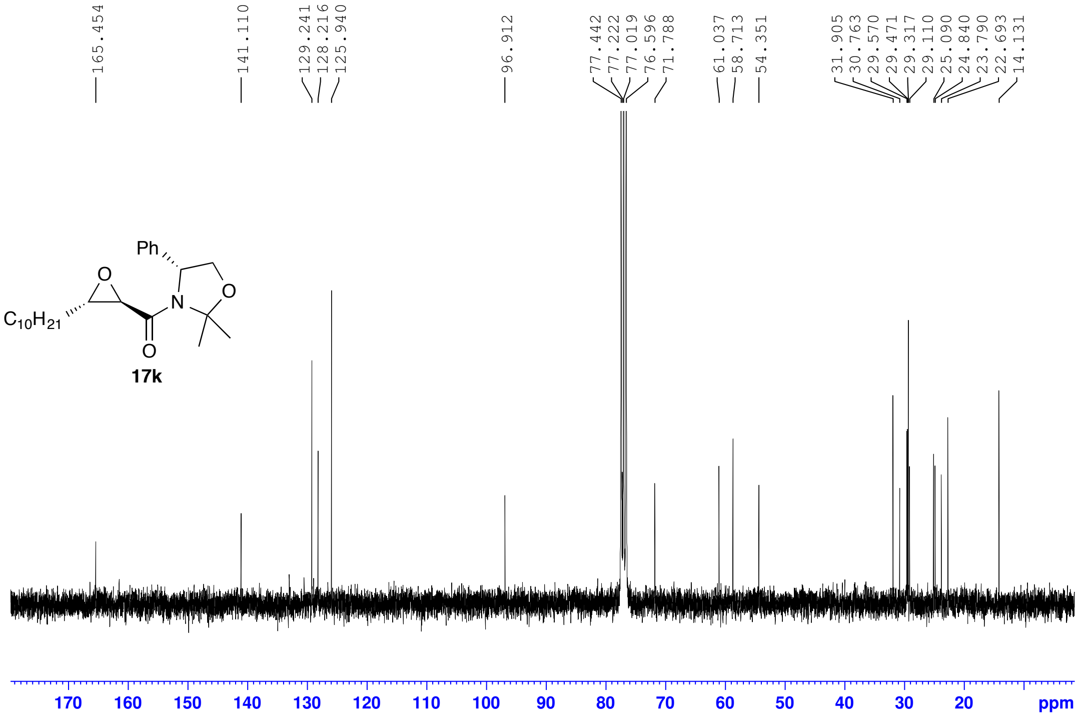
165.454
141.110
129.241 128.216 125.940
96.912
77.442 77.222 77.019 76.596 71.788
61.037 58.713 54.351
31.905 30.763 29.570 29.471 29.317 29.110 25.090 24.840 23.790 22.693 14.131
Ph
O
N
O
O
$C_{10}H_{21}$
17k
170 160 150 140 130 120 110 100 90 80 70 60 50 40 30 20 ppm

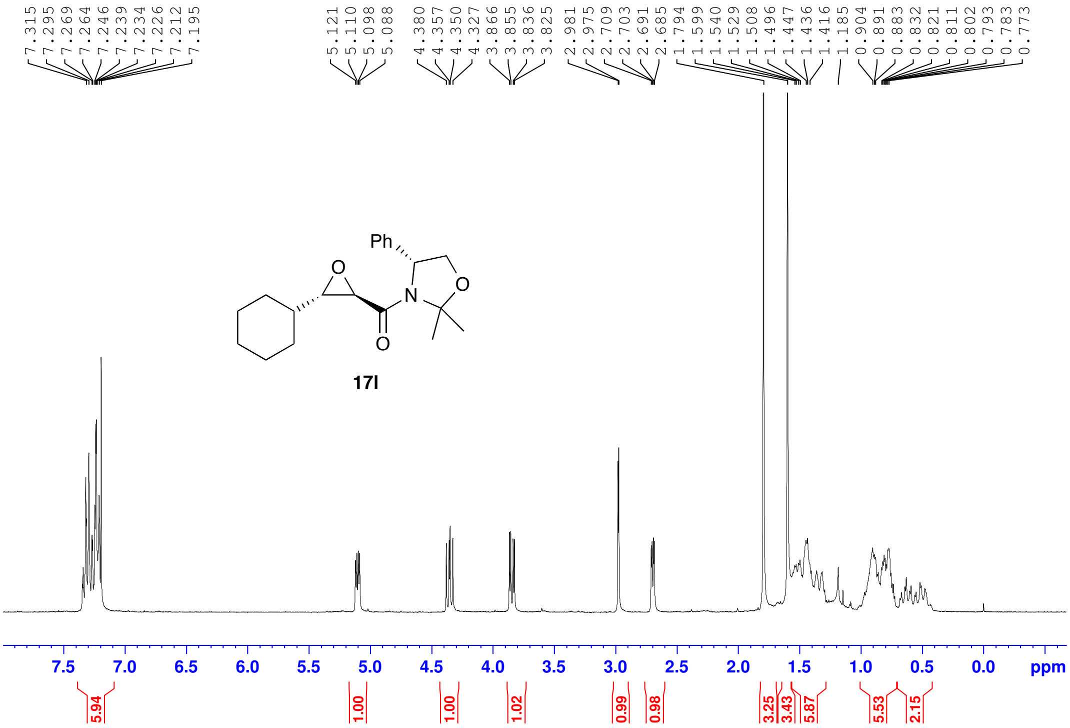
7.315 7.295 7.269 7.264 7.246 7.239 7.234 7.226 7.212 7.195
5.121 5.110 5.098 5.088
4.380 4.357 4.350 4.327 3.866 3.855 3.836 3.825
2.981 2.975 2.709 2.703 2.691 2.685
1.794 1.599 1.540 1.529 1.508 1.496 1.447 1.436 1.416 1.185
0.904 0.891 0.883 0.832 0.821 0.811 0.802 0.793 0.783 0.773
Ph
O
N
O
O
17l
7.5 7.0 6.5 6.0 5.5 5.0 4.5 4.0 3.5 3.0 2.5 2.0 1.5 1.0 0.5 0.0 ppm
5.94 1.00 1.00 1.02 0.99 0.98 3.25 3.43 5.87 5.53 2.15

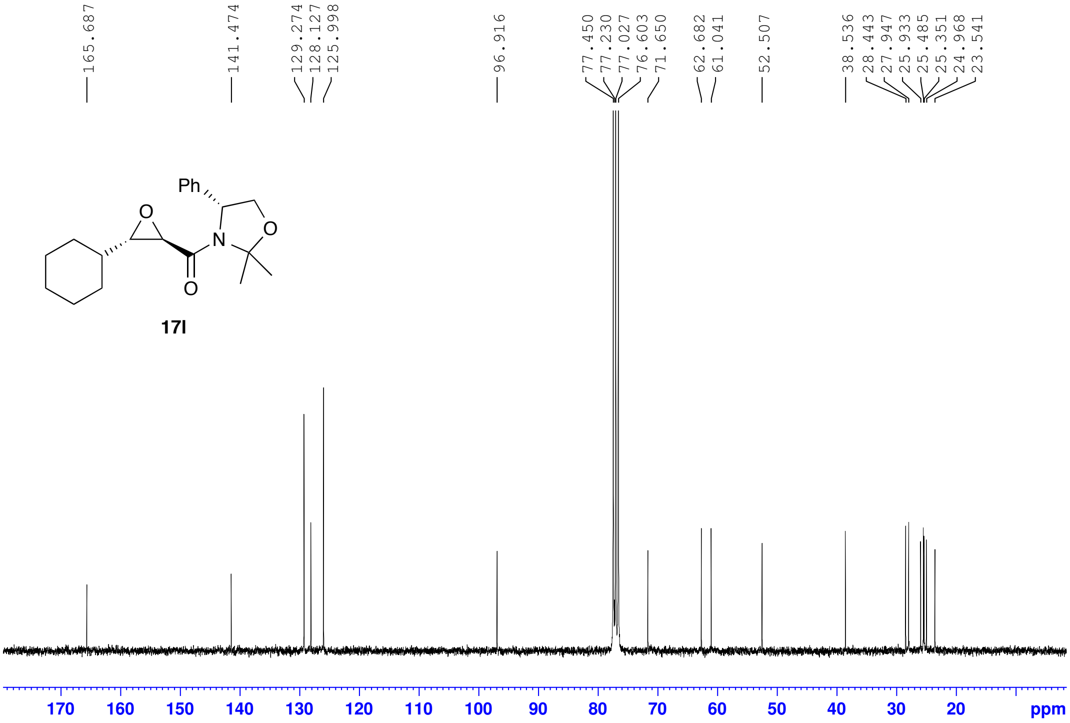
165.687
141.474
129.274 128.127 125.998
96.916
77.450 77.230 77.027 76.603 71.650
62.682 61.041
52.507
38.536 28.443 27.947 25.933 25.485 25.351 24.968 23.541
Ph
O
N
O
O
17l
170 160 150 140 130 120 110 100 90 80 70 60 50 40 30 20 ppm

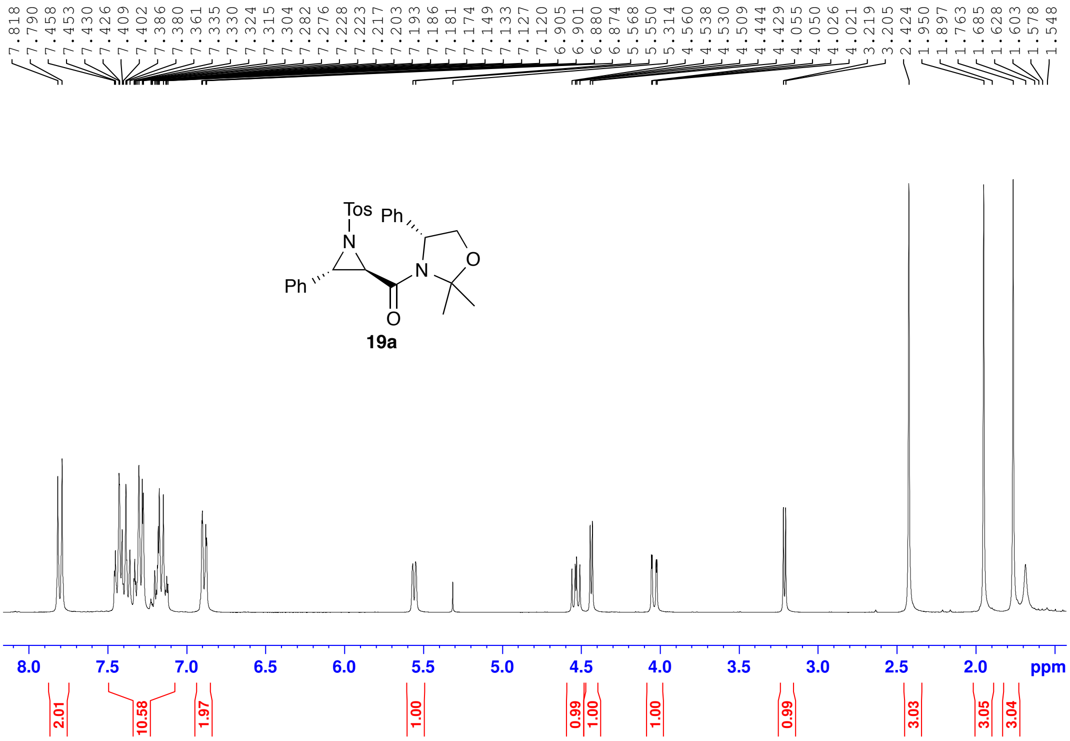
7.818 7.790 7.458 7.453 7.430 7.426 7.409 7.402 7.386 7.380 7.361 7.335 7.330 7.324 7.315 7.304 7.282 7.276 7.228 7.223 7.217 7.203 7.193 7.186 7.181 7.174 7.149 7.133 7.127 7.120 6.905 6.901 6.880 6.874 5.568 5.550 5.314 4.560 4.538 4.530 4.509 4.444 4.429 4.055 4.050 4.026 4.021 3.219 3.205 2.424 1.950 1.897 1.763 1.685 1.628 1.603 1.578 1.548
Tos
Ph
N
O
19a
8.0 7.5 7.0 6.5 6.0 5.5 5.0 4.5 4.0 3.5 3.0 2.5 2.0 ppm
2.01 10.58 1.97 1.00 0.99 1.00 1.00 0.99 3.03 3.05 3.04

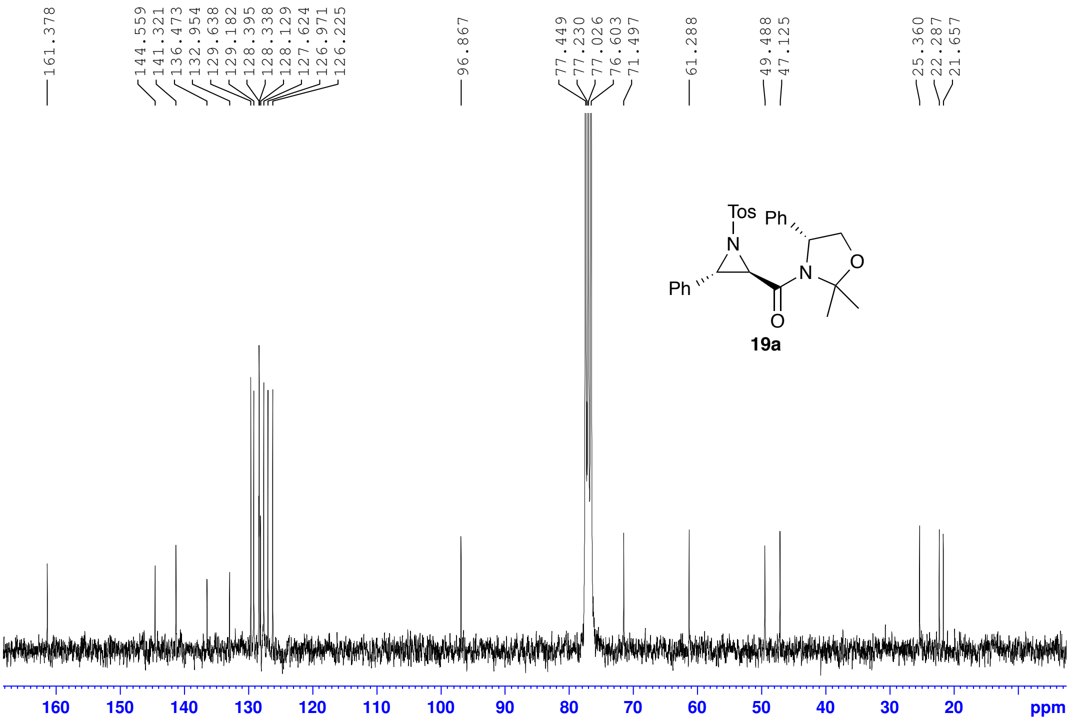
161.378
144.559 141.321 136.473 132.954 129.638 129.182 128.395 128.338 128.129 127.624 126.971 126.225
96.867
77.449 77.230 77.026 76.603 71.497
61.288
49.488 47.125
25.360 22.287 21.657
Tos
Ph
N
O
19a
160 150 140 130 120 110 100 90 80 70 60 50 40 30 20 ppm

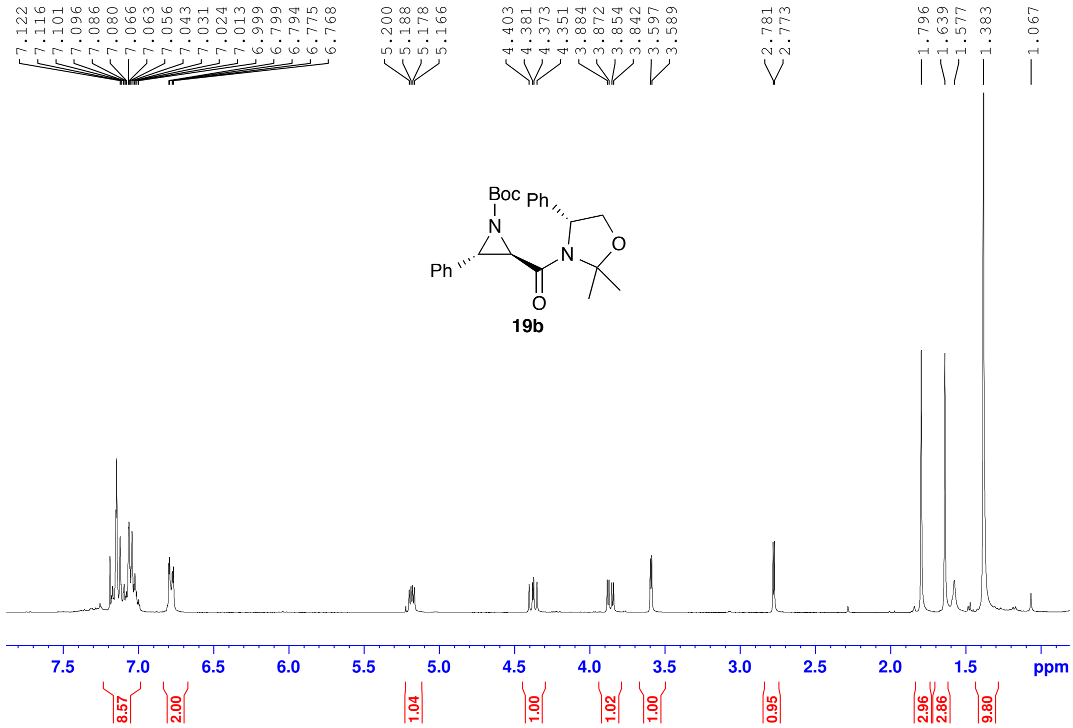
7.122 7.116 7.101 7.096 7.086 7.080 7.066 7.063 7.056 7.043 7.031 7.024 7.013 6.999 6.799 6.794 6.775 6.768
5.200 5.188 5.178 5.166
4.403 4.381 4.373 4.351 3.884 3.872 3.854 3.842 3.597 3.589
2.781 2.773
1.796 1.639 1.577 1.383
1.067
Boc
Ph
N
O
19b
7.5 7.0 6.5 6.0 5.5 5.0 4.5 4.0 3.5 3.0 2.5 2.0 1.5 ppm
8.57 2.00 1.04 1.00 1.02 1.00 0.95 2.96 2.86 9.80

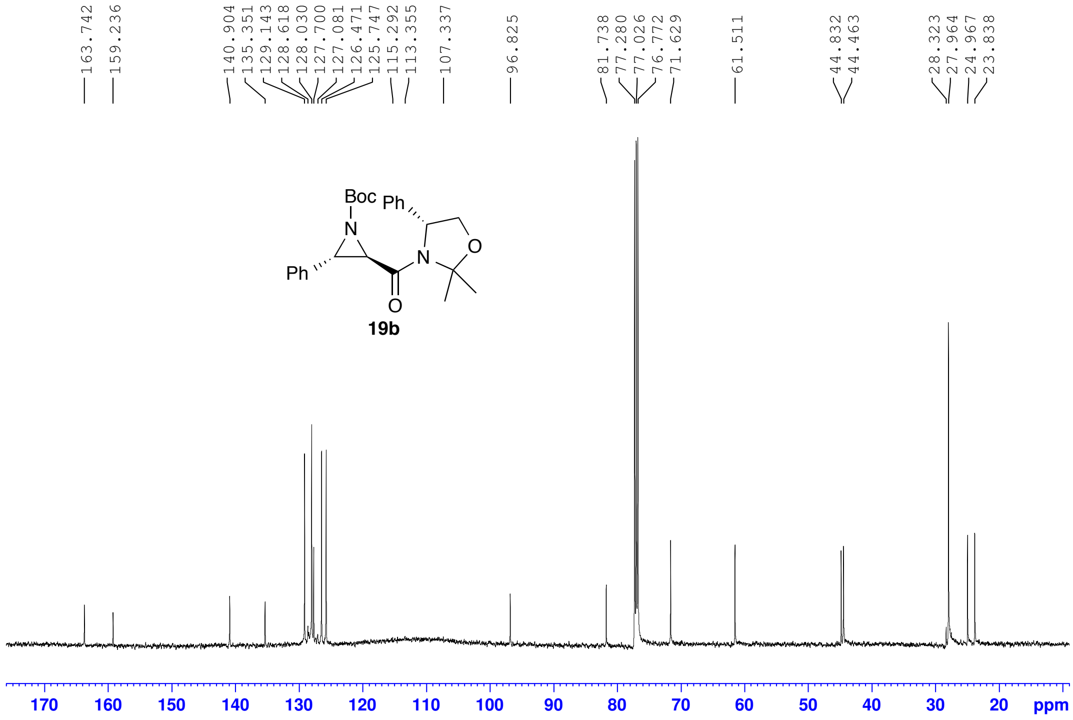
163.742 159.236
140.904 135.351 129.143 128.618 128.030 127.700 127.081 126.471 125.747 115.292 113.355
107.337
96.825
81.738 77.280 77.026 76.772 71.629
61.511
44.832 44.463
28.323 27.964 24.967 23.838
Boc
Ph
N
O
19b
170 160 150 140 130 120 110 100 90 80 70 60 50 40 30 20 ppm

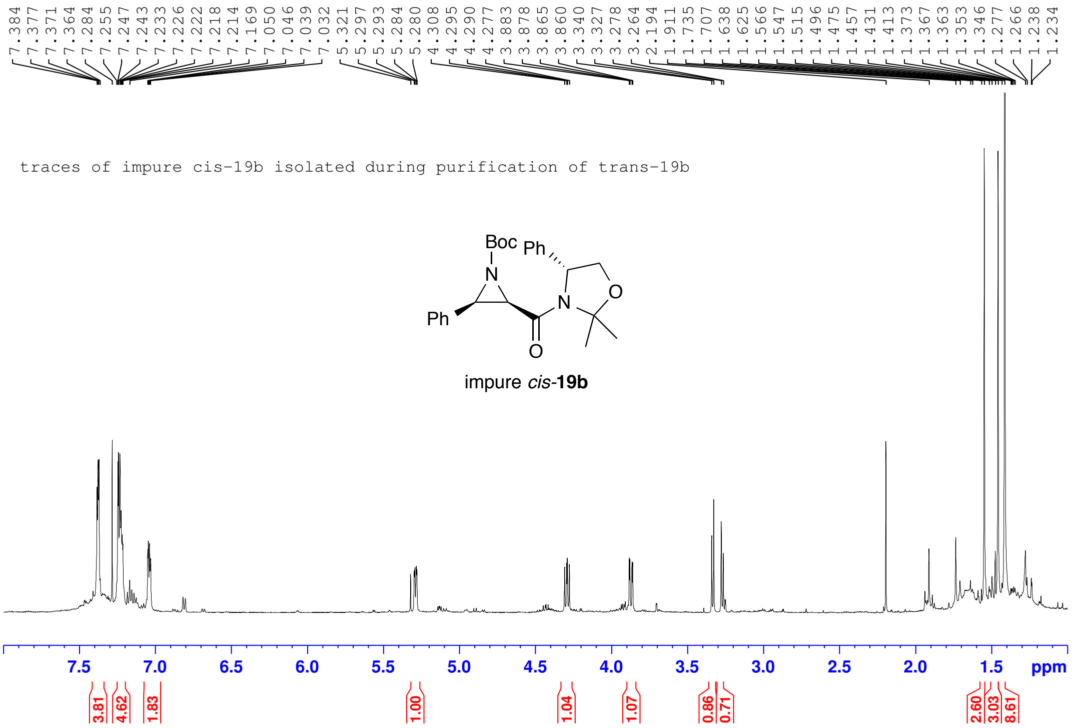
7.384 7.377 7.371 7.364 7.284 7.255 7.247 7.243 7.233 7.226 7.222 7.218 7.214 7.169 7.050 7.046 7.039 7.032 5.321 5.297 5.293 5.284 5.280 4.308 4.295 4.290 4.277 3.883 3.878 3.865 3.860 3.340 3.327 3.278 3.264 2.194 1.911 1.735 1.707 1.638 1.625 1.566 1.547 1.515 1.496 1.475 1.457 1.431 1.413 1.373 1.367 1.363 1.353 1.346 1.277 1.266 1.238 1.234
traces of impure cis-19b isolated during purification of trans-19b
Boc
Ph
N
O
Ph
N
O
impure cis-19b
7.5 7.0 6.5 6.0 5.5 5.0 4.5 4.0 3.5 3.0 2.5 2.0 1.5 ppm
3.81 4.62 1.83 1.00 1.04 1.07 0.86 0.71 2.60 3.03 8.61

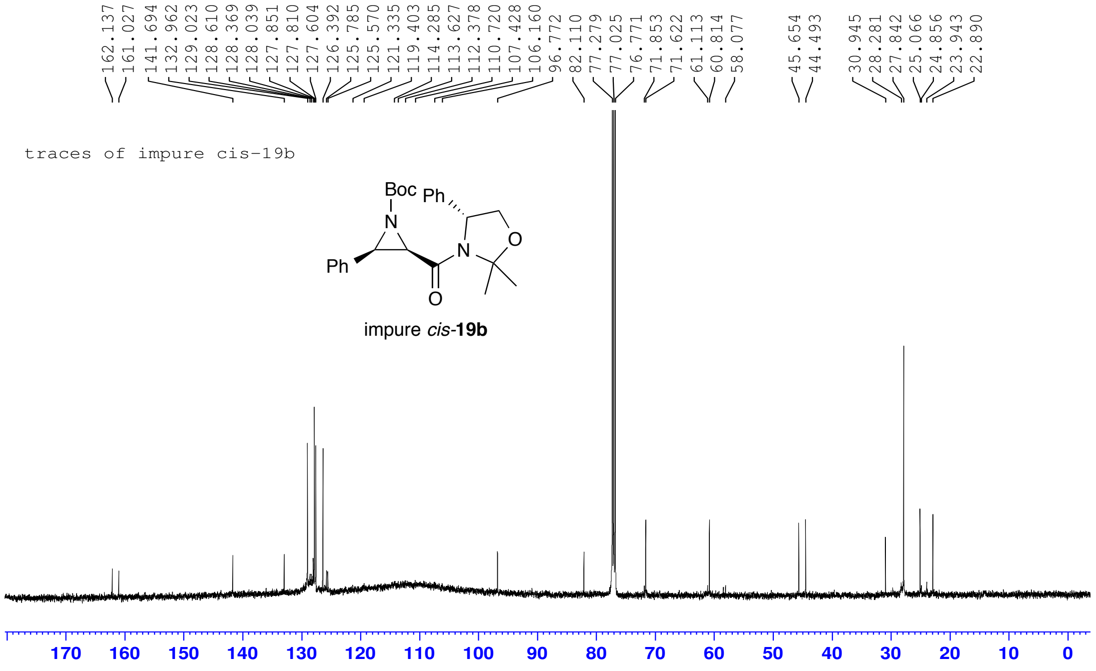
162.137 161.027 141.694 132.962 129.023 128.610 128.369 128.039 127.851 127.810 127.604 126.392 125.785 125.570 121.335 119.403 114.285 113.627 112.378 110.720 107.428 106.160 96.772 82.110 77.279 77.025 76.771 71.853 71.622 61.113 60.814 58.077 45.654 44.493 30.945 28.281 27.842 25.066 24.856 23.943 22.890
traces of impure cis-19b
Boc
Ph
N
O
Ph
N
O
impure cis-19b
170 160 150 140 130 120 110 100 90 80 70 60 50 40 30 20 10 0

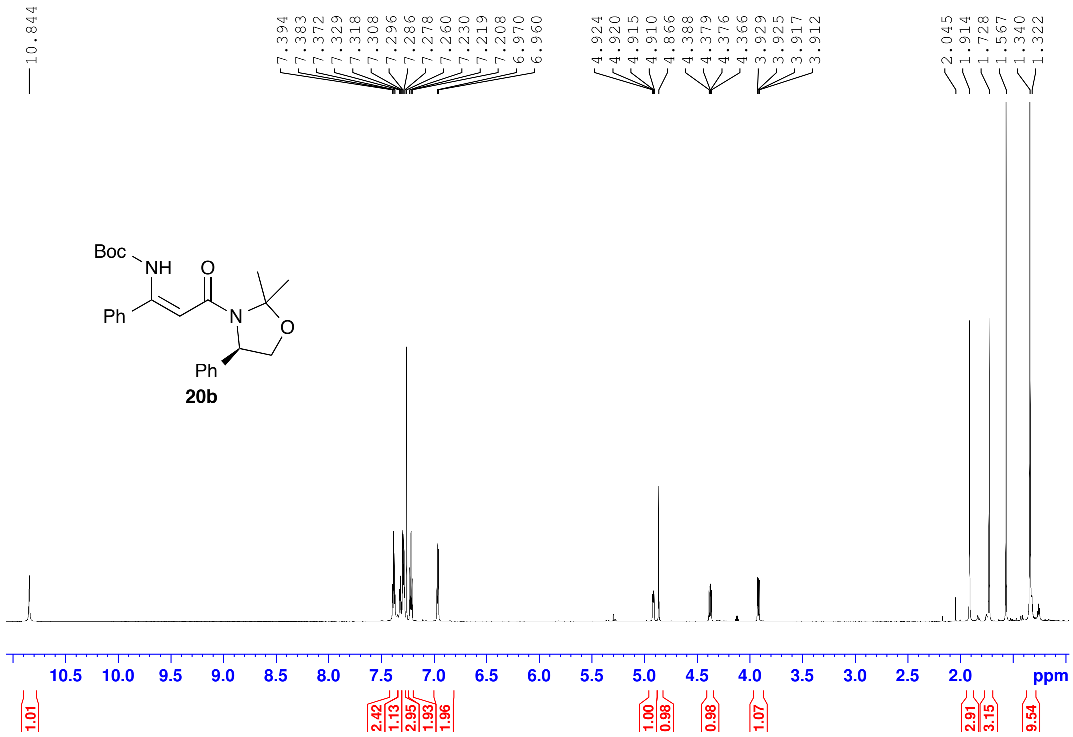
10.844
7.394 7.383 7.372 7.329 7.318 7.308 7.296 7.286 7.278 7.260 7.230 7.219 7.208 6.970 6.960
4.924 4.920 4.915 4.910 4.866 4.388 4.379 4.376 4.366 3.929 3.925 3.917 3.912
2.045 1.914 1.728 1.567 1.340 1.322
Boc
NH
O
Ph
N
O
Ph
20b
10.5 10.0 9.5 9.0 8.5 8.0 7.5 7.0 6.5 6.0 5.5 5.0 4.5 4.0 3.5 3.0 2.5 2.0 ppm
1.01 2.42 1.13 2.95 1.93 1.96 1.00 0.98 0.98 1.07 2.91 3.15 9.54

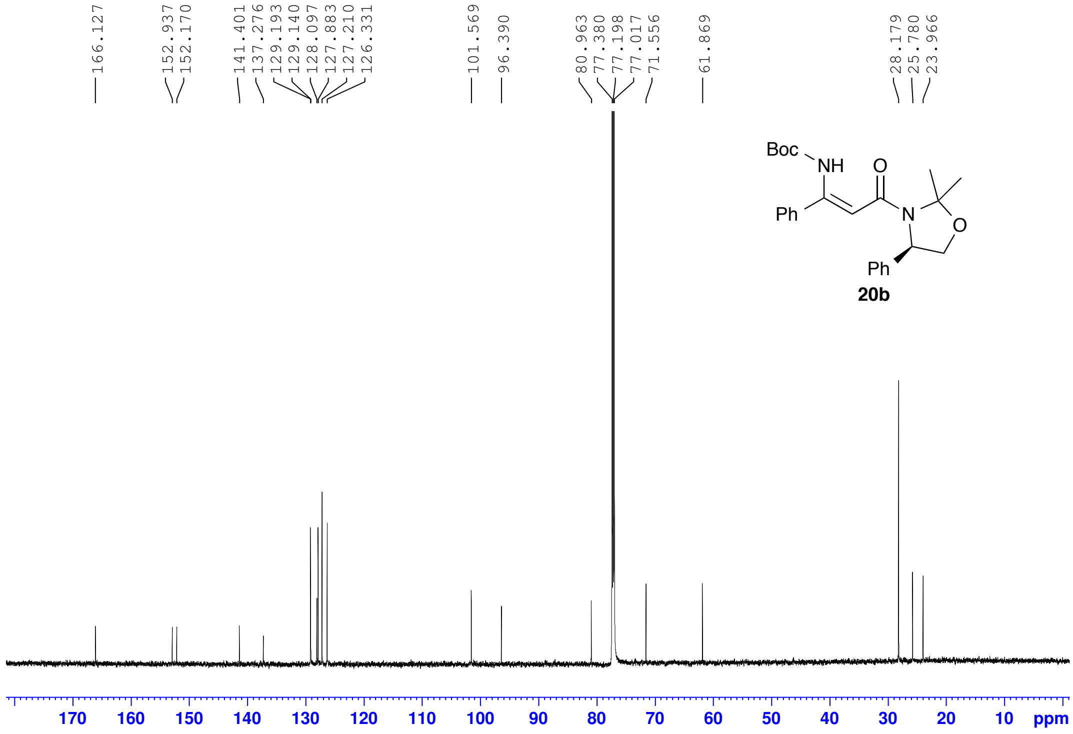
166.127
152.937 152.170
141.401 137.276 129.193 129.140 128.097 127.883 127.210 126.331
101.569 96.390
80.963 77.380 77.198 77.017 71.556
61.869
28.179 25.780 23.966
Boc
NH
O
Ph
N
O
Ph
20b
170 160 150 140 130 120 110 100 90 80 70 60 50 40 30 20 10 ppm

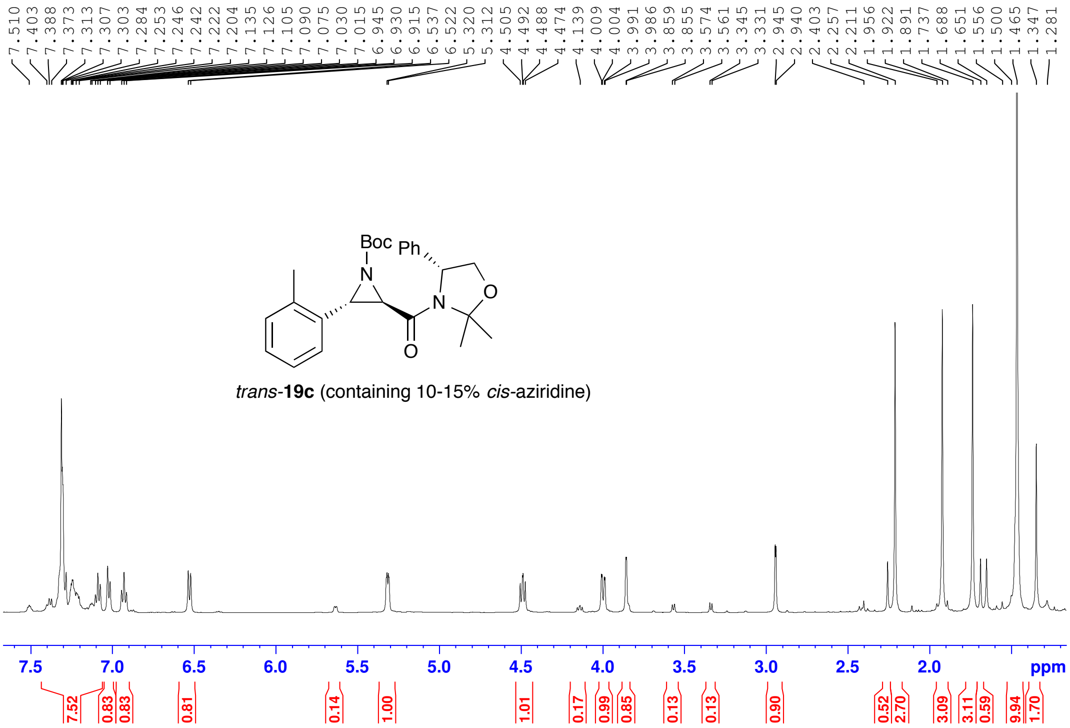
7.510 7.403 7.388 7.373 7.313 7.307 7.303 7.284 7.253 7.246 7.242 7.222 7.204 7.135 7.126 7.105 7.090 7.075 7.030 7.015 6.945 6.930 6.915 6.537 6.522 5.320 5.312 4.505 4.492 4.488 4.474 4.139 4.009 4.004 3.991 3.986 3.859 3.855 3.574 3.561 3.345 3.331 2.945 2.940 2.403 2.257 2.211 1.956 1.922 1.891 1.737 1.688 1.651 1.556 1.500 1.465 1.347 1.281
Boc
N
Ph
O
N
O
trans-**19c** (containing 10-15% *cis*-aziridine)
7.5 7.0 6.5 6.0 5.5 5.0 4.5 4.0 3.5 3.0 2.5 2.0 ppm
7.52 0.83 0.83 0.81 0.14 1.00 1.01 0.17 0.99 0.85 0.13 0.13 0.90 0.52 2.70 3.09 3.11 0.59 9.94 1.70

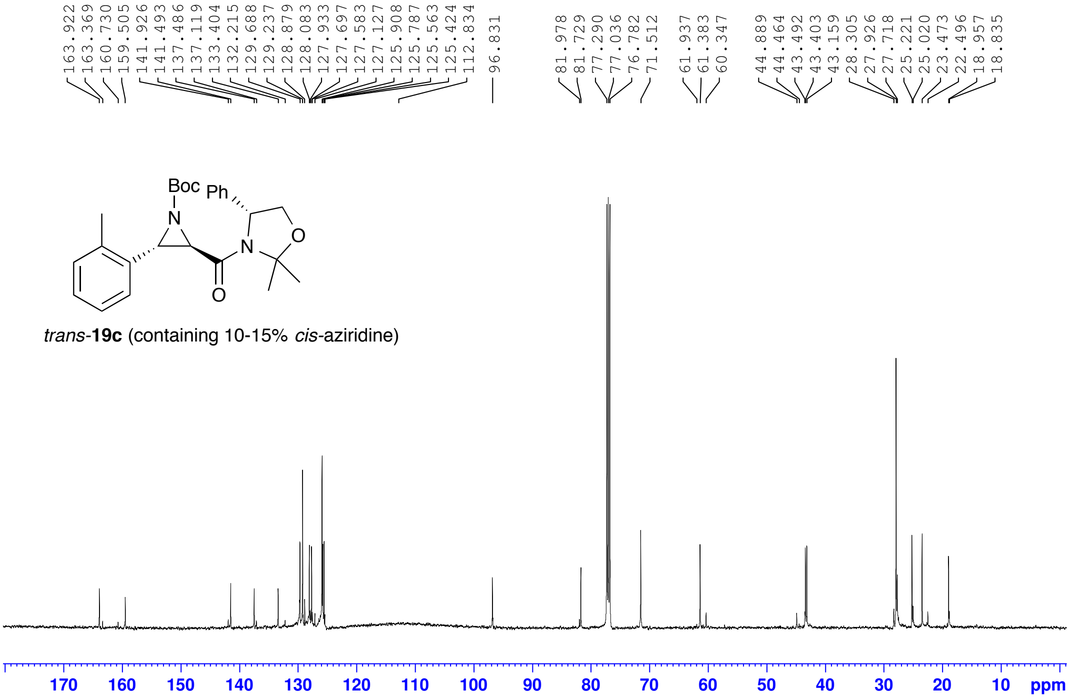
163.922 163.369 160.730 159.505 141.926 141.493 137.486 137.119 133.404 132.215 129.688 129.237 128.879 128.083 127.933 127.697 127.583 127.127 125.908 125.787 125.563 125.424 112.834 96.831 81.978 81.729 77.290 77.036 76.782 71.512 61.937 61.383 60.347 44.889 44.464 43.492 43.403 43.159 28.305 27.926 27.718 25.221 25.020 23.473 22.496 18.957 18.835
Boc
N
Ph
O
N
O
trans-**19c** (containing 10-15% *cis*-aziridine)
170 160 150 140 130 120 110 100 90 80 70 60 50 40 30 20 10 ppm

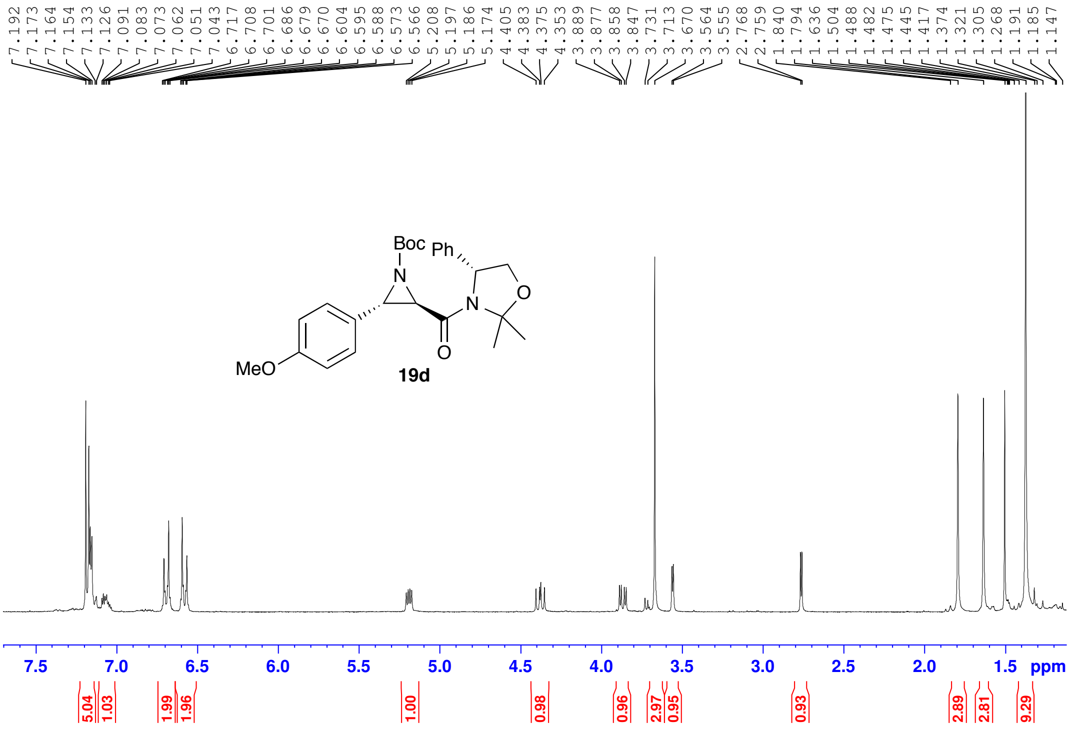
7.192 7.173 7.164 7.154 7.133 7.126 7.091 7.083 7.073 7.062 7.051 7.043 6.717 6.708 6.701 6.686 6.679 6.670 6.604 6.595 6.588 6.573 6.566 5.208 5.197 5.186 5.174 4.405 4.383 4.375 4.353 3.889 3.877 3.858 3.847 3.731 3.713 3.670 3.564 3.555 2.768 2.759 1.840 1.794 1.636 1.504 1.488 1.482 1.475 1.445 1.417 1.374 1.321 1.305 1.268 1.191 1.185 1.147
Boc
Ph
N
O
N
O
MeO
19d
7.5 7.0 6.5 6.0 5.5 5.0 4.5 4.0 3.5 3.0 2.5 2.0 1.5 ppm
5.04 1.03 1.99 1.96 1.00 0.98 0.96 2.97 0.95 0.93 2.89 2.81 9.29

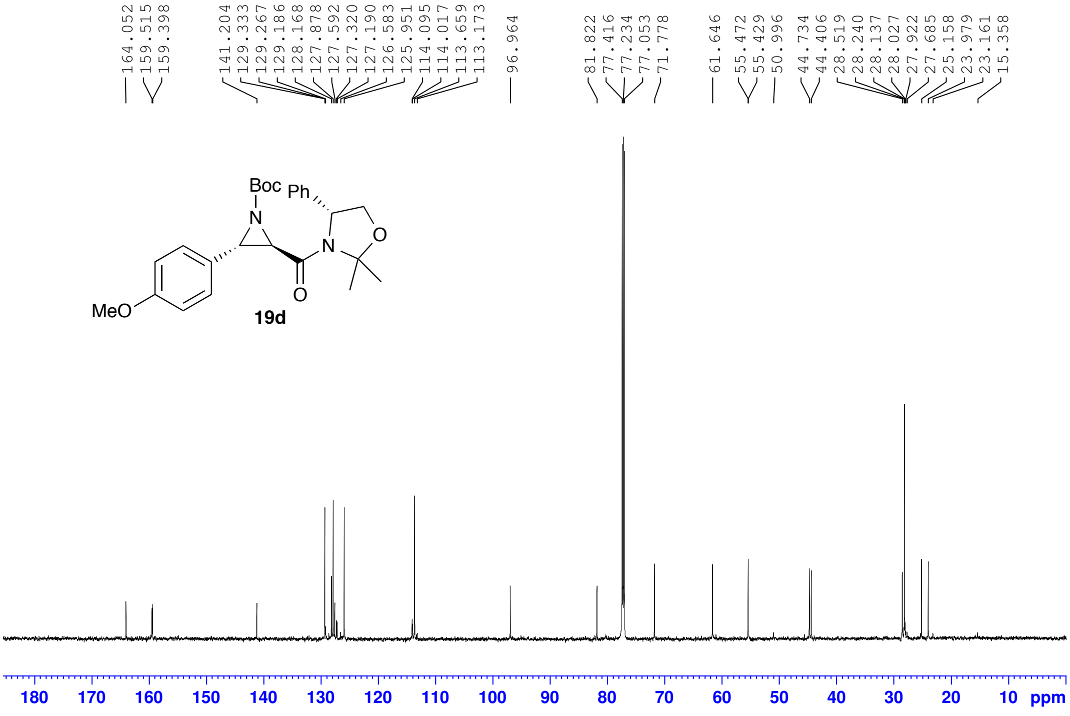
164.052 159.515 159.398 141.204 129.333 129.267 129.186 128.168 127.878 127.592 127.320 127.190 126.583 125.951 114.095 114.017 113.659 113.173 96.964 81.822 77.416 77.234 77.053 71.778 61.646 55.472 55.429 50.996 44.734 44.406 28.519 28.240 28.137 28.027 27.922 27.685 25.158 23.979 23.161 15.358
Boc
Ph
N
O
N
O
MeO
19d
180 170 160 150 140 130 120 110 100 90 80 70 60 50 40 30 20 10 ppm

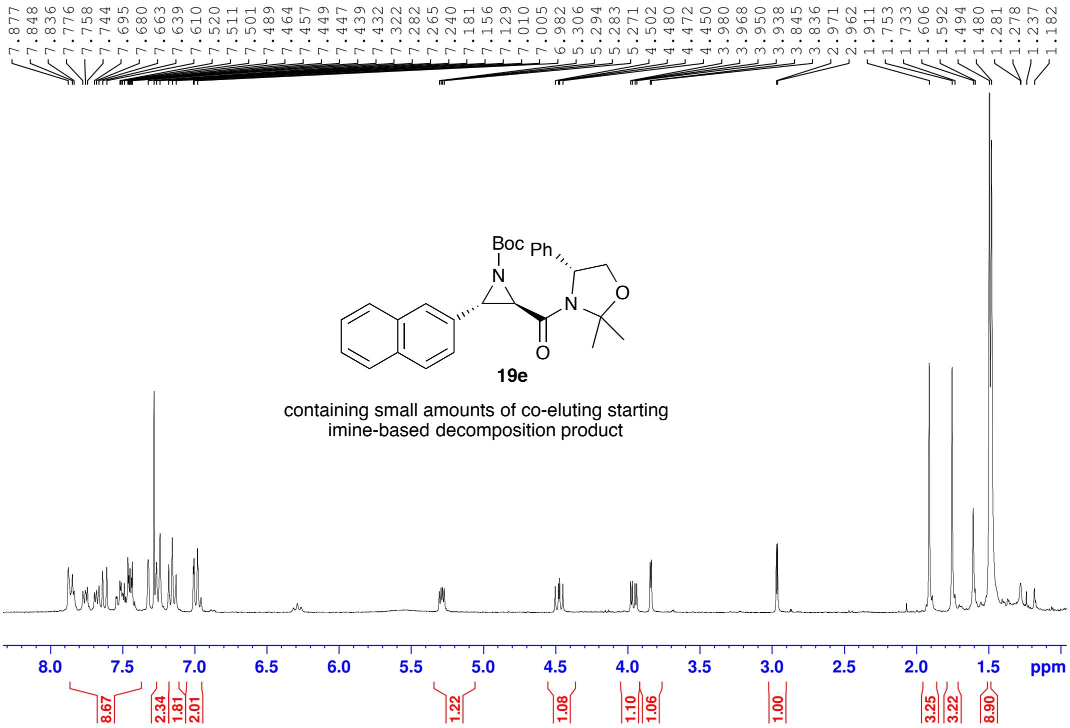
7.877 7.848 7.836 7.776 7.758 7.744 7.695 7.680 7.663 7.639 7.610 7.520 7.511 7.501 7.489 7.464 7.457 7.449 7.447 7.439 7.432 7.322 7.282 7.265 7.240 7.181 7.156 7.129 7.010 7.005 6.982 5.306 5.294 5.283 5.271 4.502 4.480 4.472 4.450 3.980 3.968 3.950 3.938 3.845 3.836 2.971 2.962 1.911 1.753 1.733 1.606 1.592 1.494 1.480 1.281 1.278 1.237 1.182
Boc
N
Ph
N
O
O
19e
containing small amounts of co-eluting starting
imine-based decomposition product
8.0 7.5 7.0 6.5 6.0 5.5 5.0 4.5 4.0 3.5 3.0 2.5 2.0 1.5 ppm
8.67 2.34 1.81 2.01 1.22 1.08 1.10 1.06 1.00 3.25 3.22 8.90

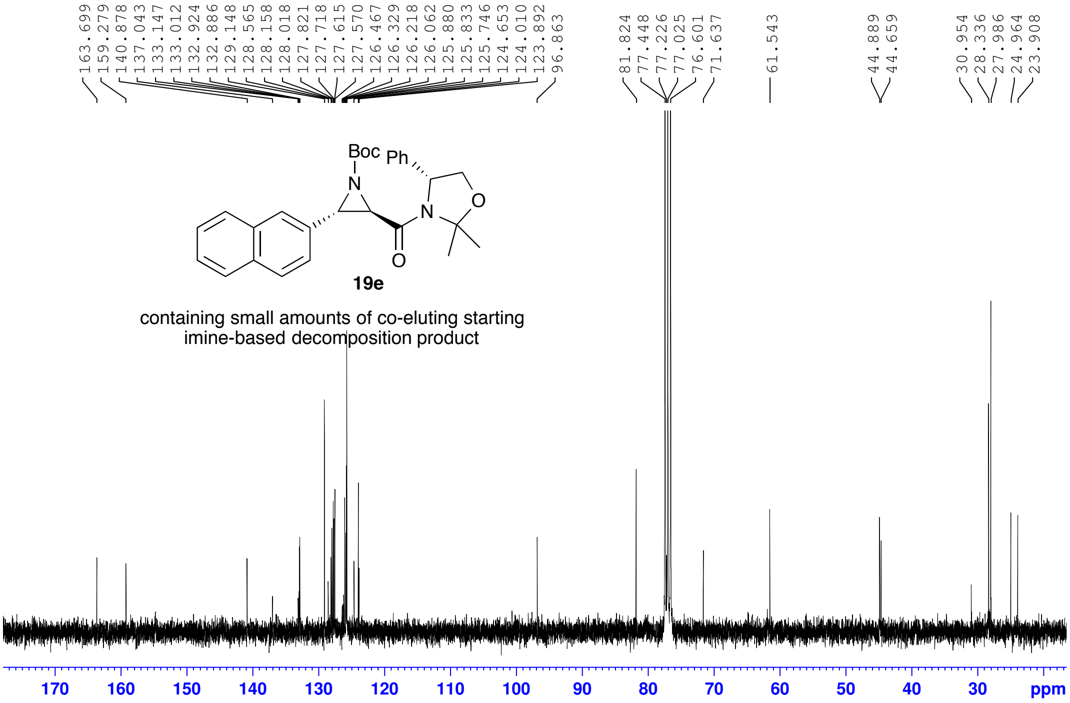
163.699 159.279 140.878 137.043 133.147 133.012 132.924 132.886 129.148 128.565 128.158 128.018 127.821 127.718 127.615 127.570 126.467 126.329 126.218 126.062 125.880 125.833 125.746 124.653 124.010 123.892 96.863 81.824 77.448 77.226 77.025 76.601 71.637 61.543 44.889 44.659 30.954 28.336 27.986 24.964 23.908
Boc
N
Ph
N
O
O
19e
containing small amounts of co-eluting starting
imine-based decomposition product
170 160 150 140 130 120 110 100 90 80 70 60 50 40 30 ppm

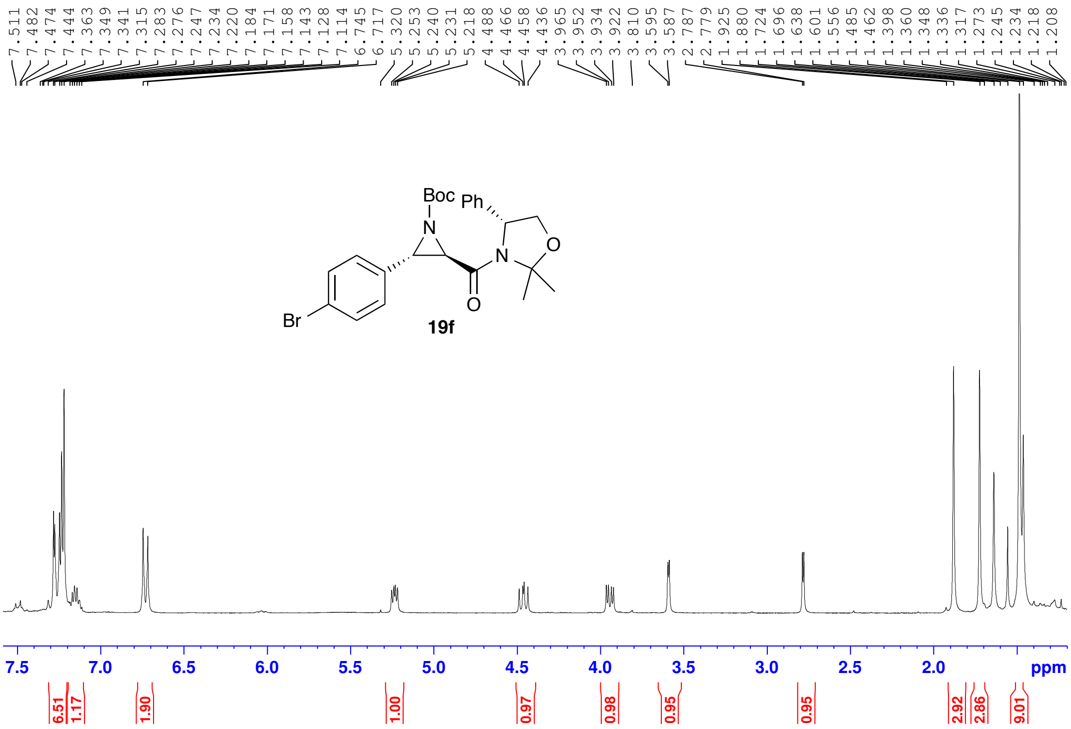
7.511 7.482 7.474 7.444 7.363 7.349 7.341 7.315 7.283 7.276 7.247 7.234 7.220 7.184 7.171 7.158 7.143 7.128 7.114 6.745 6.717 5.320 5.253 5.240 5.231 5.218 4.488 4.466 4.458 4.436 3.965 3.952 3.934 3.922 3.810 3.595 3.587 2.787 2.779 1.925 1.880 1.724 1.696 1.638 1.601 1.556 1.485 1.462 1.398 1.360 1.348 1.336 1.317 1.273 1.245 1.234 1.218 1.208
Boc
Ph
N
O
Br
19f
7.5 7.0 6.5 6.0 5.5 5.0 4.5 4.0 3.5 3.0 2.5 2.0 ppm
6.51 1.17 1.90 1.00 0.97 0.98 0.95 0.95 2.92 2.86 9.01

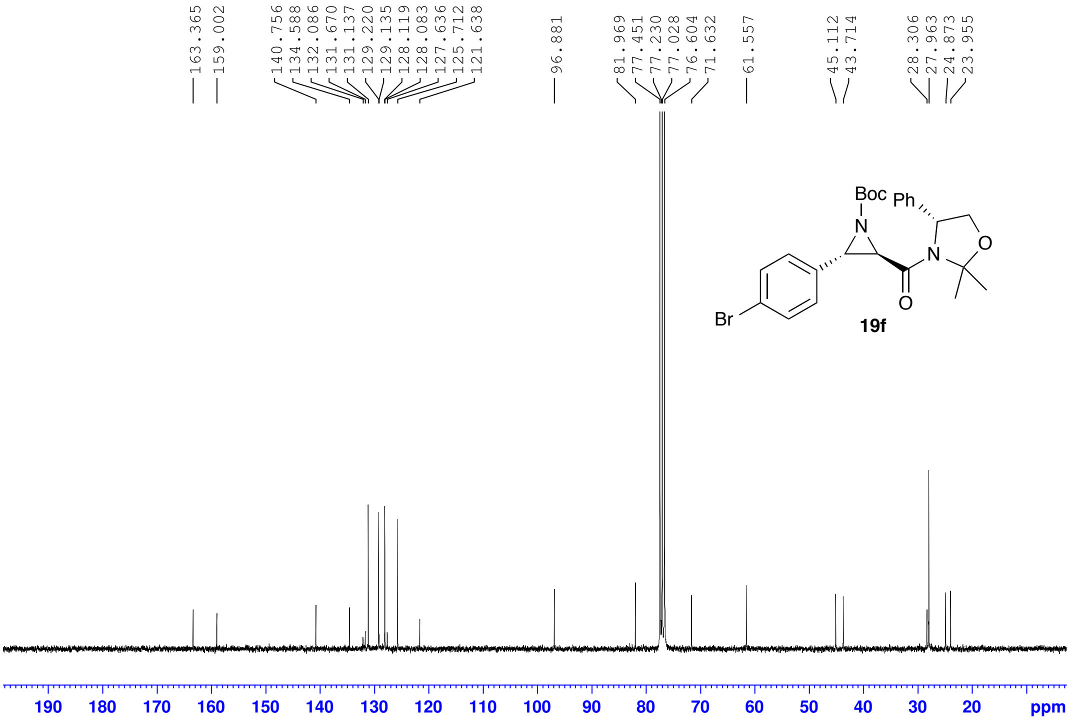
163.365 159.002 140.756 134.588 132.086 131.670 131.137 129.220 129.135 128.119 128.083 127.636 125.712 121.638 96.881 81.969 77.451 77.230 77.028 76.604 71.632 61.557 45.112 43.714 28.306 27.963 24.873 23.955
Boc
Ph
N
O
Br
19f
190 180 170 160 150 140 130 120 110 100 90 80 70 60 50 40 30 20 ppm

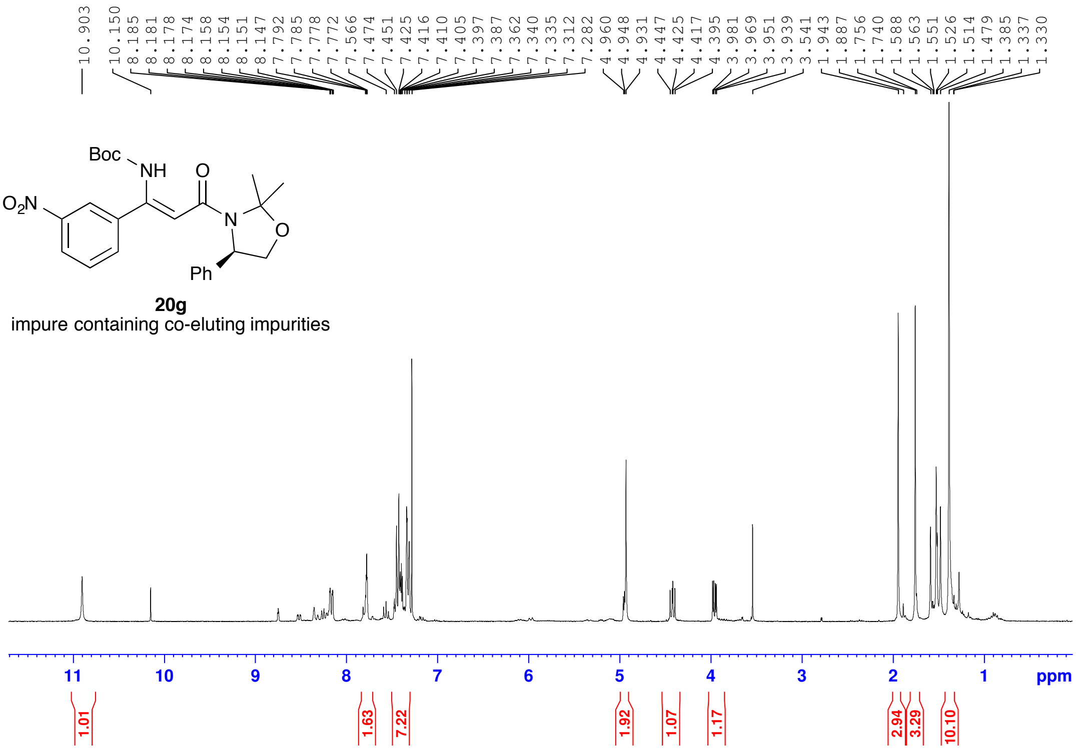
10.903
10.150
8.185
8.181
8.178
8.174
8.158
8.154
8.151
8.147
7.792
7.785
7.778
7.772
7.566
7.474
7.451
7.425
7.416
7.410
7.405
7.397
7.387
7.362
7.340
7.335
7.312
7.282
4.960
4.948
4.931
4.447
4.425
4.417
4.395
3.981
3.969
3.951
3.939
3.541
1.943
1.887
1.756
1.740
1.588
1.563
1.551
1.526
1.514
1.479
1.385
1.337
1.330
Boc
NH
O
O_2N
N
O
Ph
20g
impure containing co-eluting impurities
11 10 9 8 7 6 5 4 3 2 1 ppm
1.01
1.63
7.22
1.92
1.07
1.17
2.94
3.29
10.10

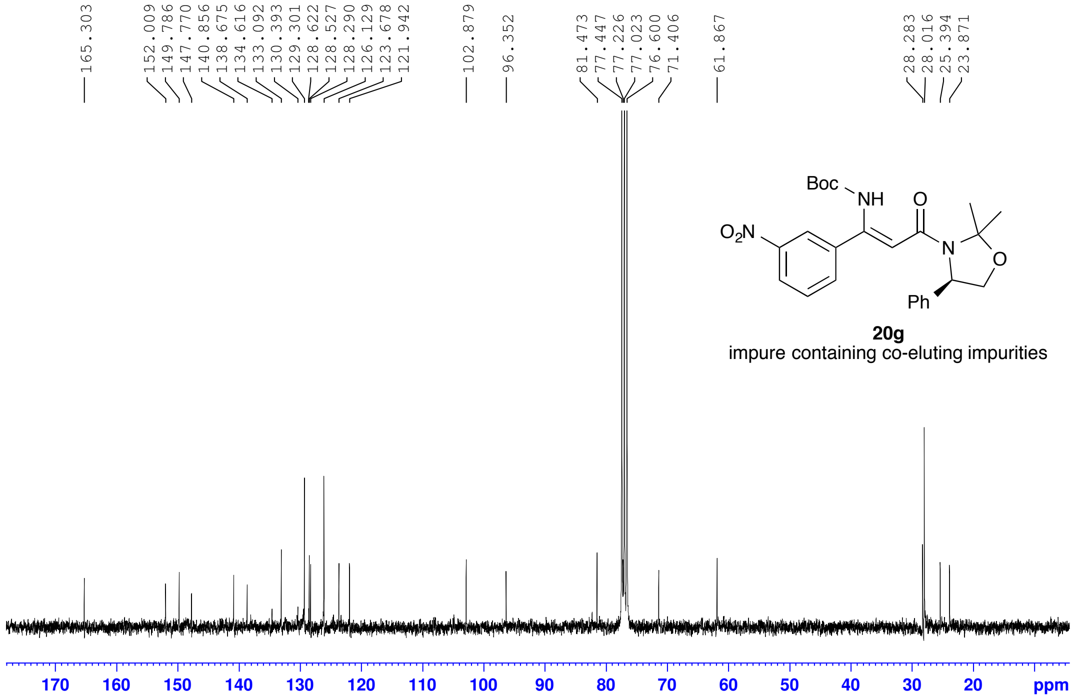
165.303
152.009
149.786
147.770
140.856
138.675
134.616
133.092
130.393
129.301
128.622
128.527
128.290
126.129
123.678
121.942
102.879
96.352
81.473
77.447
77.226
77.023
76.600
71.406
61.867
28.283
28.016
25.394
23.871
Boc
NH
O
O_2N
N
O
Ph
20g
impure containing co-eluting impurities
170 160 150 140 130 120 110 100 90 80 70 60 50 40 30 20 ppm
